# VectorNet: collaborative mapping of arthropod disease vectors in Europe and surrounding areas since 2010

**DOI:** 10.2807/1560-7917.ES.2023.28.26.2200666

**Published:** 2023-06-29

**Authors:** G.R. William Wint, Thomas Balenghien, Eduardo Berriatua, Marieta Braks, Cedric Marsboom, Jolyon Medlock, Francis Schaffner, Wim Van Bortel, Neil Alexander, Bulent Alten, Ewelina Czwienczek, Sofie Dhollander, Els Ducheyne, Celine M. Gossner, Kayleigh Hansford, Guy Hendrickx, Hector Honrubia, Tom Matheussen, Andrei Daniel Mihalca, Dusan Petric, Jane Richardson, Hein Sprong, Veerle Versteirt, Olivier Briet

**Affiliations:** 1 Environmental Research Group Oxford Ltd, c/o Department of Biology, Oxford, United Kingdom; 2 CIRAD, UMR ASTRE, Rabat, Morocco; 3 ASTRE, University of Montpellier, CIRAD, INRAE, Montpellier, France; 4 Unité Microbiologie, immunologie et maladies contagieuses, Institut Agronomique et Vétérinaire Hassan II, Rabat, Morocco; 5 Departamento de Sanidad Animal, Facultad de Veterinaria, Regional Campus of International Excellence “Campus Mare Nostrum”, Universidad de Murcia, Murcia, Spain; 6 Centre for Zoonoses and Environmental Microbiology, National Institute for Public Health and the Environment, Bilthoven, The Netherlands; 7 Avia-GIS, Agro-Veterinary Information and Analysis, Zoersel, Belgium; 8 Medical Entomology & Zoonoses Ecology, UK Health Security Agency, Porton Down, United Kingdom; 9 Francis Schaffner Consultancy, Riehen, Switzerland; 10 Unit Entomology and the Outbreak Research Team, Institute of Tropical Medicine, Antwerp, Belgium; 11 Hacettepe University, Faculty of Science, Department of Biology, Ecology Division, VERG Laboratories, Beytepe, Ankara, Turkey; 12 European Food Safety Authority (EFSA), Parma, Italy; 13 Disease Programme Unit, European Centre for Disease Prevention and Control, Stockholm, Sweden; 14 Johnson and Johnson, Beerse, Belgium; 15 Public Health Functions Unit, European Centre for Disease Prevention and Control, Stockholm, Sweden; 16 Department of Parasitology and Parasitic Diseases, University of Agricultural Sciences and Veterinary Medicine of Cluj-Napoca, Cluj-Napoca, Romania; 17 Parasitology Consultancy Group, Corușu, Romania; 18 Faculty of Agriculture, University of Novi Sad, Serbia; 19 Centre for Infectious Disease Control, National Institute for Public Health and the Environment (RIVM), Bilthoven, The Netherlands; 20 Agentschap voor Natuur en Bos, Havenlaan 88, 1000 Brussels, Belgium

**Keywords:** arthropod vectors, vector-borne disease, VectorNet, maps, distributions, risk assessment, vector surveillance, network

## Abstract

**Background:**

Arthropod vectors such as ticks, mosquitoes, sandflies and biting midges are of public and veterinary health significance because of the pathogens they can transmit. Understanding their distributions is a key means of assessing risk. VectorNet maps their distribution in the EU and surrounding areas.

**Aim:**

We aim to describe the methodology underlying VectorNet maps, encourage standardisation and evaluate output.

**Method:**

s: Vector distribution and surveillance activity data have been collected since 2010 from a combination of literature searches, field-survey data by entomologist volunteers via a network facilitated for each participating country and expert validation. Data were collated by VectorNet members and extensively validated during data entry and mapping processes.

**Results:**

As of 2021, the VectorNet archive consisted of ca 475,000 records relating to > 330 species. Maps for 42 species are routinely produced online at subnational administrative unit resolution. On VectorNet maps, there are relatively few areas where surveillance has been recorded but there are no distribution data. Comparison with other continental databases, namely the Global Biodiversity Information Facility and VectorBase show that VectorNet has 5–10 times as many records overall, although three species are better represented in the other databases. In addition, VectorNet maps show where species are absent. VectorNet’s impact as assessed by citations (ca 60 per year) and web statistics (58,000 views) is substantial and its maps are widely used as reference material by professionals and the public.

**Conclusion:**

VectorNet maps are the pre-eminent source of rigorously validated arthropod vector maps for Europe and its surrounding areas.

**Key public health message**

**What did you want to address in this study?**

We wished to present the VectorNet surveillance data and map archive, which has been compiled since 2010, to provide public health professionals, academics and the public with state-of-the-art spatial distribution maps of the mosquito, tick, sandfly and biting midge species that transmit a wide range of human and animal diseases. An important objective of the paper is to provide practitioners with details of the methodology used and broad interpretations of its results.

**What have we learnt from this study?**

The combination of literature search, contribution from volunteer network members, expert opinion and iterative validation provides a reliable and comprehensive source of information that is more complete than any other data source with similar scope. As a result, the VectorNet maps are the ‘go-to’ resource for vector distributions in Europe and its surrounding areas.

**What are the implications of your findings for public health?**

Well-structured and thoroughly expert-validated network-based data collection can provide reliable vector distribution data, which are useful for the development of a public-health strategy for vector-borne disease mitigation.

## Introduction

Vector-borne diseases (VBD) include a wide range of infectious diseases of humans and other animals. Some VBD have (re)emerged in Europe and its surrounding areas: mosquito-borne diseases like malaria [1,2] chikungunya, dengue [3,4], Zika [5-8] and West Nile virus infection [9-11]. Others have been endemic in Europe for many years, prominent examples being tick-borne diseases such as Lyme borreliosis and tick-borne encephalitis, sandfly-borne diseases such as leishmaniasis [12] and biting-midge-borne diseases such as bluetongue and Schmallenberg virus disease [13,14] in livestock. Knowing the spatial distribution of competent vectors helps understand the risk of these diseases, as vector presence is a prerequisite for vector-borne transmission.

The European Centre for Disease Prevention and Control (ECDC) started mapping vector distributions in Europe to enable continental VBD risk assessment with the Tiger Maps project (2008–2009), focusing on *Aedes albopictus* [15], and the VBORNE project (2008), reviewing surveillance practices of tick, mosquito, sandfly and rodent vectors. Subsequently, the VBORNET project (2010–2013) standardised protocols and mapped a range of priority tick, mosquito and sandfly vector species. These species were identified by expert consultation at the Nomenclature of Territorial Units for Statistics Level 3 (NUTS3) using data from literature searches and data submitted by a network of volunteer entomologists. VectorNet (2014–2018), commissioned by ECDC jointly with the European Food Safety Authority (EFSA), expanded to include vector sampling, and extended mapping coverage to incorporate North Africa, the Near East, and the western part of Central Asia and added biting midges as a vector group. VectorNet also provides networking support, expert advice and scientific opinions, e.g [16]. A second VectorNet phase (beginning in 2019) has added target species to make a total of 58 priority species of public and veterinary health importance (Table 1). The network has been strengthened with VectorNet Entomological Network (VEN) national contact points who facilitate information transfer between national colleagues and project members. VectorNet now includes recording of surveillance activities but no longer commissions field sampling.

**Table 1:** Current VectorNet priority species, 2019–2023

Biting midges	Ticks	Sandflies	Mosquitoes	
***Culicoides chiopterus***	***Dermacentor reticulatus***	***Phlebotomus alexandri***	***Aedes aegypti***	***An. messeae* ^5^**
***Cu. dewulfi***	***Hyalomma lusitanicum***	***P. ariasi***	***Ae. albopictus***	***An. plumbeus***
***Cu. imicola***	***H. marginatum***	*P. langeroni*	***Ae. atropalpus***	***An. sacharovi* ^5^**
***Cu. kingi***	***Ixodes persulcatus***	*P. major* s.l.	***Ae. caspius***	***An. superpictus***
***Cu. lupicaris* ^1^**	***I. ricinus***	***P. mascittii***	***Ae. detritus* ^3^**	***Coquillettidia richiardii***
***Cu. newsteadi* s.l.**	***Ornithodoros erraticus***	***P. neglectus***	***Ae. coluzzii* ^3^**	*Culex antennatus*
***Cu. obsoletus* s.l.^2^**	***Rhipicephalus sanguineus* s.l.**	***P. papatasi***	***Ae. japonicus***	***Cx.modestus***
***Cu. pulicaris* s.l.^1^**		***P. perfiliewi***	***Ae. koreicus***	*Cx. perexiguus*
***Cu. punctatus* s.l.**		***P. perniciosus***	***Ae. vexans* s.l.^4^**	***Cx. pipiens* ^6^**
***Cu. scoticus* ^2^**		***P. sergenti***	***Ae. vexans vexans* ^4^**	*Cx. theileri*
		***P. similis***	***Anopheles atroparvus* ^5^**	***Cx. torrentium* ^6^**
		***P. tobbi***	*An. claviger*	*Cx. tritaeniorhynchus*
			***An. labranchiae* ^5^**	*Cx. univittatus*
			***A. maculipennis* s.l.^5^**	*Culiseta annulata*
			***A. maculipennis* s.s.^5^**	

SL: sensu latu.

^1–6^: Species with matching superscripted numbers are jointly mapped complexes. Priority species were defined by expert consultation during the VBORNE, VBORNET and VectorNet projects. Maps of species in bold are available online [34]. Data for the others can be requested.

While many studies have mapped vectors at subnational, national, continental or global level [17-19], there are few repositories that contain standardised data. Besides VectorNet, these include VectorBase (https://vectorbase.org/vectorbase/app/), the ENHanCEd Infectious Diseases Database (EID2) [20], focussing on pathogens and vectors and the Global Biodiversity Information Facility (GBIF) [21]. There are also a significant number of citizen science initiatives such as Mosquito Alert, Tekenradar and the United Kingdom (UK) Health Security Agency Tick Surveillance Scheme. While data rich, these are not yet comparable with the standardised databases as the sampling effort is not yet sufficiently defined.

This paper aims to provide details of the data standardisation and methodology underlying the mapping of the VectorNet priority species, to present and summarise the maps, and using data collected up to 2021, compare them with the other standardised databases and assess their use and impact. This paper also identifies potential future improvements to the maps.

## Methods

### Data collection, validation and mapping

Within the VectorNet project, vector group leaders (VGLs) coordinate the collection, validation and mapping of data on the distribution of mosquitoes, ticks, sandflies and biting midges (Table 1). Each VGL is responsible for a single vector group. Data are collected from several sources: (i) individual researchers listed in Supplementary Table S1; (ii) published literature using strictly defined search protocols, set out in detail in Supplementary Table S2, which reflect the different sampling methodologies used for each group; (iii) national and regional surveillance databases; and (iv) in 2014–2018, standardised field sampling designed to refine distributions and fill gaps.

In Tiger Maps and VBORNET, data were entered into spreadsheets, while VectorNet phase 1 used an online data entry platform. In VectorNet phase 2, VGLs use a macro-driven spreadsheet-based system for column and content description with look-up tables to populate dropdown lists, tools to generate location codes and error-trapping algorithms. These are detailed in Supplementary Table S3. From the end of VectorNet phase 1, data collection expanded from polygon-based presence/absence data to recording point location occurrence, including abundance, seasonality and sampling effort. The metrics used to specify these variables are also set out in detail in Supplementary Table S3. In the latest phase of the project, the data entry was repeated for approximately 1% of these records, compared with the original data and quality metrics calculated. The quality indicators used are given in Supplementary Table S4. The database thus contains both polygon-based distribution status data and (more) recent point-based abundance data.

The spatial units at which vector distribution status is determined are based on NUTS3 [22] and Global Administrative Unit Layers Second-Level (GAUL 2) [23], where the NUTS system does not reach. The mapped species distribution categories within each polygon are as follows: (i) ‘present’ or ‘established’ are confirmed or assumed by expert opinion to be established; (ii) ‘introduced’ is recorded as present but not considered by expert opinion to be established; (iii) ‘anticipated absent’ is inferred as absent by expert opinion or known environmental constraints; (iv) ‘absent’ or ‘observed absent’ are recorded as absent; (v) ‘no data’ confirms that no data are available; (vi) ‘unknown’ means there is no information on data availability, or data are unreliable. Expert opinion here means assessed by members of the VectorNet network and validated and reconciled for each vector group by a dedicated project member. In the 'introduced’ and ‘present’ categories, the vectors may not be present everywhere within the polygon as they may contain unsuitable areas. ‘Introduced’ has only been used for ticks and invasive mosquitos. Ticks are likely to be carried to locations by a host but are not recorded throughout the year. For mosquitoes, the first record of an invasive species is categorised as introduced if there is no evidence of overwintering, unless the record consists of multiple records throughout a year. An ‘introduced’ record reverts to a display of ‘absent’ after 5 years if searches have found no further presence records. VectorNet aims to depict current vector distribution, therefore only data after 1980 are collated. For vector species complexes (sibling or cryptic species), data are reported at complex level (e.g. *Culicoides obsoletus* s.l., *Anopheles maculipennis* s.l.) unless complex members are reported based on reliable identification methods (i.e. molecular).

The spreadsheet data are subjected to further spatial error trapping routines (to validate coordinates against named locations, and extent limits) and then visualised with ECDC’s Map Maker [24]. This combines current polygon distribution status with new point data and any suggested status changes according to the hierarchical dominance of the reported status, which is as follows: present, introduced, absent, anticipated absent, no data and unknown. These changes are then validated by the VGLs based on their expert knowledge, and, together with any corrections, incorporated into the ECDC VectorNet database. To facilitate change assessment, all data records are retained. This updated database is subsequently used to map distributions in Feature Manipulation Engine (FME) software. Because polygon sizes vary considerably and, for some countries, are sometimes difficult to visualise at pan-European scale, they are aggregated to mapping polygons, assigning the dominant distribution status to any aggregated polygon. The generated maps are then re-circulated to the VGLs for approval before posting online. If the administrative units are occasionally changed by the national authorities, the mapping polygons are also modified. If they are split then the original polygon entry is duplicated to ‘children’ and point location statuses are assigned as appropriate.

The data acquisition, entry and validation cycle is repeated bi-annually, with new maps posted in late spring and autumn.

### Coverage and completeness of the database

A preliminary assessment of VectorNet record numbers, coverage and accuracy in relation to other continental databases such as GBIF, VectorBase and EID2 was undertaken. Of these other databases, either GBIF or VectorBase was found to have the most records for each of the species mapped by VectorNet. The March 2021 VectorNet distribution maps were therefore compared (and overlaid) with point records from the other database with the most records per species (downloaded in July 2021), and the number of VectorNet polygons with VectorBase or GBIF records were counted. Comparisons with modelled distributions was not made as many, if not most, continental scale models incorporate VectorNet data in their training datasets [25,26].

### Surveillance activities

In late 2020, the VGLs asked the VEN to record publicly funded and reported surveillance activities for each vector group in their country. Information for each polygon was requested on an annual basis for 2015 and 2016, and a monthly timescale for 2017–2019. Surveillance efforts were coded according to sampling methods/effort and species targeted (preamble to Supplementary Figures 1–5). Two sets of maps were produced: one showing the number of calendar months surveyed during 2017–2019 and the other showing the surveillance effort, weighted according to the sampling method used. The VGLs screened these for anomalies and corrected errors.

The surveillance activity and vector distribution maps were compared in order to check where surveillance was reported but distributions were not, and vice versa (preamble to Supplementary Figure S6). As surveillance maps are by vector group, while most distribution maps are for single species, the latter were aggregated to identify the polygons where at least 80% of the mapped species within a vector group had data.

Finally, and indication of the scientific impact of the online maps was assessed via a non-exhaustive literature search (which is likely to underestimate impact), combining ‘dimensions’ and ‘publish or perish’ software searches using Google Scholar, Web of Science and PubMed with the project names VBORNE, VBORNET and VectorNet as search terms.

## Results

The cumulative archive contains > 273,000 records with polygon presence/absence records or point locations with reported numbers and sample methods (abundance) covering 322 vector species (Table 2). Sampling effort details have been more rigorously defined since 2019 (Supplementary Table S3).

**Table 2:** Data and record numbers for VectorNet priority species, 2010–2021

Group and year	Recorded species number	Mapped species number	Total record number	Point record number	Abundance record number	% polygons mapped spp.	Data polygon number
Invasive mosquitoes							
2010	5	5	370	0	0	5	1,523
2013	5	5	749	0	0	47	1,587
2017/18	5	5	1,902	1,107	0	39	5,070
2019	5	5	7,116	3,752	3,859	40	5,070
2021	5	5	36,585	20,694	19,541	56	2,964
Native mosquitoes							
2010	0	0	0	0	0	0	1,523
2013	3	3	376	0	0	24	1,587
2017/18	33	10	3,102	1,601	0	5	5,070
2019	53	10	10,739	7,837	9,393	7	5,070
2021	110	10	32,671	21,688	20,961	19	2,964
All mosquitoes							
2010	5	5	370	0	0	24	1,523
2013	8	8	1,125	0	0	38	1,587
2017/18	38	15	5,004	2,708	0	17	5,070
2019	58	15	17,855	11,589	13,252	18	5,070
2021	115	15	69,256	42,382	40,502	31	2,964
Biting midges							
2010	0	0	0	0	0	0	1,523
2013	0	0	0	0	0	0	1,587
2017/18	58	10	27,080	18,611	27,080	3	5,070
2019	98	10	33,474	24,935	21,980	12	5,070
2021	127	10	77,381	65,833	58,376	37	2,964
Sandflies							
2010	10	10	124	0	0	1	1,523
2013	10	10	1,206	0	0	76	1,587
2017/18	15	10	40,246	40,042	0	38	5,070
2019	18	10	42,670	40,409	3,033	38	5,070
2021	40	10	89,062	54,903	15,026	59	2,964
Ticks							
2010	6	6	0	0	0	0	1,523
2013	6	6	234	0	0	15	1,587
2017/18	32	7	8,181	6,498	0	13	5,070
2019	34	7	20,857	12,752	11,173	13	5,070
2021	40	7	37,420	13,814	15,872	25	2,964
All							
2010	21	21	494	0	0	2	1,523
2013	24	24	2,565	0	0	46	1,587
2017/18	143	42	80,511	67,859	27,080	17	5,070
2019	208	42	114,856	89,685	49,438	18	5,070
2021	322	42	273,119	176,932	129,776	31	2,964

### Record numbers

Record numbers have increased steeply since 2016, reflecting the focus point data which account for ca 65% of the total. There are 70–90,000 records each for sandflies, biting midges and mosquitoes, with > 50% (36,585/69,256) of the latter being of invasive species (*Aedes aegypti*, *Ae. albopictus*, *Aedes japonicus* and *Aedes koreicus*). However, there are fewer records (ca 37,000) and fewer species mapped for ticks than for other groups.

Table 2 also illustrates the change in polygon numbers for which data have been collected, increasing from ca 1,500 to ca 5,000 when areas surrounding Europe were included, then falling to ca 3,000 as administrative units were changed to replace small (e.g. Algeria, Albania) or large (Sweden, UK) ones to standardise their size.

### Presence or absence records

By 2013, 46% (730/1,587) of the polygons had presence or absence records but that dropped to 17% (862/5,070) as European surrounding areas (some with many small polygons) were incorporated, then increased to 31% when administrative unit size was adjusted in some countries and data collection increased (Table 2). The invasive mosquito and sandfly maps have fewer gaps (< 50%) than other groups as the absence entries are more complete, while maps of other groups are ca 20% (native mosquitoes) to 37% (biting midges) complete. These summary data hide significant interspecific variation. For example for ticks, there are very few records for the *Ornithodoros* species, but many for *Ixodes ricinus*. Many of the missing data are beyond the vectors’ environmental ranges.

### Vector maps

Distribution status maps of 42 species are currently available online. The March 2021 map for each species or species group/complex is provided in Supplementary Figures S7–45. Summary characteristics and salient features are set out next to each species map. We provide an example of the mapped outputs for each of the vector groups below.

**Figure 1:**
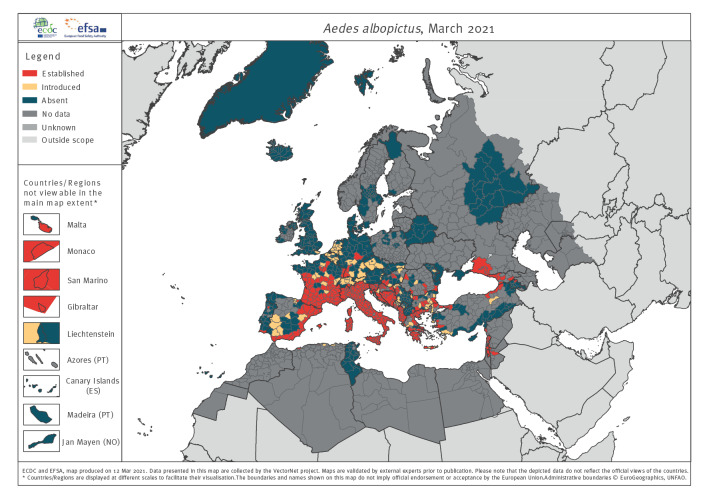
Online distribution map for representatives of the vector group mosquitos: *Aedes albopictus,* 2021

*Ae. albopictus*, the invasive tiger mosquito, a threat to human and animal health, was introduced into Albania in the 1970s and is now widespread in much of Europe. The map (Figure 1) shows numerous introduction sites, where it may become established. Widely implemented active and/or passive surveillance programmes have reported many true absence data. The VectorNet maps show more presence areas than GBIF does (e.g. France, Balkan, Caucasus). In some cases, the large polygon size (e.g. southern Russia) misrepresents much more localised distributions.

**Figure 2:**
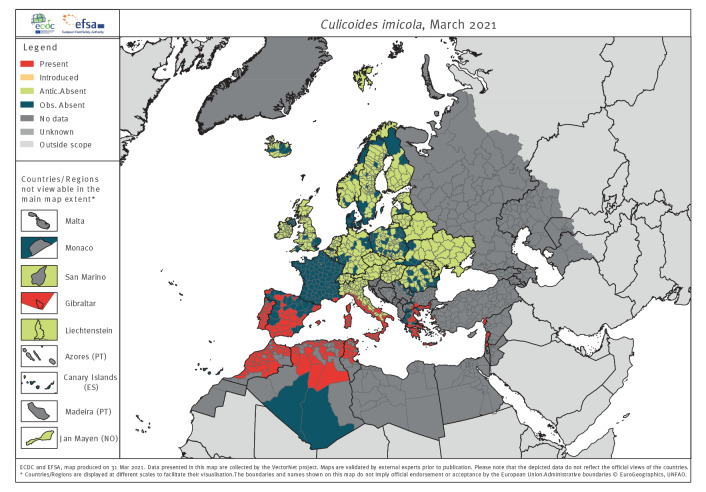
Online distribution maps for representatives of the vector group biting midges: *Culicoides imicola,* 2021

*Culicoides imicola* is a well-known vector of livestock and wild ruminant viruses such as Bluetongue virus and African horse sickness virus. *Cu. imicola* is an afrotropical species, widespread in Africa and the Middle East. The respective VectorNet map (Figure 2) shows the species to be present in the Europe around most of the Mediterranean Sea. It is abundant in the western Mediterranean islands and parts of northern Africa. It extends north to parts of North Macedonia and Bulgaria, and east to parts of Lebanon and Israel. It is also known to be widespread in Turkey, Greece and Egypt, although recent documented presence for Libya and Turkey is scarce. VectorNet has many more records for this species than either GBIF or VectorBase.

**Figure 3:**
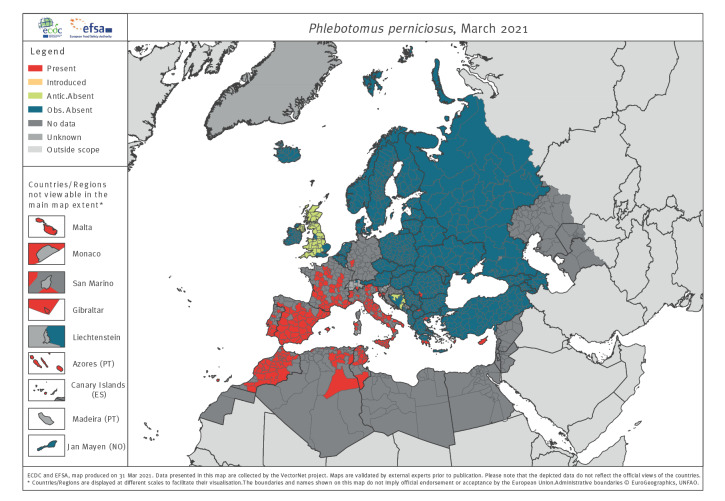
Online distribution maps for representatives of the vector group sandflies: *Phlebotomus perniciosus,* 2021

Sandflies are responsible for the transmission of *Leishmania* spp. and phleboviruses. Their distribution in the VectorNet region is very widely scattered. *Phlebotomus perniciosus* is the main vector for leishmaniasis in western Europe. Its distribution ranges from Portugal in the west to the Balkans in the east and it has been described as far north as Germany (Figure 3). In northern Africa, *P. perniciosus* has only been reported in Morocco, Algeria and Tunisia (Figure 3). Its altitudinal distribution range is wide, having been found at sea level and > 1,600 m in the Pyrenees. Maps of the species distribution in endemic countries are incomplete as surveillance is patchy. As GBIF and VectorBase records are very sparse, they do not provide similarly detailed distribution maps for Europe and its surrounding areas to those provided by VectorNet.

**Figure 4:**
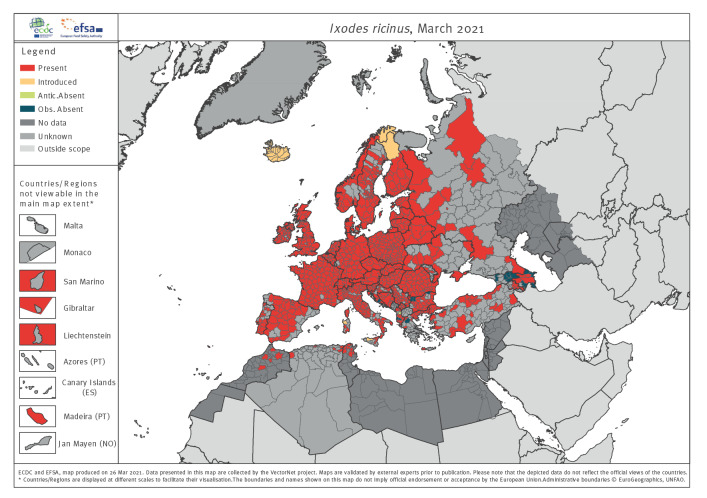
Online distribution maps for representatives of the vector group ticks: *Ixodes ricinus,* 2021

*I. ricinus* is the primary vector of Borrelia burgdorferi spp., tick-borne encephalitis virus and other pathogens such as Babesia spp., Anaplasma spp., Borrelia miyamotoi, Rickettsia spp. and Louping ill virus. Its distribution is widespread (Figure 4) but limited by low host abundance and unfavourable microclimates: southern parts of Europe are too dry and the high-altitude Alps are too cold. Due to climate change [27], ticks are increasingly found at higher altitudes. VectorNet records are biased to west, central and southern areas, with relatively few records from eastern regions, limiting the accuracy of any spatial extrapolations eastward. Some presence records in southern Europe are possibly of other similar species (Ixodes gibbosus, Ixodes inopinatus). There are records of bird mediated temporary introduction in northern regions such as Iceland, where establishment is limited by climate and an absence of hosts [28]. *I. ricinus* is one of the most recorded tick species in Europe and is also widely recorded in GBIF records but not VectorBase. Approximately 10% of GBIF records are in areas where VectorNet has no data, but these data are within the range inferred by the VectorNet presence status.

### Surveillance and distribution maps

The maps of surveillance activity per vector group are shown and described in Supplementary Figures S1–5. Data were submitted for between 68% (biting midges) and 86% (native mosquitoes) of the polygon-vector group combinations. The surveillance effort maps are patchy, rarely national in scope and vary intra-nationally for all groups. The maps highlight stark differences between vector groups: active surveillance of native mosquitoes is implemented for more months of the year than for the other vector groups and is the most diverse and widespread.

Surveillance and distribution maps are compared in Supplementary Figure S6 and by tabulating the numbers of administrative unit polygons into three categories: (i) agreeing with distribution data and recorded surveillance or agreeing with no surveillance and no distribution data; (ii) disagreeing where data are present but no surveillance reported; and (iii) disagreeing where surveillance is reported but distribution data are incomplete (Table 3). The proportion of matching polygons (i.e. recorded surveillance data, or no surveillance and no distribution data) demonstrate reasonable correspondence between surveillance and distribution reporting, especially for the flagship species like those mapped in Figures 1–4 above. Nevertheless, 40% (595/1,506) and 54% (814/1,506) of the polygons for ticks and native mosquitoes, respectively, have reported surveillance but no distribution data indicating widespread mismatches between the two activities. This is to be expected for native mosquitoes given the diversity of surveillance methods used, which may not be suitable to multi-species sampling. For sandflies, 33% (503/1,506) of the polygons have no formal surveillance reported but do have reported distribution data.

**Table 3:** Comparison of surveillance for VectorNet priority species and distribution status data at mapping polygon level, 2015–2019

Group or species	Polygons match: *with distribution data^a^ and recorded surveillance or no surveillance and no distribution data*		Polygons do not match: *distribution data present^a^, but no surveillance reported*		Polygons do not match: *no distribution data^a^, surveillance reported*	
	n	%	n	%	n	%
**Invasive mosquitoes**	1,059	70	264	18	183	12
**Native mosquitoes**	668	44	24	2	814	54
**Biting midges**	1,200	80	119	8	187	12
**Ticks**	911	60	0	0	595	40
**Sandflies**	847	56	503	33	156	10
*Aedes albopictus*	1,342	89	0	0	164	11
*Culicoides imicola*	1,433	95	0	0	73	5
*Ixodes ricinus*	1,369	91	0	0	137	9
*Phlebotomus perniciosus*	1,411	94	0	0	95	6

^a^ For vector groups (names in bold), distribution data defined as present if > 80% priority species have valid data.

### Comparison of VectorNet, Global Biodiversity Information Facility and VectorBase

Table 4 compares numbers of records and polygons covered per species (vector group) by VectorNet, GBIF and VectorBase. VectorNet distribution maps overplotted with GBIF or VectorBase points and correspondence tables are provided in Supplementary Tables S5–43. Overall, the VectorNet database has 4.8–9.3 times as many georeferenced point location records as the other databases and more records for 37 of 42 species. However, VectorNet does have substantially fewer georeferenced records for five of 42 species (vector groups): *Aedes detritus*, *An. maculipennis* complex, *Anopheles superpictus*, *Culex. pipiens* and *Ixodes persulcatus* compared to the other databases. Although for the last two, VectorNet data covers more polygons.

**Table 4:** Comparison of VectorNet, VectorBase and GBIF record numbers by VectorNet priority species and vector group, 2010-2021

Species	VectorNet not georeferenced	VectorNet georeferenced	VectorNet total records	GBIF total records	VectorBase total records	VectorNet polygons		GBIF polygons	VectorBase polygons
						With data	Present		
***Aedes aegypti***	**2,666**	**338**	**3,004**	37	686	840	24	11	6
***Ae. albopictus***	**5,334**	**8,603**	**13,937**	4,903	1,192	866	430	258	42
***Ae. atropalpus***	**2,354**	**178**	**2,532**	0	0	82	0	0	0
***Ae. caspius***	**450**	**1,369**	**1,819**	0	29	230	188	62	7
***Ae. detritus***	**96**	**77**	**173**	950	14	44	13	31	2
***Ae. japonicus***	**3,160**	**7,275**	**10,435**	176	4	851	73	29	1
***Ae. koreicus***	**2,376**	**4,295**	**6,671**	262	2	839	13	4	1
***Ae. vexans* s.l.^a^**	**1,637**	**814**	**2,451**	5,179	24	387	217	310	5
***Anopheles maculipennis* s.l.^a^**	**886**	**1,827**	**2,713**	4,030	4,085	359	332	144	476
***An. plumbeus***	**1,158**	**570**	**1,728**	0	0	387	235	56	0
***An. superpictus***	**69**	**64**	**133**	398	413	55	12	4	183
***Coquillettidia richiardii***	**76**	**715**	**791**	42	22	86	83	49	7
***Culex modestus***	**1,895**	**426**	**2,321**	386	5	440	164	23	1
***Cx. pipiens* ^a^**	**2,630**	**6,651**	**9,090**	8,020	360	552	525	210	16
***Culicoides chiopterus***	**702**	**4,170**	**4,872**	305	256	472	243	34	13
***Cu. dewulfi***	**688**	**4,075**	**4,763**	366	290	470	238	28	14
***Cu. imicola***	**2,332**	**10,041**	**12,373**	377	0	958	220	6	0
***Cu. kingi***	**994**	**4,230**	**5,224**	20	0	476	41	0	0
***Cu. newsteadi* s.l.**	**1,043**	**5,972**	**7,015**	348	0	477	402	10	0
***Cu. obsoletus* s.l./*Cu. scoticus***	**2,111**	**12,922**	**15,033**	5,272	815	563	543	112	28
***Cu. pulicaris* s.l./*Cu. lupicaris***	**1,377**	**645**	**1,034**	16	2,539	441	370	52	28
***Cu. punctatus* s.l.**	**704**	**7,620**	**8,324**	865	780	511	453	37	13
***Dermacentor reticulatus***	**1,923**	**1,175**	**3,098**	491	0	503	328	102	0
***Hyalomma lusitanicum***	**104**	**288**	**392**	53	0	0	0	3	0
***H. marginatum***	**3,542**	**1,034**	**4,576**	56	0	715	360	17	0
***Ixodes persulcatus***	**704**	**272**	**976**	743	0	83	47	16	0
***I. ricinus***	**13,218**	**6,958**	**20,176**	4,050	0	886	869	215	0
***Ornithodoros erraticus***	**5**	**6**	**11**	0	0	7	3	0	0
***Rhipicephalus sanguineus group***	**1,525**	**1,035**	**2,560**	305	0	354	354	25	0
***Phlebotomus alexandri***	**4,122**	**3,603**	**7,725**	55	1	1,116	79	0	1
***P. ariasi***	**4,222**	**2,087**	**6,309**	12	0	970	78	1	0
***P. mascittii***	**3,160**	**3,546**	**6,706**	25	10	835	99	4	1
***P. neglectus***	**3,593**	**4,707**	**8,300**	6	3,672	904	144	1	7
***P. papatasi***	**2,184**	**6,626**	**8,810**	41	1,461	842	307	2	6
***P. perfiliewi***	**2,966**	**4,630**	**7,596**	4	8	740	185	1	3
***P. perniciosus***	**3,313**	**6,886**	**10,199**	132	6	971	198	12	1
***P. sergenti***	**2,146**	**5,291**	**7,437**	1	15	842	194	1	1
***P. similis***	**4,256**	**1,481**	**5,737**	286	1,868	1,147	57	0	7
***P. tobbi***	**3,724**	**3,818**	**7,542**	8	641	999	116	25	6
*Culicoides group*	***9,951***	***57,054***	***67,005***	*10,375*	*4,680*	NA	NA	NA	NA
*Invasive mosquito group*	***15,890***	***20,689***	***36,579***	*5,378*	*1,951*	NA	NA	NA	NA
*Native mosquito group*	***8,897***	***12,847***	***21,744***	*15,373*	*4,891*	NA	NA	NA	NA
*Sandfly group*	***33,686***	***42,675***	***76,361***	*284*	*7,682*	NA	NA	NA	NA
*Tick group*	***21,021***	***10,768***	***31,789***	*5,698*	*0*	NA	NA	NA	NA
**Grand total**	**96,187**	**176,932**	**273,119**	**37,108**	**19,204**	NA	NA	NA	NA

GBIF: Global Biodiversity Information Facility; NA: not available.

^a ^Species with matching subscript letter were mapped jointly.

The geographic compatibility between the datasets is good as typically less than 5% of GBIF and VectorBase records are located in polygons that VectorNet defines as absent. However, GBIF and VectorBase have some presence records in areas where VectorNet has none, most noticeably for *Aedes vexans*, *Aedes caspius*, *Ae. detritus*, *An. maculipennis* complex, *An. superpictus*, *Coquillettidia richardii* and *Dermacentor reticulatus.* Note that these comparisons do not include VectorNet’s many thousand (> 120,000) absence records of which the other databases have very few.

### Citations in the literature

The literature search for VBORNE, VBORNET and VectorNet citations retrieved 40 citations per year from 2014 to 2017, rising to ca 60 citations per year since 2017. These figures are detailed in Supplementary Figure S46. The web statistics identified a total of 58,000 page views for the maps from 2013 to September 2021, 95% of which were for the distribution maps. Ticks and invasive mosquito maps were visited most (23,000 views each), and the most visited species in each group were *I. ricinus* (9,100), *Ae. albopictus* (11,300), *Cx. pipiens* (400) and *P. perniciosus* (1,500). For biting midges, which have been only available online since October 2020, the most accessed map has been *Cu. obsoletus/scoticus* with 63 visits. More generally, the maps appear in the first five Google search results for all groups (search terms = group name and either maps or distribution).

## Discussion

The VectorNet project series has continuously collated and integrated vector distribution data provided by a large and diverse network of volunteer professionals and academic source material, posting its maps online regularly since 2013. A wide range of formal and informal sources, rigorous validation through error trapping, iterative data entry and correction and expert opinion assures the quality of the maps made available. In addition to formal accesses and citations, which the non-exhaustive nature of the search is likely to underestimate, the scientific, public and veterinary health impact of the maps is probably most evident from their use in professional presentations, grey literature reports and project proposals. Here, the data they provide are used as reference and background information to illustrate the extent of disease risk at continental level, support investment in surveillance and control and feed media outlets. On a more technical level, the polygon and point data on which the maps are built have also underpinned a large number of spatial distribution models for many species or risk assessment of specific diseases [29-32]. Both models and maps are also useful to target regional, national and even subnational surveillance, although detailed surveillance recommendations are likely to require higher resolution than NUTS3. National normative bodies can also use the continental maps to define measures or even legal restrictions such as storage regulations for used tyres in the Netherlands that are informed by the VectorNet invasive mosquito maps. A less concrete but nevertheless valuable impact of the maps is as a ‘glue’ to encourage a large and active international entomological volunteer network to collaborate and, through an expert-led technical framework, to adopt a set of data sampling and reporting standards, enhancing compatibility of datasets across the continent.

The maps can be used by planners to justify surveillance funding and encourage reporting at subnational scale, and the spatial models derived from the point data can be used to target surveillance more precisely. Some caveats need to be mentioned. The maps are released as ‘current known distribution’, but the availability of a series of updated maps risks confusing additional or new spatial distribution data with spreading of the vector, which may not be the case. Also, as vector infection rates are often low in Europe, their presence only represents potential not active risk of disease. The move to recording point location abundance and sample effort data are not yet reflected in the published maps, partly because the body of published sampling methods are inconsistent and thus difficult to combine and partly because publications often contain only summarised data and records of high-profile species, with incomplete descriptions of sampling methodology. This last point is especially important as sampling methods are different for each vector group, and indeed for different species within each group. Improved standardisation of published data (including, for example, full sampling effort details and absence reporting, as illustrated in Aedes Invasive Mosquito Cost Action (AIMCOST) continental survey outputs [33]) will enable the acquisition of more consistent quantitative distribution data and so the production of density as well as presence/absence maps.

A key feature of the recent phases of the VectorNet projects is a focus on acquiring and maintaining standardised data records, including specimen numbers, sampling dates and locations, sampling effort and methodologies and validated species identifications. This provides the potential for detailed analysis of changes in distribution and abundance and a wide range of other spatial analyses.

## Conclusions

The VectorNet datasets compare favourably with other datasets in terms of coverage and record numbers because they record absences and are validated by experts. They are, however, less accessible in terms of interactive querying and download. The datasets and many maps are (still) patchy and incomplete, mostly because of missing absence data in areas outside known vector ranges, or because absence is not recorded when suitable trapping efforts have resulted in zero records.

Planned improvements, such as producing density and seasonality maps and including inferred absences, would not only make the maps more informative but would also facilitate tracking expansion and change more effectively. Such changes would enhance what is already a valuable dataset providing validated vector distribution information to a wide range of professional and lay audiences.

## Supplementary Data

4509000 Supplementary Material
