# Supplementary material for: VectorNet: collaborative mapping of arthropod disease vectors in Europe and surrounding areas since 2010
Source: Euro Surveill. 2023 Jun 29;28(26):2200666. doi: 10.2807/1560-7917.ES.2023.28.26.2200666 (PMC10311950; doi:10.2807/1560-7917.ES.2023.28.26.2200666)

VectorNet: collaborative mapping of arthropod disease vectors in Europe and surrounding areas since 2010

SUPPLEMENTARY INFORMATION

Section I: Contributors

Section II: Literature Search Protocols

Section III: Data Entry Worksheet and Column Descriptions

Section IV: Quality Indicators

Section V: Maps

Section VI: Citations

This supplementary material is hosted by *Eurosurveillance* as supporting information alongside the article "*VectorNet: collaborative mapping of arthropod disease vectors in Europe and surrounding areas since 2010*", on behalf of the authors, who remain responsible for the accuracy and appropriateness of the content. The same standards for ethics, copyright, attributions and permissions as for the article apply. Supplements are not edited by *Eurosurveillance* and the journal is not responsible for the maintenance of any links or email addresses provided therein."

SUPPLEMENTARY INFORMANTION SECTION I: Supplementary Table S1: Contributor List

Contributors involved in all constituent projects. Key for Vector Group Column: C= Culicoides, M = Mosquitoes, S= Sand flies, T=Ticks, VEN = VectorNet Entomological Network Member, VGL = Vector Group Leader.

Contributor	Country	Institution	Vector Group
Akiner M	Turkey	Recep Tayyip Erdogan University, Faculty of Arts and Sciences, Department of Biology, Fener, Rize	M
Albieri A	Italy	Centro Agricoltura Ambiente, “Giorgio Nicoli”, Crevalcore	M
Alfreðsson M.	Iceland	Icelandic Institute of Natural History	T, VEN
Alishani M.	Kosovo	University of Prishtina	T
Alkan F.	Turkey	Ankara University	C
Alten B.	Turkey	Hacettepe University, Ankara	C, S, M, VGL, VEN
Alves M.	Portugal	Centro de Estudos de Vectores e Doenças Infecciosas Dr. Francisco Cambournac, Águas de Moura	M
Aranda C.	Spain	Consell Comarcal del Baix Llobregat	M
Arnthorsdottir AL.	Iceland	Icelandic Food And Veterinary Authority (MAST)	C
Bakran-Lebl K.	Austria	AGES - Österreichische Agentur für Gesundheit und Ernährungssicherheit GmbH, Wien	M
Balenghien T.	France	CIRAD	C, VGL, VEN
Barceló C.	Spain	University of the Balearic Islands	C
Baylis M.	UK	University of Liverpool	C
Baymak D.	Kosovo		T
Beck R.	Croatia	University of Zagreb	T
Berrell M.	UK	UK Health Security Agency	T
Berriatua E.	Spain	Universidad de Murcia, Spain	VGL, VEN
Bisia M.	Greece	Veterinary Centre of Athens	C
Blackwell A.	Scotland	APS Biocontrol Ltd	C
Bødker R.	Denmark	University of Copenhagen	C, VEN
Bouattour A.	Tunisia	Universite´ de Tunis El Manar, Institut Pasteur de Tunis	VEN
Bourquia M.	Morocco	Institut Agronomique et Vétérinaire Hassan II	C
Boussaa S.	Morocco	ISPITS, Ministère de Santé et Protection sociale, Rabat	S
Boutsini S.	Greece	Ministry of Rural Development and Food	C, S, VEN

Contributor	Country	Institution	Vector Group
Brugger K.	Austria	University of Veterinary Medicine, Vienna	C, VEN
Brzonova. J.	Czech Republic	Charles University Prague, Czechia	C
Bueno Mari R.	Spain	Lokimica, Valencia	M
Calzolari M.	Italy	ISZLER, Reggio Emilia	M
Carpenter S.	UK	The Pirbright Institute	C
Chakhunashvili, D.	Georgia	National Center for Disease Control and Public Health	VEN
Clausen CG.	Denmark	Technical University of Denmark	C
Colenutt C.	UK	The Pirbright Institute	C
Collantes F.	Spain	Facultad de Biología , Universidad de Murcia	M, S
Culverwell C.L.	Finland	University of Helsinki	C, M, VEN
Cvetkovikj A.	N. Macedonia	Faculty of veterinary medicine	C
Dağalp SB.	Turkey	Ankara University	C
Dahle S.	Norway	Norwegian Institute for Nature Research	C, VEN
Dascălu L.	Romania	Institute for Diagnosis and Animal Health	C
De Regge N.	Belgium	Sciensano	C
Deblauwe, I	Belgium	Institute of Tropical Medicine, Antwerp, Belgium	C, T, M, VEN
Delacour Estrella S.	Spain	Instituto Agroalimentario de Aragón, Facultad de Veterinaria. Universidad de Zaragoza	M
Dikolli Velo E.	Albania	Institute of Public Health, Tirana	S, T, M, VEN
Di Luca M.	Italy	Istituto Superiore di Sanità, Roma	M
Duscher G.	Austria	University of Veterinary Medicine Vienna	T
Dvořák V.	Czechia	Charles University Prague, Czechia	S, VEN
Elbers A.	Netherlands	Wageningen Bioveterinary Research	C
England M.	United-Kingdom	The Pirbright Institute	C
Eritja R.	Spain	CREAF, University of Barcelona	M
Estrada-Peña A.	Spain	University of Zaragoza	T
Eydal M.	Iceland	University of Iceland	T

Contributor	Country	Institution	Vector Group
Falcuta E.	Romania	”Cantacuzino” Military-Medical National Institute for Research and Development. Bucharest	S, M, VEN
Farkas, R.	Hungary	Szent Istvan University	VEN
Filatov S.	Ukraine	Institute of Experimental and Clinical Veterinary Medicine	C, VEN
Foxi C.	Italy	Università degli Studi di Sassari	C
Führer H.-P.	Austria	University of Veterinary Medicine, Vienna	M
Gandy S.	UK	UK Health Security Agency	T
Garcia Vozmediano A.	Italy	University of Turin	T
Gardès L.	France	Cirad	C
Garros C.	France	Cirad	C
Geller J.	Estonia	National Institute of Health Development	T
Gewehr S.	Greece	Ecodevelopment S.A.	M
Gilbert L.	UK	University of Glasgow	T
Gillingham E.	UK	UK Health Security Agency	T
Goffredo M.	Italy	Istituto Zooprofilattico Sperimentale dell'Abruzzo e del Molise "G. Caporale"	C, VEN
Golovljova I.	Estonia	National Institute of Health Development, Estonia	T
Gospodinovic I.	Serbia	University of Novi Sad	T
Gunay F.	Turkey	Hacettepe University, Ankara	S, M
Haddad N.	Lebanon	Faculty of Public Health, Lebanese University, Fanar	C, M, VEN
Hansen J.	Faroe Is	Tjóðsavnið - Faroe Islands National Museum	T
Hansen MF.	Denmark	University of Copenhagen	C
Hansford K.	UK	UK Health Security Agency	T
Harrat, Z.	Algeria	Laboratoire éco-épidémiologie Parasitaire et Génétique des Populations, Institut Pasteur d’Algérie	VEN
Hendrickx G.	Belgium	AviaGIS, Belgium	S
Henney J.	Guernsey	Environment Guernsey	T
Hlavackova K.	Czechia	Charles University Prague, Czechia	S
Hodzik A.	Austria	University of Veterinary Medicine Vienna	T
Høye TT.	Denmark	Department of Bioscience and Arctic Research Centre	C
Hristescu D.	Romania	Institute for Diagnosis and Animal Health	C
Hubalek Z.	Czech Republic	The Czech Academy of Sciences	T
Huber K.	France	INRAe	C
Hufnagl P.	Austria	Austrian Agency for Health and Food Safety, Institute for Medical Microbiology and Hygiene	M
Hvidsten D.	Norway	Nordlandssykehuset HF	T
Ibanez Justicia A.	Netherlands	Centre for Monitoring of Vectors NVWA	M
Ivanovic I.	Serbia	University of Novi Sad	T
Ivovic V.	Slovenia	University of Primorska, Koper, Slovenia	S, VEN
Jaarma, K.	Estonia	Animal Health and Welfare Department, Estonia	VEN
Jääskeläinen A.	Finland	Independent	T
Jaenson T.	Sweden	University of Uppsala	T
Janev-Holker, N.	Croatia	Croatian Institute of Public Health	VEN
Johnston C.	UK	UK Health Security Agency	T
Jöst A.	Germany	Kommunale Aktionsgemeinschaft zur Bekämpfung der Schnakenplage, Speyer	M
Jourdain, F.	France	Santé publique France	VEN
Jurisic A.	Serbia	University of Novi Sad	T
Kalan K.	Slovenia	University of Primorska	M
Kapo N.	Bosnia and Herzegovina	University of Sarajevo	T
Kampen H.	Germany	Friedrich-Loeffler-Institut, Greifswald - Insel Riems	M, VEN
Kanani K.	Jordan	Parasitic and Zoonotic Diseases Department, Vector-Borne Diseases programmes manager, MOH, Ramallah, Jordan,	VEN
Karakus M.	Turkey	Hacettepe University, Ankara	S
Kasap O.	Turkey	Hacettepe University, Ankara	S
Kavur H.	Turkey	Cukurova University, Dept of Medical Parasitology, Adana	S, M
Kenyeres Z.	Hungary	Acrida Conservational Research L.P., Tapolca	M
Khalin A:	Russia	Zoological Institute, Russian Academy of Sciences, St. Petersburg	M
Khallaayoune K.	Morocco	Institut Agronomique et Vétérinaire Hassan II	C
Klobucar A.	Croatia	Andrija Stampar Teaching Institute of Public Health, Zagreb	M
Kocišová A.	Slovakia	University of Veterinary Medicine and Pharmacy	C, VEN

Contributor	Country	Institution	Vector Group
Krüger A.	Germany	Bernhard Nocht Institute for Tropical Medicine, Hamburg	M
Kuhn C.	Germany	Umweltbundesamt, Berlin	M
L'Ambert G.	France	EID Méditerranée, Montpellier	M
Larska M.	Poland	National Veterinary Research Institute	C
Lindstrom A.	Sweden	National Veterinary Research Institute	VEN
Lucientes J.	Spain	Facultad de Veterinaria. University of Zaragoza	C
Madeira S.	Portugal	CIISA - Centro de Investigação Interdisciplinar em Sanidade Animal	C
Mammadova K.	Azerbaijan	Azerbaijan State Agricultural University	T, M, VEN
Mancini G.	Italy	Istituto Zooprofilattico Sperimentale dell'Abruzzo e del Molise 'G. Caporale'	C
Martínez-Barciela Y.	Spain	Rede Galega de Vixilancia de Vectores (ReGaViVec), Xunta de Galicia, Department of Ecology and Animal Biology, Faculty of Biology, University of Vigo	M
Mathieu B.	France	Institut de Parasitologie et de Pathologie Tropicale, Strasbourg	C
McGinley L.	UK	UK Health Security Agency	T
Meadows S.	Jersey	States of Jersey Department of Environment	T
Medlock J.	UK	UK Health Security Agency	M, T , VGL, VEN
Michaelakis A.	Greece	Benaki Phytopathological Institute, Arhens	M
Melashvili G.	Georgia	Agricultural University of Georgia	T
Mihalca A. D.	Romania	University of Agricultural Sciences and Veterinary Medicine of Cluj-Napoca	S, T, VGL, VEN
Mikov O.	Bulgaria	National Centre of Infectious and Parasitic Diseases, Sofia	M, S, T, VEN
Miranda MA.	Spain	University of the Balearic Islands (UIB)	C, M, VEN
Misfud, D.	Malta	Universtity of Malta	VEN
Montalvo T.	Spain	CIBER Epidemiología y Salud Pública (CIBERESP), Madrid	M
Montarsi, F	Italy	Istituto Zooprofilattico Sperimentale delle Venezie (IZSVe), Legnaro	M
Mosca A.	Italy	Istituto per le Piante da Legno e l'Ambiente, Turin	M
Muja-Bajraktari N.	Kosovo	Faculty of Mathematics and Natural Sciences, University of Prishtina	M

Contributor	Country	Institution	Vector Group
Müller P.	Switzerland	Swiss Tropical and Public Health Institute, Basel	M
Muñoz C.	Spain	University of Murcia	S
Murchie A.	United-Kingdom	Agri-Food and Biosciences Institute	C
Muscat I.	Jersey	Jersey General Hospital's Pathology Laboratory	T
Nebogatkin I.	Ukraine	National Academy of Sciences of Ukraine	T
Nițescu C.	Romania	Institute for Diagnosis and Animal Health	C
Oguz G.	Turkey	Hacettepe University, Ankara	C, S
Olafsson E.	Iceland	Icelandic Institute of Natural History	T
Omeragic J.	Bosnia and Herzegovina	University of Sarajevo - Veterinary Faculty	T, VEN
Ondráková M.	Czechia	Charles University Prague, Czechia	C
Orłowska A.	Poland	National Veterinary Research Institute	C
Orshan, L.	Israel	Ministry of Health	VEN
Osmani A.	Kosovo		T
Osório, H.	Portugal	National Institute of Health/ Center for Vectors and Infectious Diseases Research	VEN
Ozbel Y.	Turkey	Ege University Faculty of Medicine Department of Parasitology, Izmir	S
Ozoliņa Z.	Latvia	Institute of Food safety, Animal Health and Environment 'BIOR'	C, VEN
Pajovic I.	Montenegro	University of Montenegro, Biotechnical Faculty	S, C, M, VEN
Papa A.	Greece	Aristotle University of Thessaloniki	T
Paronyan L.	Armenia	Zoonotic and Parasitic Diseases Epidemiology Department, National Center for Disease Control and Prevention, Ministry of Health	T, VEN
Pereira da Fonseca I.	Portugal	CIISA - Centro de Investigação Interdisciplinar em Sanidade Animal	C
Perrin Y.	France	EID Méditerranée, Montpellier	M
Pesko J.	Czech Republic	The Czech Academy of Sciences	T
Petric D.	Serbia	Faculty of Agriculture, University of Novi Sad	C, S, T, M, VEN
Petrovic A.	Serbia	University of Novi Sad	T
Plenge-Bönig A.	Germany	Institute for Hygiene and Environment, Hamburg	M
Prioteasa F-L.	Romania	"Cantacuzino" Military-Medical National Institute for Research and Development.Bucharest, Bucharest	M

Contributor	Country	Institution	Vector Group
Pudar D.	Serbia	University of Novi Sad	C
Rakotoarivony I.	France	Cirad, Montpellier	C
Ramilo D.	Portugal	CIISA - Centro de Investigação Interdisciplinar em Sanidade Animal	C
Rettich F.	Czech Republic	Státní Zdravotní Ústav, Praha	M
Risueño J.	Spain	University of Murcia	S
Robert V.	France	Mivegec laboratory, University of Montpellier, IRD, CNRS, Montpellier	M
Rodze I.	Latvia	Institute of Food safety, Animal Health and Environment 'BIOR'	C
Rozek W.	Poland	National Veterinary Research Institute	C
Rudolf I.	Czech Republic	The Czech Academy of Sciences	T
Ruiu L.	Italy	Università degli Studi di Sassari	C
Samy, A.	Egypt	Entomology Department, Faculty of Science, Ain Shams University	VEN
Santilli A.	Italy	Istituto Zooprofilattico Sperimentale dell'Abruzzo e del Molise 'G. Caporale'	C
Saska A.	Slovenia	Science and Research Center,Koper	S
Sawalha S.	Palestine	Ministry of Health	VEN
Schaffner F.	France	Francis Schaffner Consultancy	M, VGL
Seglina Z.	Latvia	Institute of Food safety, Animal Health and Environment 'BIOR'	C
Serafin I.	Spain	Instituto Universitario de Enfermedas Tropicales y Salud Publica de Canarias	M
Severini F.	Italy	Istituto Superiore di Sanità, Roma	M
Sghaier S.	Tunisia	Institut de la Recherche Vétérinaire de Tunisie	C
Shaibi T.	Libya	Reference Laboratory of Parasites & Vector Borne Diseases, NCDC Libya, and Zoology Department, Faculty of Science, University of Tripoli, Libya,	VEN
Sherifi K.	Kosovo	University of Hasan Prishtina	T, VEN
Simonsen W.	Faroe Is	National Hospital of the Faroe Islands	T
Smreczak M.	Poland	National Veterinary Research Institute	C
Sohier C.	Belgium	CODA-CERVA	C
Sokolovska N,	Macedonia	Center for public health-Skopje	M
Soltész Z.	Hungary	Centre for Ecological Research, Eötvös Lóránd Research Network, Vácrátót	M
Sousa C. A.	Portugal	Instituto de Higiene e Mdecicina Tropical, Lisbon	S, M

Contributor	Country	Institution	Vector Group
Stachurski F.	France	CIRAD, Montpelier	T
Stefanovska J.	N. Macedonia	Faculty of Veterinary Medicine	C, VEN
Stougiou D.	Greece	Veterinary Centre of Athens	C
Stroo A.	Netherlands	Centre for Monitoring of Vectors NVWA	VEN
Stuen S.	Norway	Norwegian University of Life Sciences	T
Sulesco T.	Moldova	Institute of Zoology, Chisinau	VEN
Sztikler J.	Hungary	National Public Health Center,Department of Communicable Disease Epidemiology	M
Tachfine H.	Morocco	Direction de l'épidémiologie et de lutte contre les maladies	VEN
Tchakarova S.	Bulgaria	National Diagnostic and Research Veterinary Medical Institute	C
Toma L.	Italy	Istituto Superiore di Sanità, Roma	M
Tomassone L.	Italy	Università di Torino	T
Van Bortel	Belgium	Institute of Tropical Medicine, Antwerp, Belgium	M
Vatansever Z	Turkey	Kafkas Univetsity, Kars	T
Vaux A.	UK	UK Health Security Agency	M
Verdun Castello M.	Spain	CReSA-IRTA, Universitat Autònoma de Barcelona	C
Veronesi E.	Switzerland	National Centre for Vector Entomology, Zurich	C
Vial L.	France	CIRAD, Montpellier	T
Votýpka J.	Czech Republic	Charles University, Praha	S
Walder G.	Austria	Medical University of Innsbruck	M
Walochnik, J.	Austria	Medical University Wien	VEN
Weigand A.	Luxembourg	National Museum of Natural History	T, VEN
Wieckowski F.	Alderney	Alderney Wildlife Trust	T
Zamburlini R.	Italy	University of Udine	M
Zhioua E.	Tunisia	Insitut Pasteur de Tunis	T
Zintl A.	Ireland	University College, Dublin	T, VEN
Zygutiene M.	Lithuania	Centre for Communicable diseases and AIDS, Vilnius	S, M

SUPPLEMENTARY INFORMATION SECTION II: Supplementary Table S2: Literature Search Protocols

Databases: Medline/Pubmed; Scopus; Embase; Google Scholar; Web of Science (if available to Institution)

More specialist institutional databases may also be used such as CORDIS, as well as, dependent on institutional access. Other published sources include: Online accessible dedicated web-sites such as those maintained by national public and veterinary health institutes and services (examples: https://solidarites-sante.gouv.fr/sante-et-environnement/risques-microbiologiques-physiques-et-chimiques/especes-nuisibles-et-parasites/article/cartes-de-presence-du-moustique-tigre-aedes-albopictus-en-france-metropolitaine; https://www.nvwa.nl/onderwerpen/muggen-knutten-en-teken) are checked continually for primary data sources.

Time limits

Default time limits: 2016–present

Vector group specific

Mosquitoes: 2013–present for RVF Mandate: *caspius*, *detritus*, *pipiens*, *albopictus*, *japonicus*, *vexans*, *theileri*; 1980–present for species not extracted before: *claviger*, *richiardii*, *annulata*, *coluzzii*; 2016–present for all others

Culicoides: 1980–present for countries not already covered during VectorNet 2014/18 (Austria, Croatia, Czech Republic, Denmark, Germany, Ireland, Norway, Slovakia and Sweden); 2016–present for all others

Ticks: All years for *Hyalomma lusitanicum*; 2016–present for all others

Sand flies: All years for *langeroni*, *alexandri*, *kandelaki*, *major*; 2016–present for all others

Minimum data requirements for eligibility

Data Source: a personal communication without an electronic document with the relevant data is not sufficient, but an unpublished report is fine as long as it is available for access.

Location: data with a precision to less than NUTS2 level or equivalent should not be considered, data with NUTS2 precision should be marked as such.

Vector species: data where samples are not identified to the level of species or species groups, except where currently allowed in the migrated VectorNet list, should not be considered: -Exclusion if identification is not based on reliable method within species complexes (e.g. *Anopheles maculipennis* complex: morphology accepted only for *An. sacharovi* adults, molecular methods only for all other cases).

Reported status or number of specimens caught: if the number of specimens caught is not reported, the reported status is a mandatory minimum data requirement.

Collection effort end date: If the source does not have a valid vector collection end date with accuracy less than a year, the data should not be considered.

Search Terms: The syntax of the search strings with the terms listed below needs to be built and adjusted dependent on the database. The terms and search strategy may be adjusted depending on the quality of the search results. The terms are listed in four lists: a 'datatype OR list', a 'Country/Region OR list', a 'Genus OR list' and a 'Species OR list'. The latter two are vector group-specific lists. In the searches, these are combined into search-strings according to the following principle:

Applicable to all Vector groups

Terms in Title OR in Abstract: 'Genus OR list' AND in all fields: 'Species OR list', AND in all fields: 'Datatype OR list' AND in all fields: 'Country/Region OR list'

*Datatype OR List***:** distribution OR presence OR occurrence OR report* OR spread OR dispers* OR detect* OR abundan* OR densit* OR number OR absen* OR surv* OR monitor* OR introduc* OR intercept*

Country/Region OR list: Europe* OR EU OR "Mediterranean Basin" OR "Mediterranean area" OR Balkan* OR Scandinavia* OR "Iberian peninsula" OR Aland OR Albania* OR Andorra* OR Atlantic OR Austria* OR Belgi* OR "Black sea" OR Bosnia* OR Herzegovina OR Bulgaria* OR Croatia* OR Cypr* OR "Czech Republic" OR Denmark OR Greenland OR German* OR Spain OR Estonia* OR Finland OR "Faroe islands" OR France OR Corsica* OR Greece OR

Gibraltar OR Hungary OR Iceland* OR Ireland OR Italy OR Sicil* OR Sardinia* OR Kosov* OR Latvia* OR Liechtenstein OR Lithuania* OR Luxembourg OR Macedonia* OR FYROM OR Malta OR Monac* OR Monegasqu* OR Montenegr* OR Netherlands OR Norway OR Poland OR Portug* OR Slovenia* OR Romania* OR "San Marino" OR Serbia* OR Slovakia* OR Switzerland OR Sweden OR "United Kingdom" OR "British Isles" OR "Great Britain" OR Wales OR England OR Scotland OR Turk* OR "Vatican city" OR Svalbard OR Israel* OR Palestin* OR Jordan* OR Leban* OR Syria* OR Morocc* OR Algeria* OR Tunisia* OR Libya* OR Egypt* OR "Western Sahara*" OR Armenia* OR Azerba* OR Belarus OR Bielorussia* OR Georgia* OR Moldov* OR Russia* OR Yugoslavia* OR Ukrain* OR Ukrayin* OR OR USSR OR SSSR OR "Soviet Union" OR British OR Irish OR Scottish OR Welsh OR "Channel Islands" OR Jersey OR Guernsey OR Sark OR French OR OR Greek OR Italian OR Spanish OR Swiss OR Swedish OR Transcaucasia* OR CaucasusOR Danish OR Finnish OR Norwegian OR Baltic OR Czech* OR Hungarian OR Polish OR Mediterranean OR Sahara OR OR Majorca* OR Mallorca* OR Minorca* OR Ibiza OR Azores OR Canar* OR Balearic* OR "Member St*" OR "North Africa*" OR Kazakh*OR Uzbek* OR Karakalpakstan* OR Crimea* OR Nakhchivan* OR Alsac* OR Abkhazia* OR Adjara* OR Ossetia OR Athos OR Aosta* Friuli* Giulia* Trentin* Adige Südtirol* OR "South Tyrol*" Găgăuzia* Transnistria* Madeira* Catalan Catalonia* Adygea* Bashkortostan* Chechnya* Chuvashia* Dagestan* Ingushetia* Karbardin* Kalmykia* Karachay Komi* Karelia "Mari El"* Mordovia* Tatarstan* Udmurtia* Nenets Vojvodin* Metohija* Srpska* Andalusia* Aragon* Asturias* "Balearic Islands" Basque Cantabria* Castile* Mancha* León Extremadura* Galicia* Rioja* Madrid Murcia* Navarr* Valencia* Ceuta* Melilla* "Isle of Man"

Terms applicable to individual Vector groups

Genus OR list:

Mosquitoes: *Aedes* OR *Stegomyia* OR *Ochlerotatus* OR *Anopheles* OR *Culex* OR mosquito* OR *Culicidae* OR *Culiseta* OR *Coquillettidia*

Culicoides: *Culicoides* OR "biting midge*"

Ticks: *Ixodes* OR *Dermacentor* OR *Rhipicephalus* OR *Hyalomma* or OR *Ornithodoros* OR "ticks" OR "soft ticks" OR "hard ticks"

Sand flies: *Phlebotomus* OR *Leishmania* OR sand flies OR "sand flies" OR "sand fly"

Species OR list:

Mosquitoes: *albopict** OR *aegypti* OR *atropalpus* OR *koreicus* OR *japonic** OR *caspius* OR *coluzzii* OR *detritus* OR *vexans* OR *plumbeus* OR *maculipennis* OR *messeae* OR *atroparvus* OR *sacharovi* OR *labranchiae* OR *superpictus* OR *modestus* OR *perexiguus* OR *univittatus* OR *pipiens* OR *molestus* OR *quinquefasciatus* OR *annulata* OR *claviger* OR *antennatus* OR *richiardii* OR *theileri* OR *torrentium* OR *tritaeniorhynchus*

Culicoides: N/A

Ticks: ricinus OR persulcatus OR reticulatus OR sanguineus OR marginatum OR erraticus OR lusitanicum

Sand flies: *alexandri* OR *ariasi* OR *kandelakii* OR *major* OR *mascitii* OR neglectus OR *papatasi* OR *perniciosus* OR *perfiliewi* OR *tobbi* OR *sergenti* OR *similis* OR *langeroni*

SUPPLEMENTRAY INFORMATION SECTION III: Supplementary Table S3: Data Entry Worksheet and Column Descriptions

Key and Notes:

Bold =Worksheet Title; *Italic = Calculated Field*.

If Field Value selected with dropdown lists, column Validation Lookup sheet indicates “Lists”. List definitions in Worksheet Lists

**Worksheet**	**Column Name**	**Column Description**	**Validation Lookup sheet**	**Validation lookup sheet column**	**Permitted range**
Data Entry		**Main Data entry sheet**			
**DataEntry**	SourceID	Unique ID, assigned on addition to dataset	None		
**DataEntry**	VectorCategory	*Vector Group: Ticks, Culicoides midges, Sand flies, Invasive Mosquitoes, Native Mosquitoes.*	*Lists*	*B*	*In Lookup*
**DataEntry**	VectorSpeciesName	*Vector Name*	*Lists*	*B and D,F,H,J*	*In Lookup*
**DataEntry**	Country	*Country Code, validated against list in worksheet LocationCodes/Country*	*LocationCodesCountry*		*In Lookup*
**DataEntry**	LocationCode	*Administrative Unit Code, validated against list in worksheet LocationCodes/Counry*	*LocationCodesCountry*		*In Lookup*
**DataEntry**	*LocationName*	*Administrative Unit Name, calculated from previous column using worksheet CodesVLOOKUP*	*Codes VLOOKUP*		*In Lookup*
**DataEntry**	Longitude E/W	Longitude East or West (E.W)	Lists	AT	*In Lookup*
**DataEntry**	Longitude (degrees)	Longitude degrees	None		
**DataEntry**	Longitude (minutes)	Longitude minutes	None		
**DataEntry**	Longitude (seconds)	Longitude seconds	None		
**DataEntry**	*Longitude (decimal degrees) (X)*	*Calculated Latitude in decimal degrees calculated from previous columns*	None		
**DataEntry**	Latitude N/S	Latitude East or West (E.W)	Lists	AV	*In Lookup*
**DataEntry**	Latitude (degrees)	Latitude degrees	None		
**DataEntry**	Latitude (minutes)	Latitude minutes	None		
**DataEntry**	Latitude (seconds)	Latitude seconds	None		
**DataEntry**	*Latitude (decimal degrees) (Y)*	*Calculated Latitude in decimal degrees calculated from previous columns*	None		
**DataEntry**	User Entry Longitude (X)(decimal degrees)	User entry Longitude in decimal degrees	None		

Worksheet	Column Name	Column Description	Validation Lookup sheet	Validation lookup sheet column	Permitted range
**DataEntry**	User Entry Latitude (Y) (decimal degrees)	User entry Latitude in decimal degrees	None		
**DataEntry**	PrecisionLocation	Point type or polygon	Lists	L	*In Lookup*
**DataEntry**	VectorLifeStage	Vector life stages: adult, nymph, pupa, eggs, Unknown	Lists	AL	*In Lookup*
**DataEntry**	VectorSex	Vector sex	Lists	AH	*In Lookup*
**DataEntry**	NumberOfVectorsCaught	Number of vectors caught:	None		
**DataEntry**	CollectionPlaceID	Collection place	None		
**DataEntry**	CollectionEffortStartDate	Start date for collection effort	None		
**DataEntry**	CollectionEffortEndDate	End date for collection effort	None		
**DataEntry**	VectorCollectionMethod	Collection method used (permitted methods customised for vector type)	Lists	B: AL: N, P,R, T, V, X	*In Lookup*
**DataEntry**	CollectionEffortP1Value	Collection effort as number of units in following column	None		
**DataEntry**	*UnitsEffortP1*	*Calculated from preceding columns using worksheet VLookupTableUnitsVectorCollect*	VLookupTableUnitsVectorCollect		*In Lookup*
**DataEntry**	CollectionEffortP2Value	Collection effort as number of units in following column	None		
**DataEntry**	*UnitsEffortP2*	*Calculated from preceding columns using worksheet VLookupTableUnitsVectorCollect*	VLookupTableUnitsVectorCollect		*In Lookup*
**DataEntry**	VectorHostSpecies	Vector Host Species	Lists	AP	*In Lookup*
**DataEntry**	Host Bodypart	Host Bodypart not validated	None		
**DataEntry**	VectorIdentificationMethod	Method used to identify vector species	Lists	AB	*In Lookup*
**DataEntry**	ShelteredEnvironment	Whether sample site was sheltered or not	Lists	AR	*In Lookup*
**DataEntry**	ReportedDistributionStatus	Reported distribution status: Present, Absent, Anticipated Absent, Introduced, Unknown, No Data	Lists	B, Z	*In Lookup*
**DataEntry**	PathogenName	Name of Pathogen for which test carried out	Lists	AD	*In Lookup*
**DataEntry**	PathogenDetectionMethod	Method used to detect pathogen	Lists	AF	*In Lookup*
**DataEntry**	NumberOfVectorTested	Number of vectors tested	None		

Worksheet	Column Name	Column Description	Validation Lookup sheet	Validation lookup sheet column	Permitted range
**DataEntry**	PositivePathogenDetection	Whether pathogen detected or not	None		
**DataEntry**	PathogenComment	Additional information about pathogen	None		
**DataEntry**	SourceType	Type of information source	None		
**DataEntry**	PublicationTitle	Publication Title	None		
**DataEntry**	Author	Publication Author	None		
**DataEntry**	YearOfPublication	Publication Year	None		
**DataEntry**	DOI	Publication DOI	None		
**DataEntry**	VectorNetFieldStudyID	Vectornet field study ID if applicable	None		
**DataEntry**	URL	Reference URL	None		
**DataEntry**	NotesFromDataExpert	Option Notes from Validator	None		
**DataEntry**	Data expert name	Data expert: name and family name	None		
**DataEntry**	*CollectionEffortStartDate_ddmmyyyy*	*Calculated standardised date for start of Collection effort from CollectionEffortStartDate (x)*	None		
**DataEntry**	*CollectionEffortEndDate_ddmmyyyy*	*Calculated standardised date for start of Collection effort from CollectionEffortEndDate (x)*	None		
**DataEntry**	Submitter email (first row only)	email of data submitter	None		
**Name2Geocode**		**Worksheet with Tool to identify Location code from Name**			
**Name2Geocode**	LOCATION NAME	User entered Location Name			
**Name2Geocode**	*LOCATION CODE*	*Location code returned by Tool*			
**Name2Geocode**	*LOCATION TYPE*	*Location Administrative Unit type returned by Tool*			
**Lists**		**Look Up tables as used in Column: Validation lookup sheet column**			
**Lists**	VectorCategoryCode	Look Up for Vector category Code			
**Lists**	Mosquito	Look Up for mosquito species names			
**Lists**	Sandfly	Look Up for sandfly species names			
**Lists**	Culicoides	Look Up for Culicoides species names			

Worksheet	Column Name	Column Description	Validation Lookup sheet	Validation lookup sheet column	Permitted range
**Lists**	Tick	Look Up for Tick species names			
**Lists**	PrecisionCoordinate	Look Up for precision of coordinates entered			
**Lists**	TrapTypeName	Look Up for trap type			
**Lists**	VectorCollectionMethodName	Look Up for collection method			
**Lists**	CollectionMosquitoAdults	Look Up for collection method of Adult Mosquitoes			
**Lists**	CollectionMosquitoLarvae	Look Up for collection method of Larval Mosquitoes			
**Lists**	CollectionMosquitoNymph_Pupa	Look Up for collection method of Nymphal or Pupal Mosquitoes			
**Lists**	CollectionMosquitoEggs	Look Up for collection method of Mosquito Eggs			
**Lists**	VectorDistributionStatusInvasive	Look up for Vector Status Invasive or Native			
**Lists**	VectorIdentificationMethod	Look up for Vector Identification Method			
**Lists**	PathogenNameMosquito	Look up for Pathogens transmitted by Mosquitoes			
**Lists**	PathogenDetectionMethod	Look up for Pathogen Detection Methods			
**Lists**	VectorSex	Look Up Vector Sex			
**Lists**	VectorLifeStage	Look Up for Vector Life Stage			
**Lists**	VectorInformationSourceType	Look Up Type of Vector Information Source			
**Lists**	VectorHostSpeciesName	Look Up Vector Host Species Name			
**Lists**	ShelteredEnvironment				
**Lists**	Longitude E/W	Look Up for Longitude Abbreviations			
**Lists**	Latitude N/S	Look Up for Latitude Abbreviations			
**LocationCodesby Country**			**Look Up for Location Codes and Names: Columns in pairs, first as Admin Unit Code, second as Admin Unit Names by Country**		
**CodesVLOOKUP**		**Look Up for Location codes and Names, formatted for VLOOKUP function**			

Worksheet	Column Name	Column Description	Validation Lookup sheet	Validation lookup sheet column	Permitted range
**CodesVLOOKUP**	LocationCode	Admin Unit Code			
**CodesVLOOKUP**	LocationName	Admin Unit Name			
**Geocodes**					
**VLookupTableUnitsVectorCollect**		Lookup for Sample Effort Units Specification			
**VLookupTableUnitsVectorCollect**	LOOKUPTABLEUNITS				
**VLookupTableUnitsVectorCollect**	CollectionTickAdultsNymphsLarvae				
**VLookupTableUnitsVectorCollect**	CONCATENATED				
**VLookupTableUnitsVectorCollect**	Unit1				
**VLookupTableUnitsVectorCollect**	Unit2				
**VLookupTableUnitsVectorCollect**	Unit3				

SUPPLEMENTRAY INFORMATION SECTION IV: Supplementary Table S4: Quality Indicators

Processes that ensure the quality of the database work on different levels: a) A standardised search string is created to ensure the accuracy and consistency in the literature review; b) A data entry template is created for complete, accurate and consistent data entry, and c) Entry restrictions are put in place within the data entry template to ensure valid and accurate data entry

Random data quality checks on 1% of the submitted data are performed after data uploads, and the quality criteria completeness, unique-ness, validity, accuracy and consistence are checked based on quality indicators for these qualities. The Table lists the fields to be included in the QI assessment: which are evaluated together and which not. The data entry tool comes with a manual to ensure ease of use and correct use. The quality indicators are calculated after the new data entry system is implemented, tested and validated, and the backlog of data has been entered into the new system. The quality indicators are calculated from the newly migrated database and apart from uniqueness, all indicators are only calculated for the new data.

Completeness

Completeness is expressed by the number of fields that are filled in. An analysis per column are made over the database to evaluate the extracted data and the data entry template. Which fields are checked can be found in the Table below. The QI for uniqueness is the proportion of complete records, as compared to the data filled from the random quality check.

Uniqueness

Uniqueness is assessed for the following: species, coordinates, VectorLifeStage, VectorSex, CollectionPlaceID, CollectionEffortStartDate, VectorCollectionMethod, VectorHostSpecies, Host Bodypart and source as a measure for the uniqueness. A duplicate constitutes a record where all these fields are the same. If the source is different (a different document) but the rest is the same, this is not counted as a duplicate. Duplicates are marked in the database for easy filtering but are not automatically removed. The QI for uniqueness is the proportion of duplicate records.

Validity and Correctness

There are specific restrictions built into the data entry template (e.g. location data entered with a dot and not a comma etc.). The QI for validity is the proportion of valid records.

Correctness

The correctness of the data is different than validity. An entered cell can be valid according to the restrictions in the data entry template but can be not correct, e.g. the species name is correctly spelled but is the wrong species. This is checked during the data quality check, and the QI for correctness is the proportion of correct records.

Accuracy and Consistency

Accuracy and consistency of new uploads is ensured by using defined upload templates. Within the template excel file there are several limitations and lists baked in to ensure the consistency of the data upload. The QI for accuracy and consistency is the proportion of records that comply with the template lists (out of those that have pre-defined lists).

Table: Overview of the Quality Indicators different fields in the data entry template.

Field Name	Comment
SourceID	Not considered during the QI evaluation.
VectorCategory	Evaluated under the QI assessment.
VectorSpeciesName	Evaluated under the QI assessment.
Country	Evaluated under the QI assessment.
LocationCode, Location Name	These are linked together and assessed as one.
Longitude E/W, Longitude (degrees), Longitude (minutes), Longitude (seconds), Longitude (decimal degrees) (X), Latitude N/S, Latitude (degrees)2, Latitude (minutes), Latitude (seconds), Latitude (decimal degrees) (Y), User Entry Longitude (X) (decimal degrees), User Entry Latitude (Y) (decimal degrees)	Evaluated together as they are duplicates.
PrecisionLocation	Evaluated under the QI assessment.
VectorLifeStage	Evaluated under the QI assessment.
VectorSex	Evaluated under the QI assessment.
NumberOfVectorsCaught	Evaluated under the QI assessment.
CollectionPlaceID	Evaluated under the QI assessment.
CollectionEffortStartDate CollectionEffortEndDate	These are linked together and are assessed as one.
VectorCollectionMethod	Evaluated under the QI assessment.
CollectionEffortP1Value, UnitsEffortP1	These are linked together and are assessed as one.
CollectionEffortP2Value, UnitsEffortP2	These are linked together and are assessed as one. These are optional and do not count towards completeness.
VectorHostSpecies, Host Bodypart	These are linked together and are assessed as one.
VectorIdentificationMethod	Evaluated under the QI assessment.
ShelteredEnvironment	Evaluated under the QI assessment.
ReportedDistributionStatus	Evaluated under the QI assessment.
PathogenName	Evaluated under the QI assessment.
PathogenDetectionMethod	Evaluated under the QI assessment.
NumberOfVectorTested	Evaluated under the QI assessment.
PositivePathogenDetection	Evaluated under the QI assessment.
PathogenComment	Not included in the QI assessment
PublicationTitle. Author,Year of Publication, Author, Source Type	These are linked together and are assessed as one.
DOI	Evaluated under the QI assessment.
VectorNetFieldStudyID	Not included in the QI assessment
URL	Not included in the QI assessment
NotesFromDataExpert	Not included in the QI assessment
Data expert name and surname for each row	Not included in the QI assessment
Submitter email (first row only)	Not included in the QI assessment

SUPPLEMENTRAY INFORMATION SECTION V: Supplementary Figures S1 – S45: Maps

Surveillance<br>Supplementary Figure S1: Invasive Mosquitoes<br>Supplementary Figure S2: Native Mosquitoes<br>Supplementary Figure S3: Biting Midges<br>Supplementary Figure S4: Sand flies<br>Supplementary Figure S5: Ticks<br>Supplementary Figure S6 a, b: Comparison of surveillance and distributions status	Distribution: Invasive Mosquitoes<br>Supplementary Figure S7: *Aedes aegypti*<br>Supplementary Figure S8: *Ae. albopictus*<br>Supplementary Figure S9: *Ae. atropalpus*<br>Supplementary Figure S10: *Ae. japonicus*<br>Supplementary Figure S11: *Ae. koreicus*
Distribution: Native Mosquitoes<br>Supplementary Figure S12: *Aedes caspius*<br>Supplementary Figure S13: *Ae. detritus/coluzzii*<br>Supplementary Figure S14: *Ae. vexans*<br>Supplementary Figure S15: *Anopheles maculipennis* s.l.<br>Supplementary Figure S16: *An. plumbeus*<br>Supplementary Figure S17: *An. superpictus*<br>Supplementary Figure S18: *Coquillettidia richiardii*<br>Supplementary Figure S19: *Culex modestus*<br>Supplementary Figure S20: *Cx. pipiens/torrentium*	Distribution: Biting Midges<br>Supplementary Figure S21: *Culicoides chiopterus*<br>Supplementary Figure S22: *C. dewulfi*<br>Supplementary Figure S23: *C. imicola*<br>Supplementary Figure S24: *C. kingi*<br>Supplementary Figure S25: *C. newsteadi*<br>Supplementary Figure S26: *C. pulicaris /lupicaris*<br>Supplementary Figure S27: *C. punctatus*.<br>Supplementary Figure S28: *C. obsoletus/scoticus*
Distribution: Sand flies<br>Supplementary Figure S29: *Phlebotomus alexandri*<br>Supplementary Figure S30: *P. ariasi*<br>Supplementary Figure S31: *P. mascittii*<br>Supplementary Figure S32: *P. neglectus*<br>Supplementary Figure S33: *P. papatasi*<br>Supplementary Figure S34: *P. perfiliewi*<br>Supplementary Figure S35: *P. perniciosus*<br>Supplementary Figure S36: *P. sergenti*<br>Supplementary Figure S37: *P. similis*<br>Supplementary Figure S38: *P. tobbi*	Distribution: Ticks<br>Supplementary Figure S39: *Dermacentor reticulatus*<br>Supplementary Figure S40: *Hyalomma lusitanicum*<br>Supplementary Figure S41: *H. marginatum*<br>Supplementary Figure S42: *Ixodes persulcatus*<br>Supplementary Figure S43: *I. ricinus*<br>Supplementary Figure S44: *Ornithodoros erraticus*<br>Supplementary Figure S45: *Rhipicephalus sanguineus*

Vector Group Surveillance Activity Maps

Figures S 1 to 5 present the summaries of reported surveillance activities from 2015 to 2019 for each vector group along with commentary descriptions. Two maps are presented for each group: a) Seasonal active surveillance = the number of months per year for which active surveillance (so not including passive surveillance through Citizen Science reporting) is reported and b) surveillance effort. For each admin unit, surveillance was coded by type with the highest code reflecting the highest effort as shown on the map legend. The highest surveillance type that occurred in an administrative area during 2015–2019 is depicted, with lower type categories contributing to the effort score with a lower weight as presented in the table at the end of the commentary text. The highest type-code that occurred during the period 2015–2019 decides the colour (hue) of the admin unit. The frequency with which it occurred decides the saturation of the colour: a deeper colour indicates more frequent surveillance. If lower surveillance types also occurred in the same unit, these also contribute to the saturation, but with a lower weight than the highest code which decides the hue.

Each map also shows the weighting table in the right-hand column, interpreted as follows:

	Highest level				
**Level**	**1**	**2**	**3**	**4**	**5**
**1**	1	0	0	0	0
**2**		1	1	0.5	0.25
**3**			1	0.5	0.25
**4**				1	0.5
**5**					1

All Level one (citizen science) scores are unweighted or discounted if reported to include active surveillance. All other level two and three surveillance scores are unweighted. If the most intensive surveillance in a polygon is level 4 or 5, scores for the less intensively sampled periods are progressively reduced as sampling level falls.

Figures S 6, a and b show a comparison between surveillance activities and distribution status to assess where the two match (i.e. there is surveillance and data, or where there is no surveillance and no data). A mismatch implies that either there are no data available or that the data have not been located.

Tables S 5 to 43: Each table presents a brief description of the species, and the VectorNet map for March 2021, with commentary on the distributions shown. Each table also provides the means to compare the VectorNet (VN) map with the GBIF or VectorBase (VBASE) records using a simplified map showing the presence and absence, each as a single category, with the point records from GBIF or VectorBase overlain. A summary table of the number of presence and absence polygons with and without GBIF or VectorBase records is also provided. An example interpretation is shown as follows:

GBIF or VectorBase Present	VectorNet Present	VectorNet Absent	VectorNet No Data	Total
**No**	22	807	666	**1495**
**Yes**	2	9	0	**11**
**Total**	**24**	**816**	**666**	**1506**

There are 11 polygons with GBIF or VectorBase records. There are no GBIF or VectorBase records in 22 polygons where VectorNet records the species as present, 807 polygons where VectorNet records the species as absent and 666 polygons where VectorNet has no data. There are GBIF or VectorBase records in 2 polygons where VectorNet records the species and present, 9 polygons where VectorNet records the species as absent, and 0 records where VectorNet has no data.

Figures S 7 to 45 show the VectorNet March 2021 distribution mapd of the species / species group and a comparison with records from an alternative database (GBIF or Vectorbase, depending on which was the most populated for the species / species group).

Supplementary Figure S1: SURVEILLANCE OF INVASIVE MOSQUITOES

Seasonal active surveillance

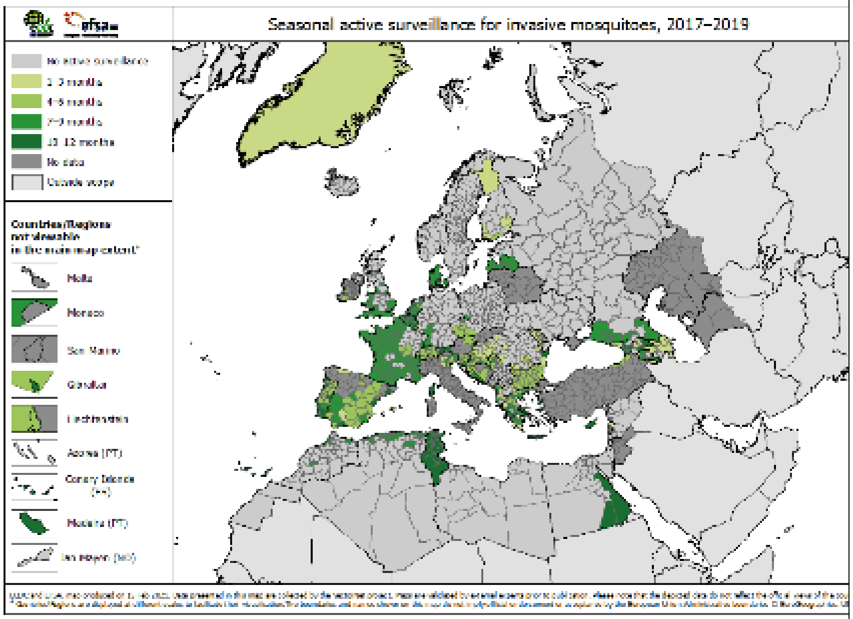

Surveillance for invasive mosquitoes is concentrated in Western Europe, the Balkans, the Caucasus and the western part of North Africa, and the reported surveillance effort is generally high.

There is a need for strengthening the low intensity of surveillance efforts in countries where *Ae. albopictus* and other invasive species might be present but undetected in many administrative units. Assessing abundance and seasonality should be prioritised to help prevent/control possible disease outbreaks in areas were *Ae. albopictus* is well established and that are receptive to pathogen introduction, such as southern France and parts of Greece.

Surveillance effort

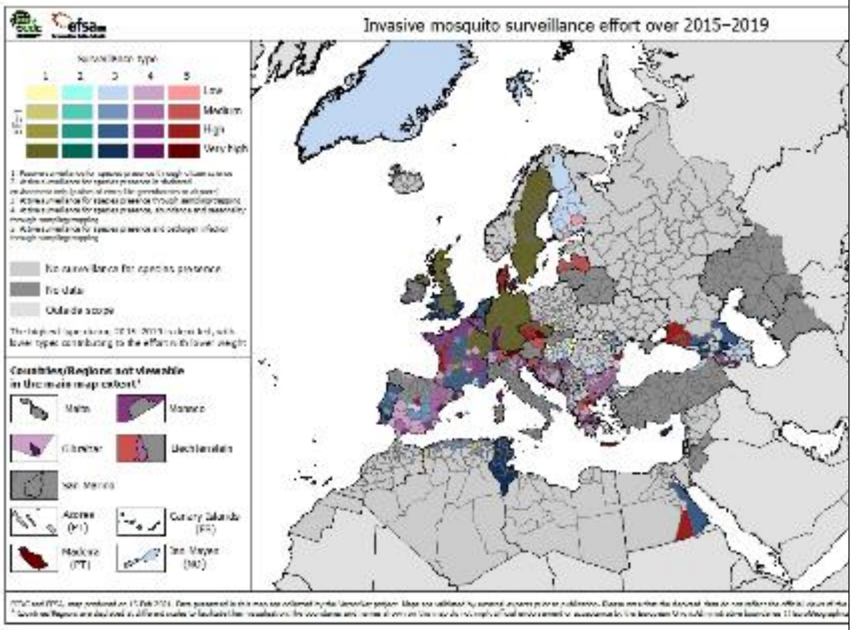

Invasive mosquito surveillance by citizen science is predominant in northern and central Europe whilst various forms of active surveillance tend to be most common in south-western Europe and the Balkans. The main surveillance activities are for *Ae. albopictus* for its presence (introduction and spread), and less frequently for its abundance as part of disease control. Given the current known distributions, there are obvious gaps in the reported surveillance data in Italy, Turkey, and Crimea.

Effort Weighting

	Highest level				
**Level**	**1**	**2**	**3**	**4**	**5**
**1**	1	0	0	0	0
**2**		1	1	0.5	0.25
**3**			1	0.5	0.25
**4**				1	0.5
**5**					1

Surveillance Effort Categories

1 = passive surveillance for species presence through citizen science

2 = active surveillance for species presence in sheltered environment only (points of entry like greenhouses or airport building)

3 = active surveillance for species presence through sampling/trapping (with or without passive surveillance)

4 = active surveillance for species presence, abundance and seasonality through sampling/trapping (with or without passive surveillance)

5 = active surveillance for species presence and pathogen infection through sampling/trapping (with or without passive surveillance)

Supplementary Figure S2: SURVEILLANCE OF NATIVE MOSQUITOES

Seasonal active surveillance

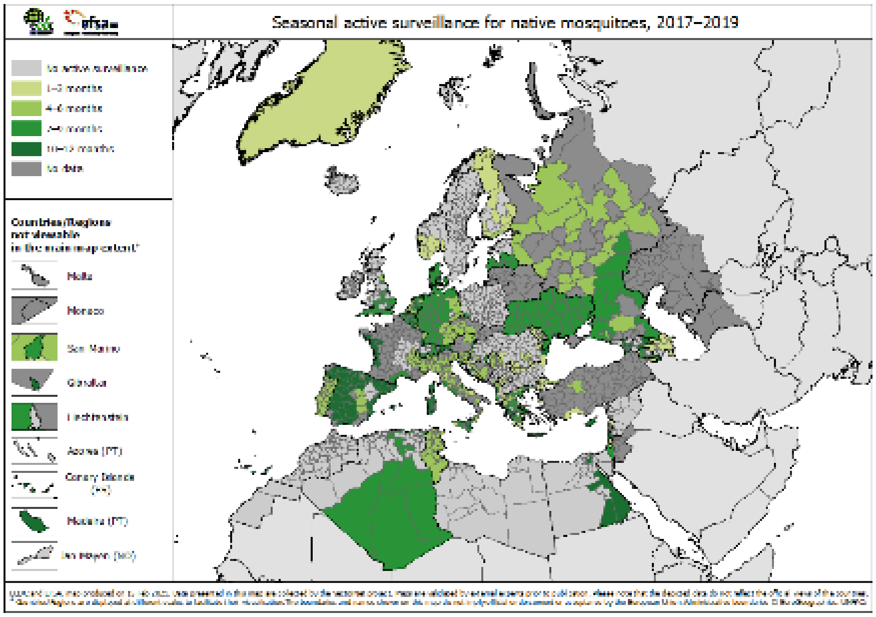

Surveillance effort

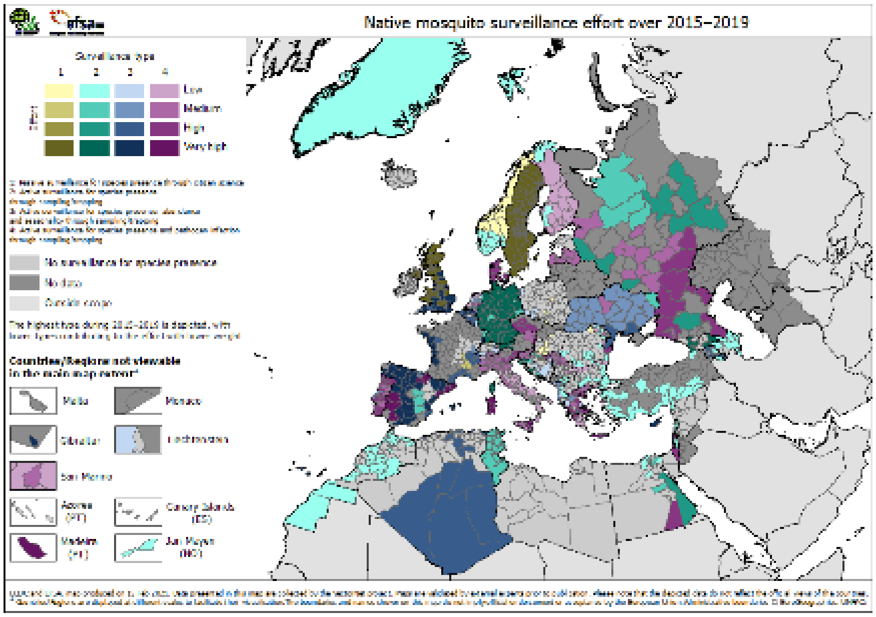

The surveillance coverage appears to be quite patchy in some countries, possibly because mosquito control activities and their associated surveillance are often implemented over relatively small areas. The current datasets suggest that there are surveillance data gaps in some countries and that there is a need for strengthening the intensity of surveillance efforts in areas were mosquito-borne pathogens are known to circulate or be at risk of circulating.

Native mosquito surveillance techniques are very diverse and include: (1) presence data only, mainly based on citizen science projects; and (2) abundance and seasonality data acquired in areas where pest species occur and are controlled (e.g. parts of France) or more widely for scientific purposes. These abundance and seasonality data are associated with pathogen screening, both in areas where disease outbreaks are more frequent (West Nile infections in particular) and in currently disease-free countries.

Effort Weighting

	Highest level			
**Level**	**1**	**2**	**3**	**4**
**1**	1	0	0	0
**2**		1	0.5	0.25
**3**			1	0.5
**4**				1

Surveillance Effort Categories

1 = passive surveillance for species presence through citizen science

2 = active surveillance for species presence through sampling/trapping (with or without passive surveillance)

3 = active surveillance for species presence, abundance and seasonality through sampling/trapping (with or without passive surveillance)

4 = active surveillance for species presence and pathogen infection through sampling/trapping (with or without passive surveillance)

The highest surveillance type that occurred in an administrative area during 2015–2019 is depicted, with lower type categories contributing to the effort score with a lower weight. Weights were as follows:

Supplementary Figure S3: SURVEILLANCE OF BITING MIDGES

Seasonal active surveillance

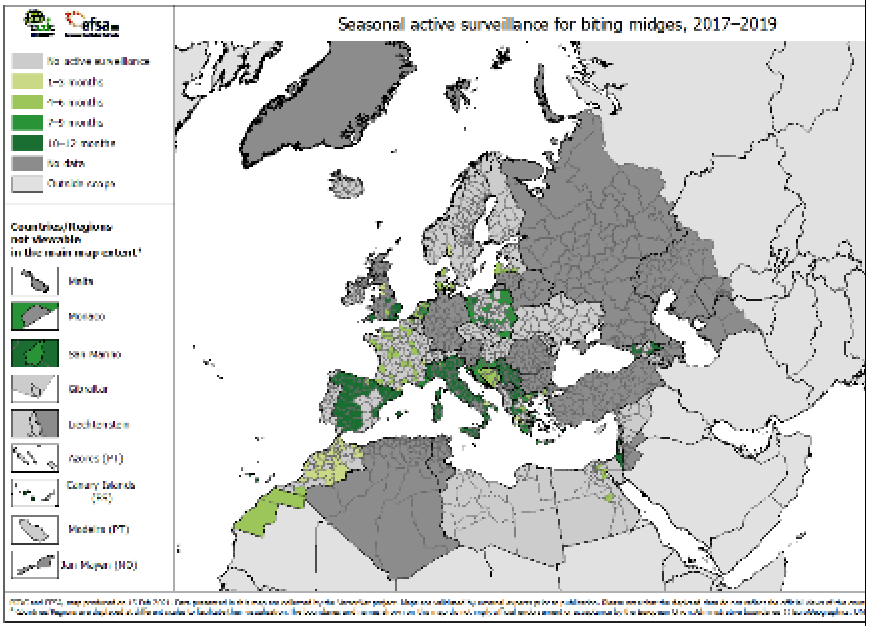

Surveillance effort

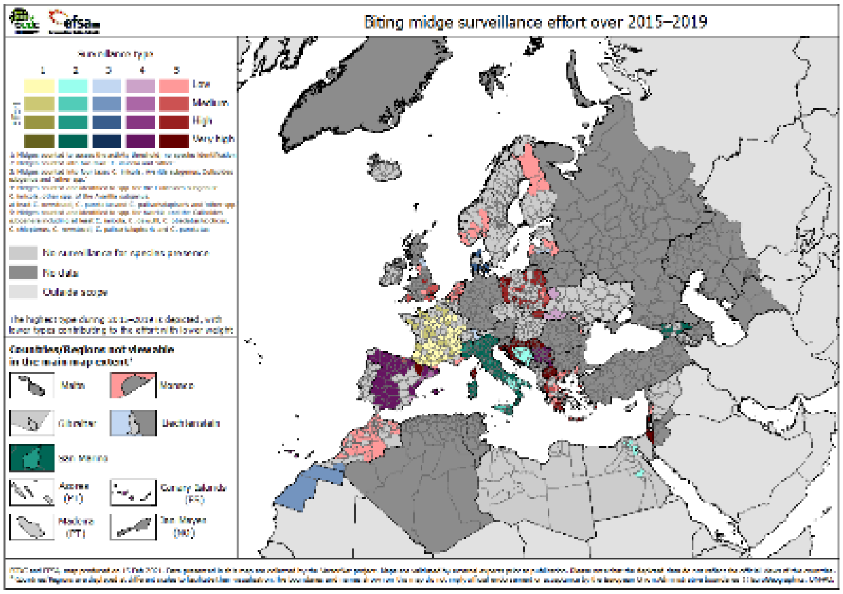

The most sustained annual surveillance is reported in Spain, Italy and the Balkans.

Midge surveillance, more than for other vector groups, has varied considerably during the past 15 years: most *Culicoides* surveillance activities in the VectorNet geographical area were initiated in response to bluetongue epizootics, and in parts of Spain, due to the African horse sickness outbreaks in 1987–1990.

Midge surveillance methodology varies considerably according to country. The most widespread method is full species identification (level 5), though this is not the case in Western Europe. Data for midge surveillance activities have been acquired for fewer countries than is the case for the other vector categories, but where data are available, surveillance occurs over a wide range, and is comparatively intensive. Data are still to be acquired for some countries, where information does exist.

Effort Weighting

	**Highest level**				
**Level**	**1**	**2**	**3**	**4**	**5**
**1**	1	0.5	0.33	0.25	0.2
**2**		1	0.66	0.5	0.4
**3**			1	0.75	0.6
**4**				1	0.8
**5**					1

Surveillance Effort Categories

1 = surveillance, with midges counted only to assess the activity threshold (*e.g.* more or less than 5 parous females), without species identification.

2 = surveillance implemented, with midges counted and identified into two taxa: *C. imicola* and 'other species'

3 = surveillance, with midges counted and identified into four taxa: *C. imicola*, other species of the *Avaritia* subgenus (= 'the *Obsoletus* group'), species of the *Culicoides* subgenus (= 'the *Pulicaris* group') and 'other species'

4 = surveillance, with midges counted and identified to species for the Culicoides subgenus: C. imicola, other species of the Avaritia subgenus (= 'the *Obsoletus* group'), at least *C. newsteadi, C. pulicaris/C. lupicaris* and *C. punctatus* and 'other species'

5 = surveillance, with midges counted and identified to species for at least the Avaritia and the Culicoides subgenera: results including at least *C. imicola, C. obsoletus/C. scoticus, C. dewulfi*, and *C. chiopteru,s*,as well as *C. newsteadi, C. pulicaris/C. lupicaris* and *C. punctatus.*

Supplementary Figure S4: SURVEILLANCE OF SAND FLIES

Seasonal active surveillance

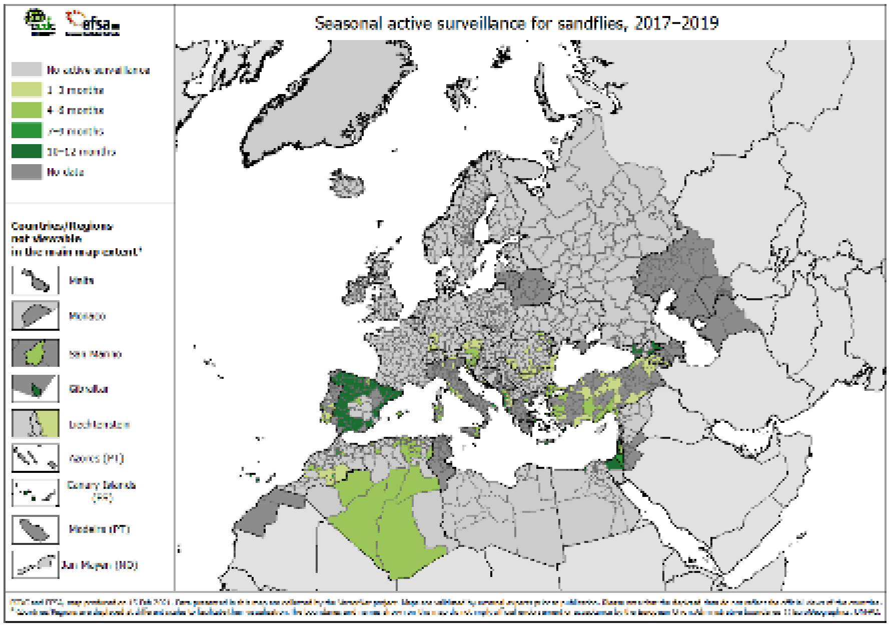

Surveillance effort

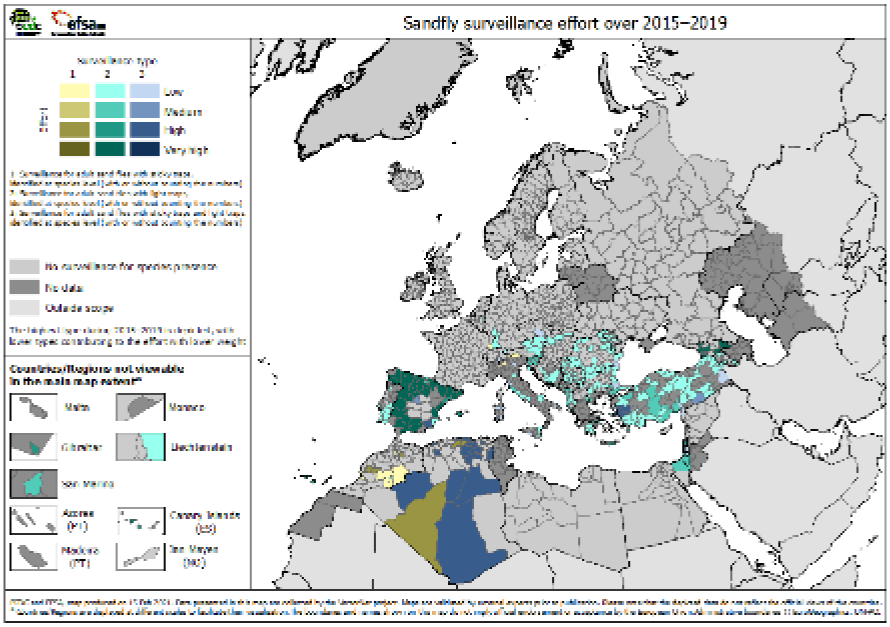

In terms of general surveillance effort, the sand fly surveillance is not only the least widespread (reflecting their comparatively restricted range), but is also implemented least intensively.

There are many apparent gaps in surveillance in parts of the Balkans, much of North Africa and Syria, and some Mediterranean regions, while there are also significant data gaps on surveillance effort in some countries.

Sand fly surveillance north and east of the Mediterranean predominantly involves light-trapping, with species identification, but in North Africa also incorporates sticky-trapping.

Effort Weighting

	**Highest level**		
**Level**	**1**	**2**	**3**
**1**	1	1	0.5
**2**		1	0.5
**3**			1

Surveillance Effort Categories

1 = surveillance for adult sand flies with sticky traps, identified at species level (with or without counting the numbers)

2 = surveillance for adult sand flies with light traps, identified at species level (with or without counting the numbers)

3 = surveillance for adult sand flies with sticky traps and light traps, identified at species level (with or without counting the numbers)

The highest surveillance type that occurred in an administrative area during 2015–2019 is depicted, with lower type categories contributing to the effort score with a lower weight. Weights were as follows:

Supplementary Figure S5: SURVEILLANCE OF TICKS

Seasonal active surveillance

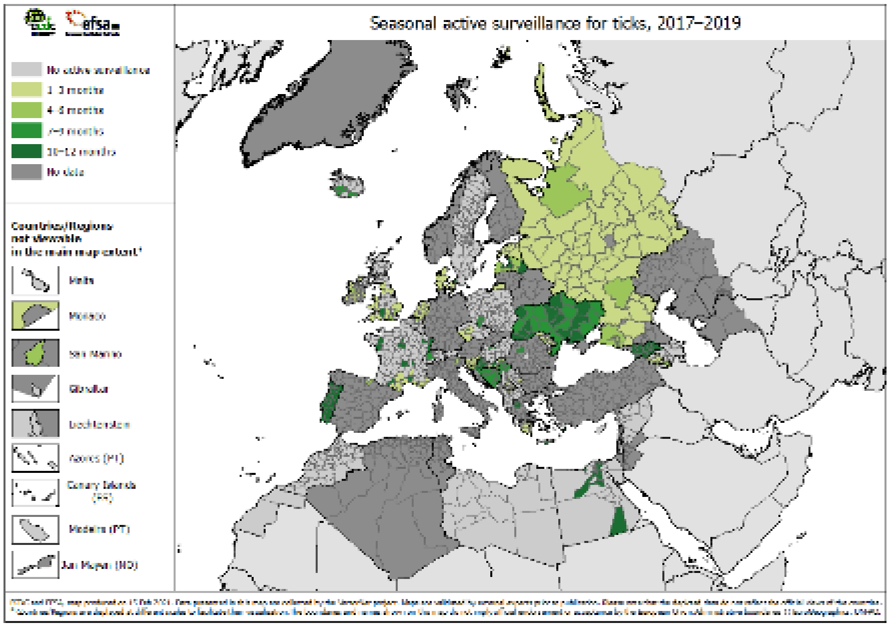

Surveillance effort

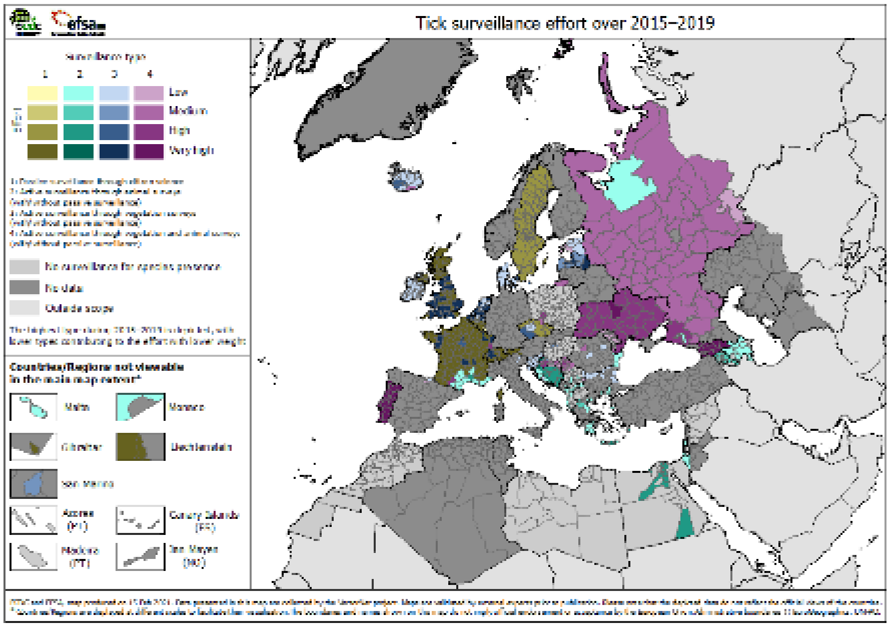

Tick surveillance methodology is variable, ranging from citizen science methods in the north and west of Europe, to the more intensive vegetation and animal monitoring in the eastern countries and in Portugal. Though data from some major countries is missing, it is clear that tick surveillance is very widespread but that the surveillance effort employed tends towards the lower end of the recorded spectrum.

The most activity for surveillance across Europe relates to the main risks posed by *Ixodes ricinus* and Lyme/tick borne encephalitis or *Hyalomma marginatum* and CCHF.

There are data gaps for some of the larger countries of central, northern and western Europe, where the available distribution data suggests that surveillance does take place. Surveillance activity in the Balkans is good for some countries, whilst in others there are no data or only local/regional surveillance.

Effort Weighting

	Highest level			
**Level**	**1**	**2**	**3**	**4**
**1**	1	0	0	0
**2**		1	1	0.5
**3**			1	0.5
**4**				1

Surveillance Effort Categories

1 = passive surveillance through citizen science

2 = active surveillance through animal surveys (with/without passive surveillance)

3= active surveillance through vegetation surveys (with/without passive surveillance)

4= active surveillance through vegetation AND animal surveys (with/without passive surveillance)

Supplementary Figure S6a, Comparison of surveillance and distributions status for vector groups

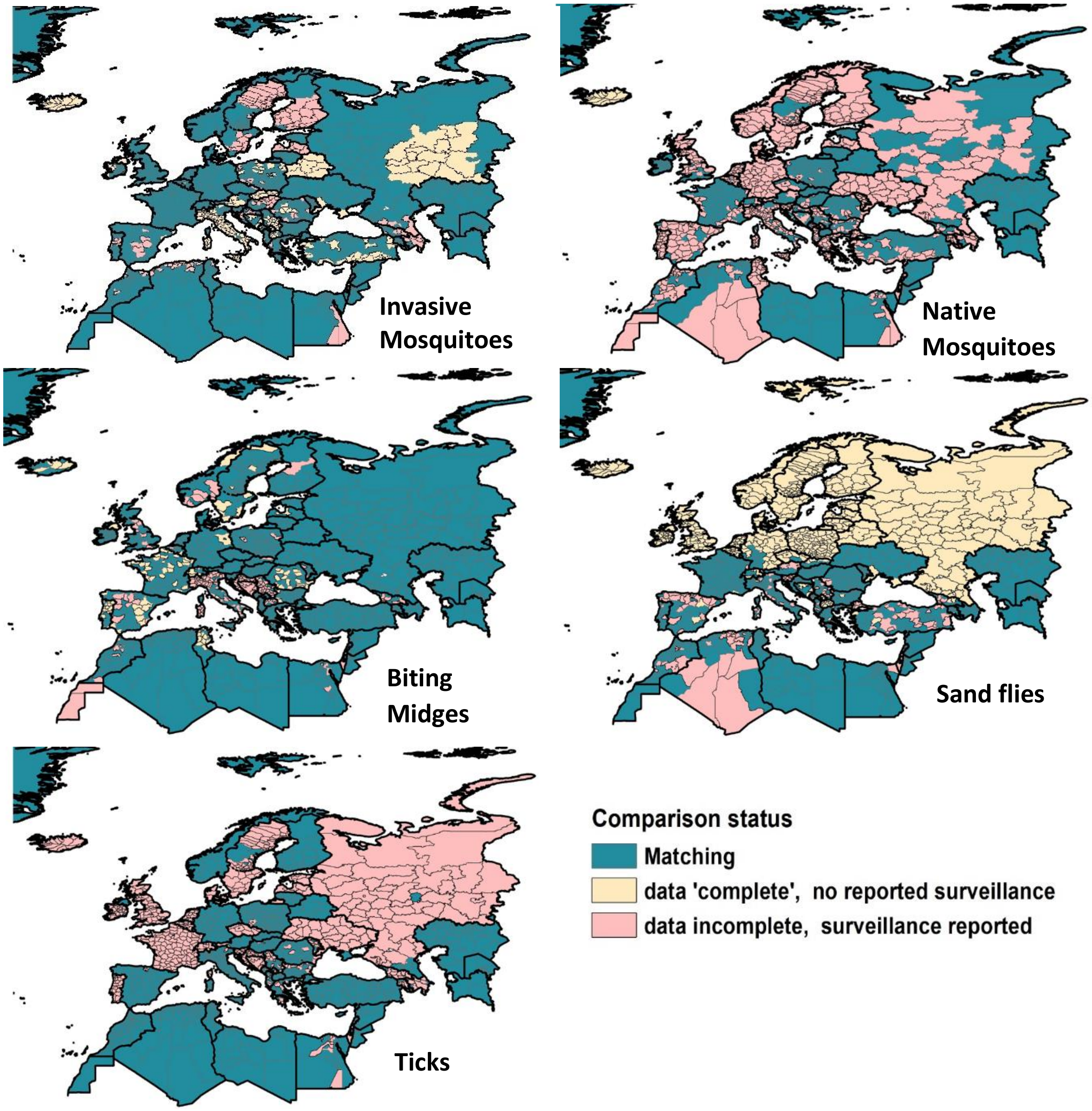

Supplementary Figure S6b, Comparison of surveillance and distributions status for vector group representative species

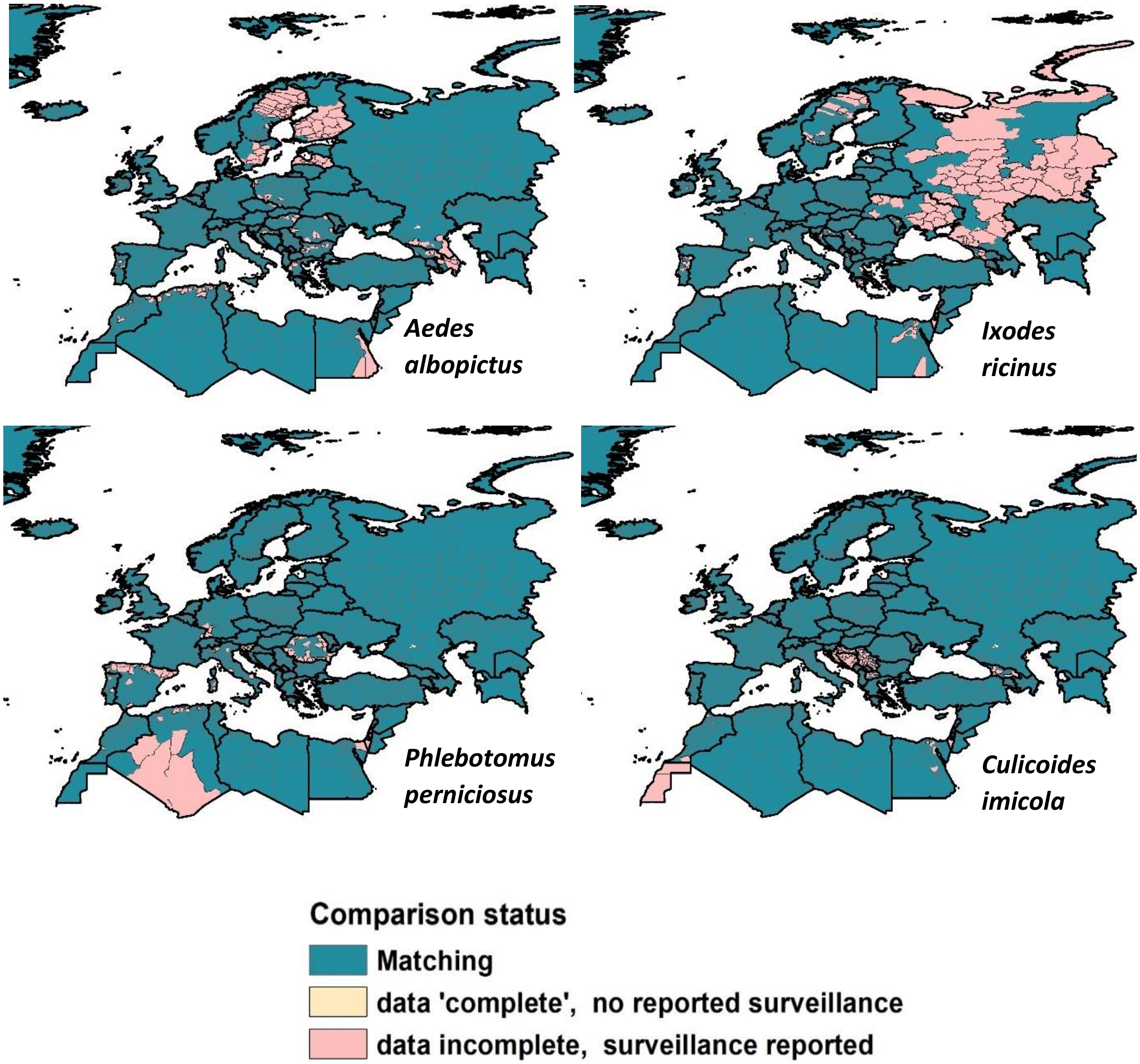

Supplementary Figure S7 *Aedes aegypti*

The yellow fever mosquito *Ae. aegypti* originates from Africa but was historically present over decades in all countries surrounding the Mediterranean Basin, where it established following introductions via ships, but from where it disappeared in the mid-twenty Century (cf Schaffner & Mathis, 2014 doi: 10.1016/S1473-3099(14)70834-5.). *Aedes aegypti* is the major worldwide vector species for dengue, chikungunya, Zika and yellow fever viruses (cf Schaffner et al., 2013 https://doi.org/10.1111/1469-0691.12189). The species was associated with the large outbreak of dengue fever that occurred in 2012 in the Portuguese Autonomous Region of Madeira (cf Sousa et al 2012 Euro Surveill. 2012;17(49):pii=20333).

VN MAP MARCH 21

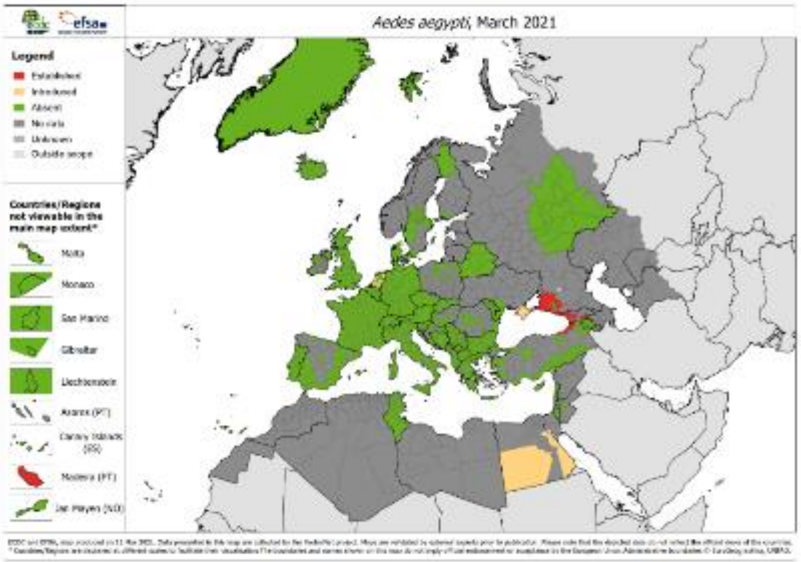

Comparison with GBIF records (in Brown)

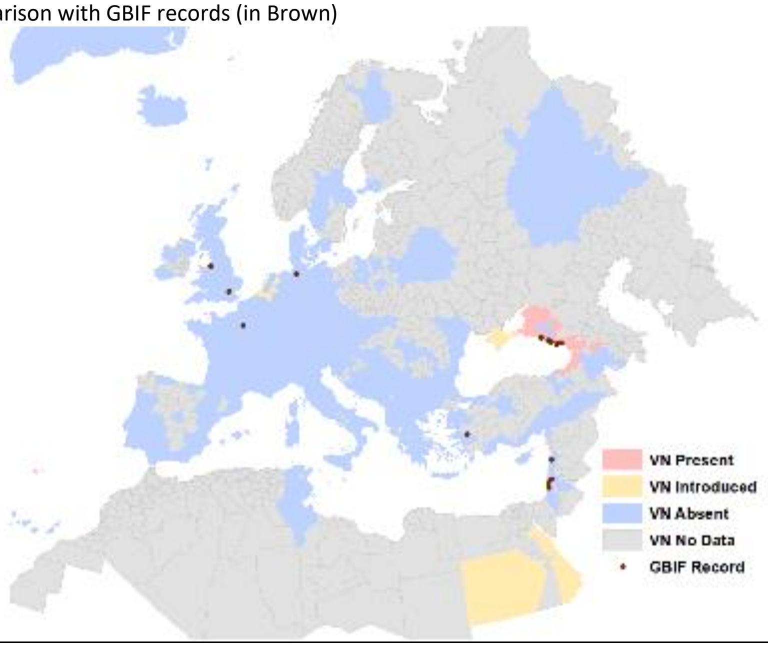

GBIF Present	VN Present	VN Absent	VN No Data	Total
**No**	22	807	666	**1495**
**Yes**	2	9	0	11
**Total**	**24**	**816**	**666**	**1506**

This invasive species has a pan global distribution. Within the VectorNet region, it has become established along the Black sea coast and on Madeira. Its establishment is not yet confirmed in Egypt, but is highly probable. Introductions to other places occur from time to time but control actions and/or climatic conditions do not lead to establishment of a viable mosquito population; such reports disappear from the maps if older than 5 years. Exceptional introduction events (*e.g.* single specimens or indoor records) are not reported by VectorNet (*i.e.* France, Germany, UK). The high number of negative areas stem from the widespread surveillance of *Ae. albopictus*.

Occurrences reported by GBIF in northern France, Germany, Israel, Turkey, UK) are not substantiated. Large VectorNet yellow or red areas (Egypt and Russia) are due to the large size of the mapped admin units, while they refer to much more localised populations.

Supplementary Figure S8: *Aedes albopictus*

The tiger mosquito *Ae. albopictus* is native to south-east Asia and is invading the European continent since the 1990s. It was first introduced into Albania (late 1970s) and Italy (1990s), from where it has progressively spread to neighbouring countries (cf Medlock et al 2015 doi:10.1017/S0007485315000103). This major invasive species is a nuisance species and a significant vector of dengue, chikungunya and Zika viruses, and of *Dirofilaria* parasites (cf Schaffner et al., 2013, https://doi.org/10.1111/1469-0691.12189)

VN MAP MARCH 21

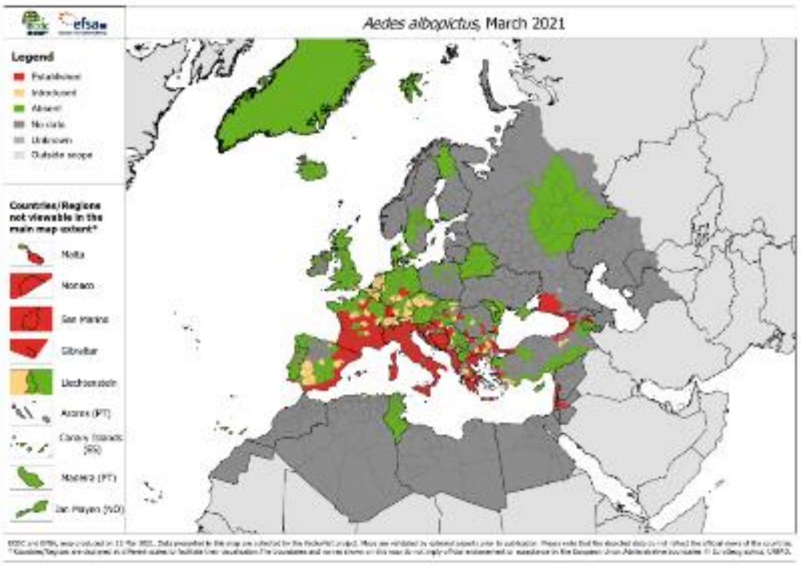

Aedes albopictus is now widespread all around the Mediterranean and Black Sea and is spreading North. The VectorNet maps show also numerous introduction locations as yellow areas and where population often eventually becomes established in subsequent mapping periods. This illustrates the spread of the species. The widely implemented active and/or passive surveillance programmes have provided many true absence records (green areas).

Comparison with GBIF records (in Brown)

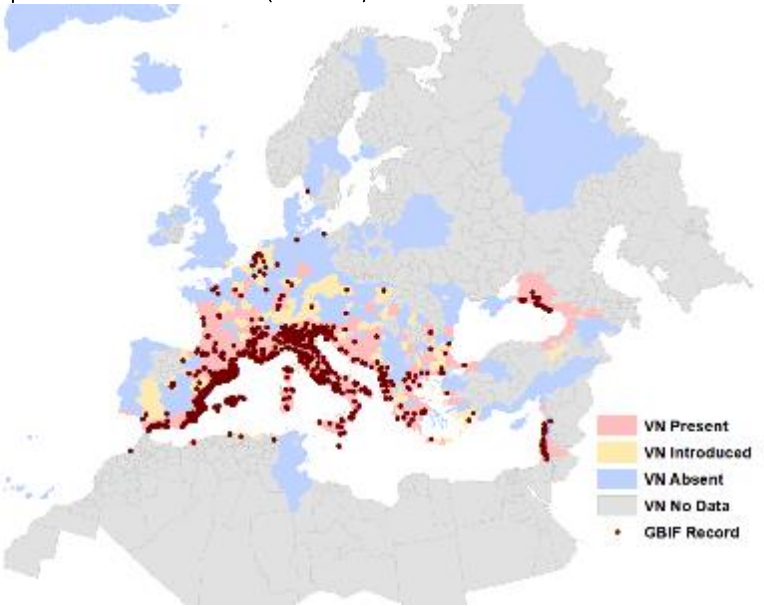

GBIF Present	VN Present	VN Absent	VN No Data	Total
**No**	187	424	637	**1248**
**Yes**	243	12	3	**258**
**Total**	**430**	**436**	**640**	**1506**

The surveillance data for *Ae. albopictus* are well reported to VectorNet, which shows more presence areas than GBIF does (e.g. France, Balkan, Caucasus). In some cases, large VectorNet yellow or red areas (e.g. southern Russia) are due to the large size of the mapped admin units, while the presence is much more localised. Conversely, established populations are sometimes hard to see on the maps, due to the very small size of the mapped admin units (e.g. Algeria, Morocco).

The high number of GBIF reports might be related to the nuisance generated by this mosquito species and to its strong connexion with human settlements.

Some presence data reported by GBIF are related to as yet unsubstantiated records (e.g. north-west France, northern Germany, Sweden).

Supplementary Figure S9: *Aedes atropalpus*

The American rock pool mosquito *Ae. atropalpus* is native to Eastern North America. Although originally limited to rock pool areas, its utilisation of used tyres as an alternative larval development site resulted in its spread in North America and introductions into the European continent (cf Medlock et al 2015 doi:10.1017/S0007485315000103). This species is not considered to be an important vector of pathogens in the field, despite isolation of West Nile virus in field populations. Laboratory experiments demonstrate some competence to transmit several encephalitis viruses (cf Scholte et al., 2012, European Mosquito Bulletin 30 (2012), 1-14).

VN MAP MARCH 21

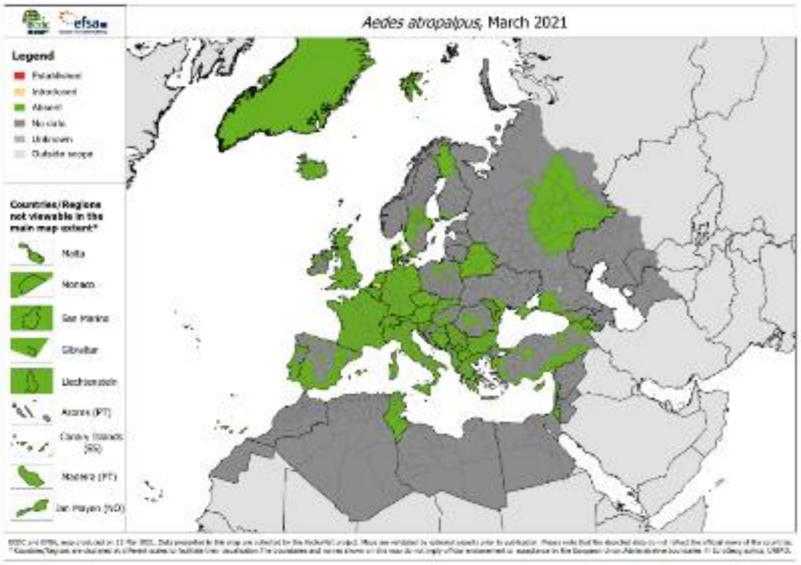

Occasional introductions and short-term establishments have occurred in Europe since the 1990s, but control measures implemented to eliminate *Ae. albopictus*, which was introduced simultaneously, has eliminated these populations (France, Italy, Netherlands). The areas appearing in yellow on the VectorNet map refer to such events that occurred less than 5 years ago. The high number of negative areas stem from the surveillance of *Ae. albopictus*.

Comparison with GBIF records (in Brown)

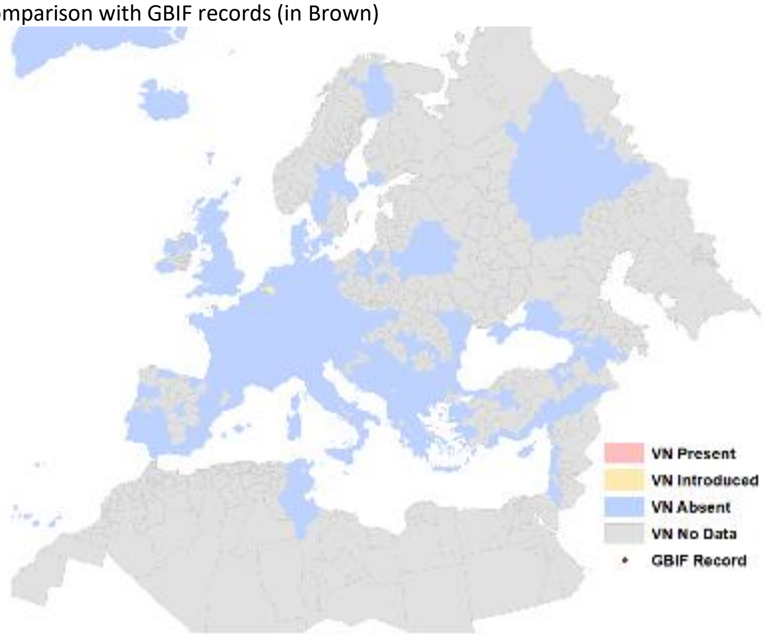

GBIF Present	VN Present	VN Absent	VN No Data	Total
No	0	828	678	**1506**
Yes	0	0	0	**0**
**Total**	**0**	**828**	**678**	**1506**

The absence of records in GBIF fits with the current VectorNet reported surveillance results. The small and briefly established populations did not lead to any GBIF reports.

Supplementary Figure S10: ***Aedes japonicus***

The Asian rock pool mosquito or Asian bush mosquito *Ae. japonicus* originates from East Asia where four subspecies occur, but only one of these, *Ae. japonicus japonicus*, is invasive in Europe. It is nowadays widely present in Northern America and has been successfully spreading throughout the European continent since the early 2000s (cf Medlock et al 2015 doi:10.1017/S0007485315000103). The species is not considered to be an important disease vector in its native Asian range. It may also be involved in West Nile virus transmission, and shows some level of vector competence for other viruses such as dengue and chikungunya (cf Schaffner et al., 2013, https://doi.org/10.1111/1469-0691.12189).

VN MAP MARCH 21

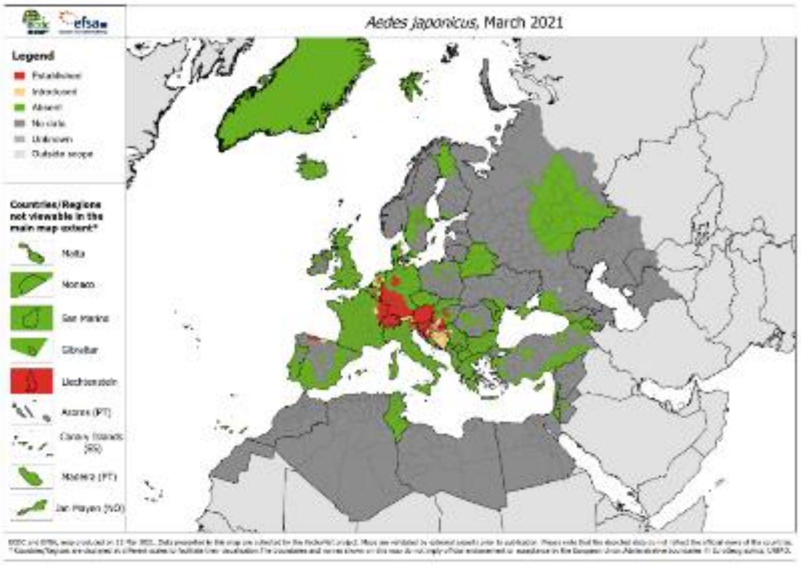

From a patchy distribution based on geographically distant introduction events, the species' spread led to the current widely colonised area in central Europe, from the North Sea coast to the Adriatic Sea coast. From there, and in all directions, the distribution continues spreading, as showed by the yellow patches where has been recorded for less than 5 years. In addition, isolated populations have been recently reported in Spain and in Romania. The high number of negative areas stem from the surveillance of *Ae. albopictus*.

Comparison with GBIF records (in Brown)

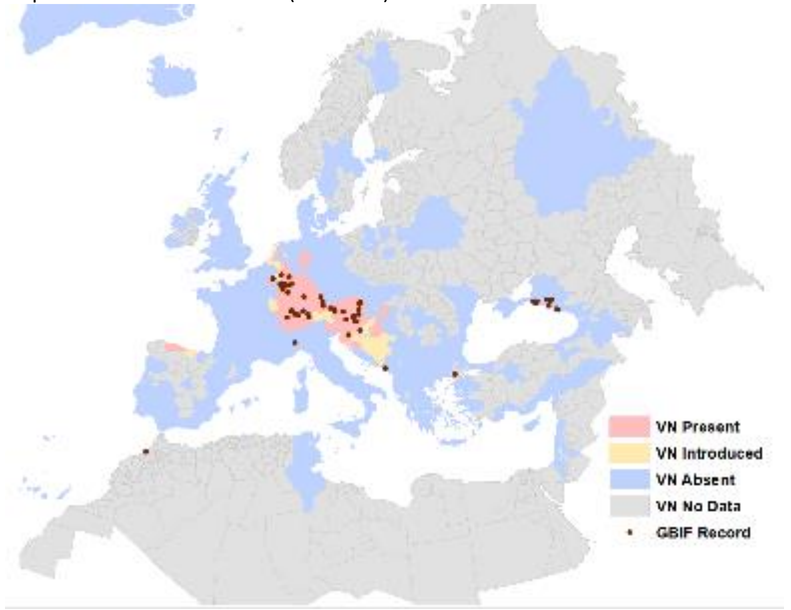

GBIF Present	VN Present	VN Absent	VN No Data	Total
No	52	773	652	**1477**
Yes	21	5	3	**29**
Total	**73**	**778**	**655**	**1506**

The high number of GBIF reports from its known distribution area (central Europe) might be related to the association of larval habitats with human settlements, as well as, to a certain degree, nuisance generated by the species.
The other reports in (Morocco, southern France, Greece, southern Russia and Georgia) are not substantiated by any scientific report

Supplementary Figure S11: *Aedes koreicus*

The Korean mosquito *Ae. koreicus* is native from South-East Asia and was encountered for the first time in Europe in 2008, and later in Italy. The species has been suggested to be a possible vector for Japanese encephalitis virus in parts of Russia; it may contribute to the transmission of *Dirofilaria* parasites (cf Medlock et al 2015 doi:10.1017/S0007485315000103)

VN MAP MARCH 21

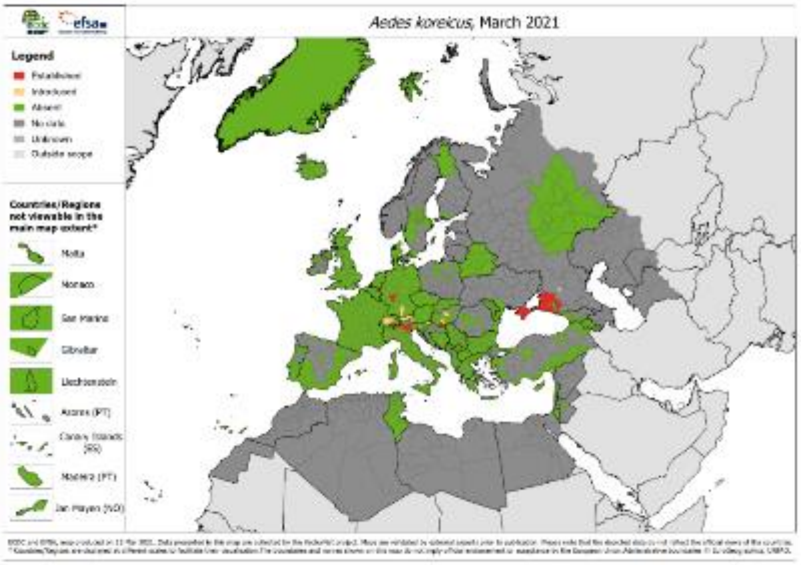

Aedes koreicus shows a patchy distribution with several established populations is isolated small areas throughout the European subcontinent. Nowadays, new introduction events are reported on a regular basis (yellow areas).
The high number of negative areas stem from the surveillance of *Ae. albopictus*.

Comparison with GBIF records (in Brown)

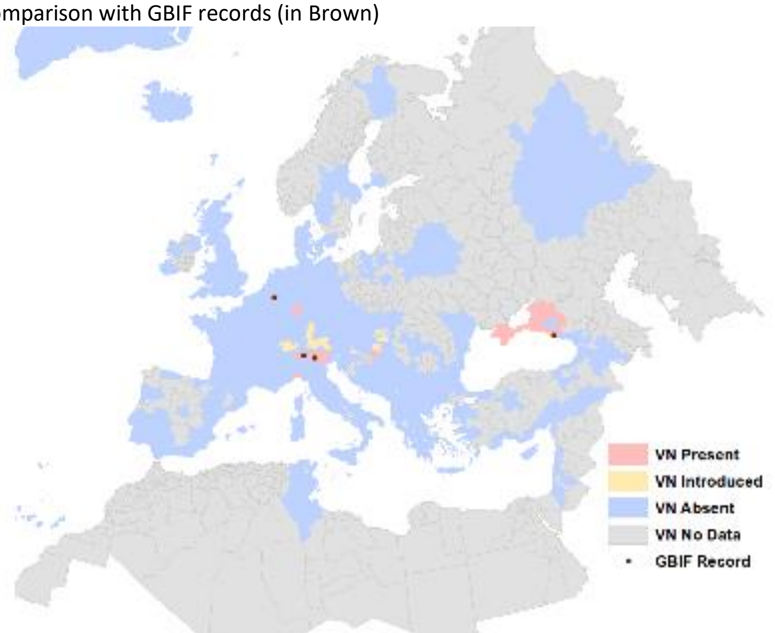

GBIF Present	VN Present	VN Absent	VN No Data	Total
**No**	9	826	667	**1502**
**Yes**	4	0	0	**4**
**Total**	**13**	**826**	**667**	**1506**

The few GBIF records fit well with the VectorNet distribution records. Some wide VectorNet areas with suggested established populations are related to the large size of the mapped admin unit (*e.g*. southern Russia) while the presence is much more localised.

Supplementary Figure S12: *Aedes caspius*

The native species *Aedes caspius* is the primary European salt marsh mosquito that develops in coastal or inland brackish marshes, and, in southern Europe, in rice fields and flood plains (cf Becker et al., 2010). DOI: 10.1007/978-3-540-92874-4. The species is responsible for intense nuisance in the areas where it proliferates, and therefore is frequently targeted by control programmes (e.g. France, Greece, Italy, Spain). *Aedes caspius* may contribute to the transmission of Rift Valley fever, West Nile and Tahyna viruses; it may also play a role in the spread of tularaemia and myxomatosis (cf Becker et al., 2010,. DOI: 10.1007/978-3-540-92874-4)

VN MAP MARCH 21

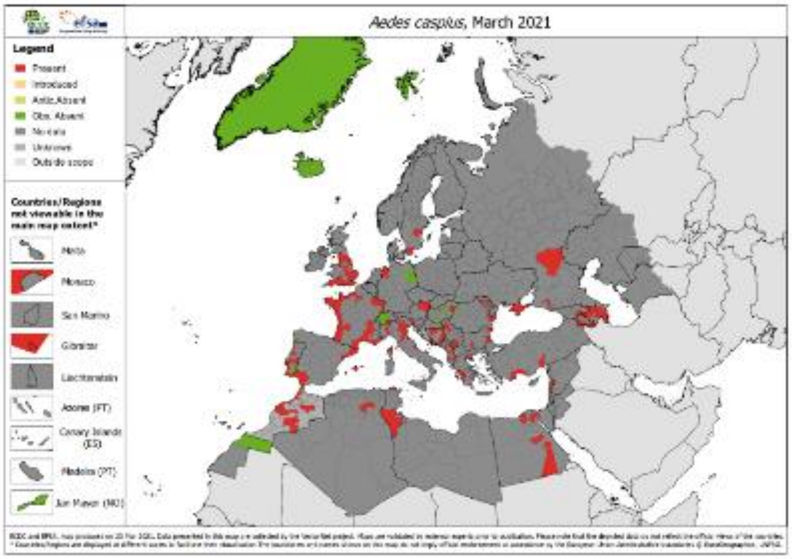

The VectorNet map shows a patchy distribution, fitting with the known species' requirements (brackish marshes). Inland populations are mainly related to the occurrence of continental brackish marshes (e.g. Austria, Egypt, north-eastern France, Hungary, Tunisia). The species is absent from most northerly latitudes but is present on Southern Sweden. Few true absences have been reported.

Comparison with GBIF records (in Brown)

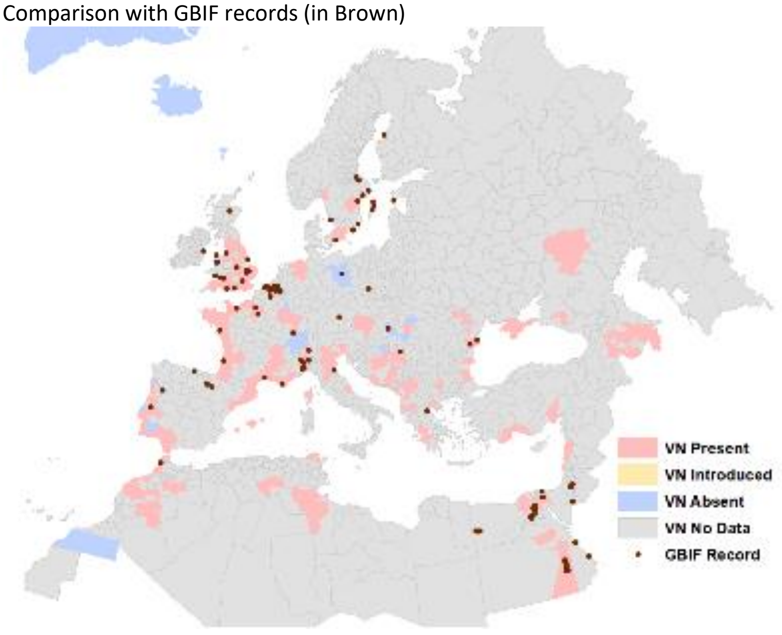

About a third of GBIF records match the VectorNet distribution, and there almost 40 polygons where GBIF has records but Vectornet does not. This could reflect an underreporting by VectorNet (e.g. Egypt, Spain, Sweden) or misidentification

GBIF Present	VN Present	VN Absent	VN No Data	Total
No	165	41	1238	**1444**
Yes	23	1	38	**62**
**Total**	**188**	**42**	**1276**	**1506**

Supplementary Figure S13: ***Aedes detritus/coluzzii***

Aedes detritus/coluzzii is a complex of two sibling species which have been described based on molecular studies; no morphological diagnostic features are described. To date, only few studies have investigated the populations with molecular tools. Therefore, they are mapped together. *Aedes detritus/coluzzii* is the second European salt marsh mosquito that develops in coastal or inland brackish marshes (cf Becker et al., 2010,. DOI: 10.1007/978-3-540-92874-4)). The species is responsible of intense nuisance in the areas where it proliferates, and is therefore frequently targeted by control programmes (e.g. France, Italy, Spain) The species It is a potential vector for RVF (cf Moutailler S, et al, http://doi.org/10.1089/vbz.2008.0009.

VN MAP MARCH 21

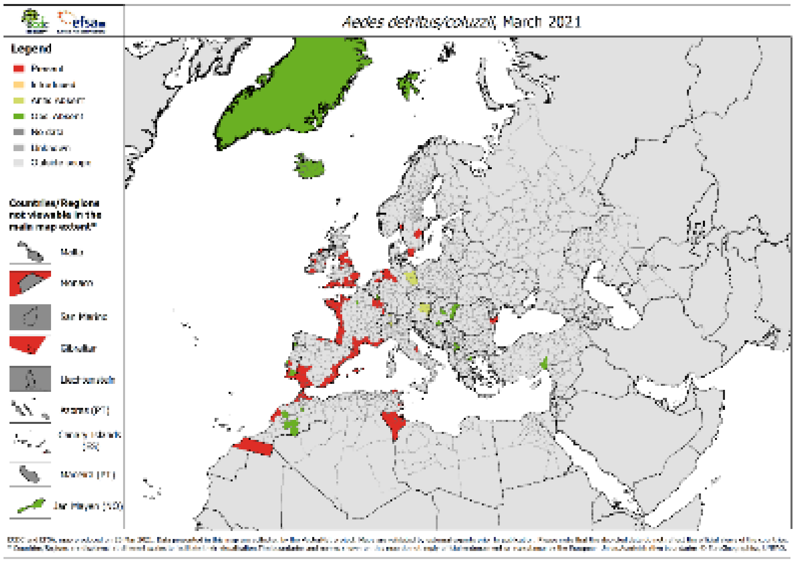

The VectorNet map shows a mainly coastal distribution, fitting with the known species requirements (brackish marshes). The rather few Inland populations are related to the occurrence of continental brackish marshes (e.g. north-eastern France, Tunisia). The species is absent from most northerly latitudes but is present is southern Sweden. Few true absences are reported.

Comparison with GBIF records (in Brown)

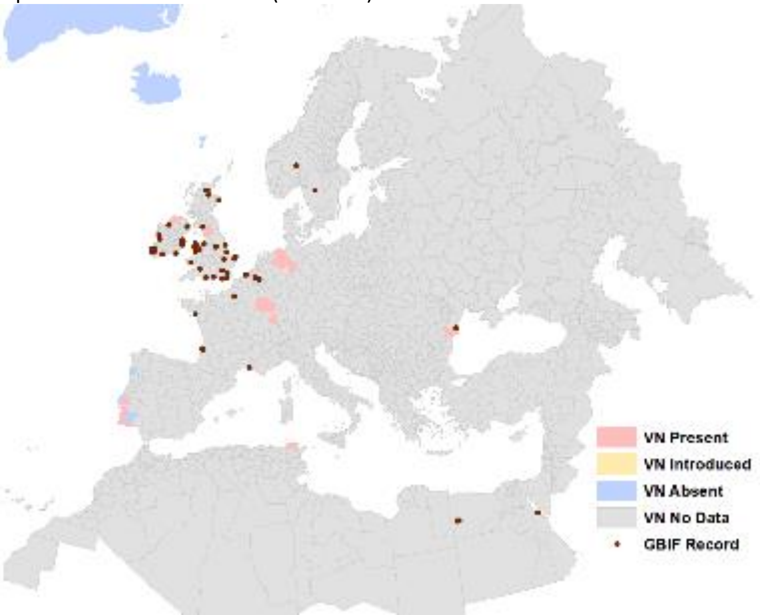

GBIF Present	VN Present	VN Absent	VN No Data	Total
**No**	12	31	1432	**1475**
**Yes**	1	0	30	**31**
**Total**	**13**	**31**	**1462**	**1506**

There are significant numbers of GBIF records, largely in the UK and Ireland which probably reflect an underreporting of VectorNet *(e.g.* UK Egypt, Ireland) as well as some are questionable (*e.g.*. Norway, Sweden).

Supplementary Figure S14: *Aedes vexans s.l,*

Aedes vexans is mapped as *sensu lato* reflecting the presence of cryptic species in Scandinavia, and lack of documented molecular differentiation of *Ae. vexans vexans* and *Ae. vexans arabiensis* in North Africa and the Middle East. It is the primary European floodwater mosquito, which can generate huge nuisance along flood plains. It develops in temporary freshwater bodies such as flooded meadows, poplar cultures, willow and reed areas (Becker et al., 2010). This pest species is targeted by control programmes in many areas (e.g. Danube, Rhine, Rhone floodplains) (Schaffner et al., 2001). *Aedes vexans* has many attributes of an ideal vector species; it was found naturally infected by several encephalitis viruses and is involved in the transmission of Rift Valley fever and Tahyna viruses (cf Becker et al., 2010,. DOI: 10.1007/978-3-540-92874-4).

VN MAP MARCH 21

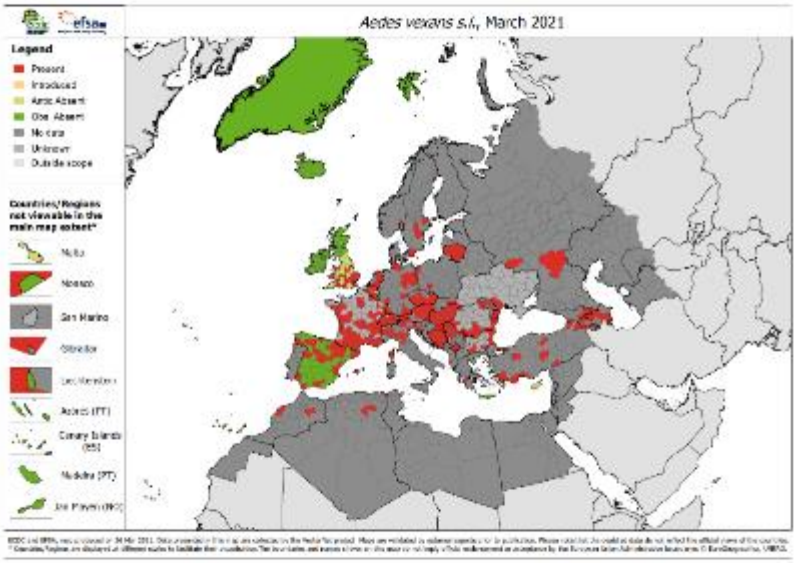

Aedes vexans shows a wide distribution in Europe, but a more restricted one in North Africa and Near East. For some countries, its distribution is underestimated on VectorNet maps, because of the lack of recent presence reports (since 1980) in areas where the species is well known to occur. The species is absent from northern latitudes. Few true absences are reported.

Comparison with GBIF records (in Brown)

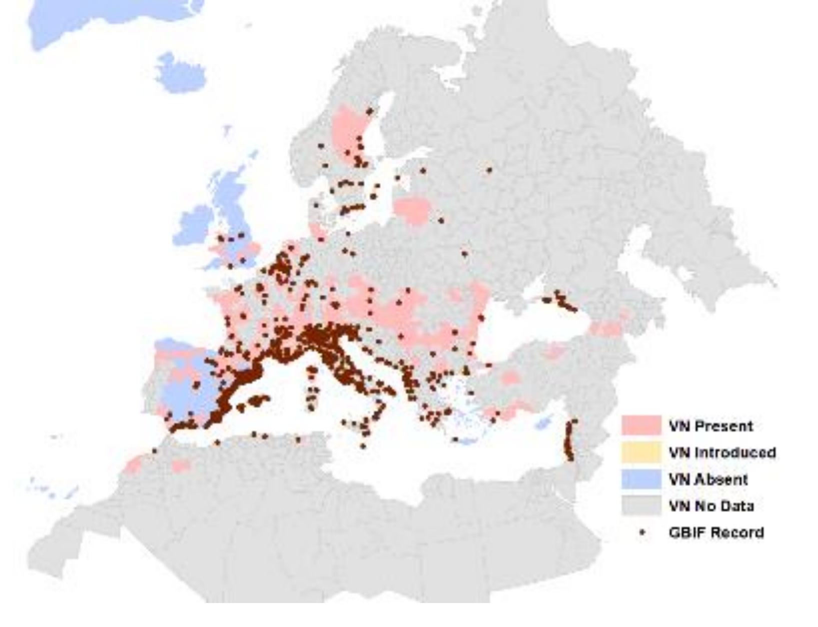

GBIF Present	VN Present	VN Absent	VN No Data	Total
**No**	132	149	915	**1196**
**Yes**	85	21	204	**310**
**Total**	**217**	**170**	**1119**	**1506**

The numerous GBIF records confirm the wide distribution. They also suggest a lack of VectorNet data reports in southern European countries, the Near East, as well as in Scandinavia. However, many reports are questionable because of a possible confusion with *Aedes caspius,* particularly *in* coastal areas of Spain, Italy, the Balkans, and Israel.

Supplementary Figure S15: *Anopheles maculipennis* s.l.

Anopheles maculipennis s.l. is a species complex of up to eight species which are difficult to distinguish by morphology. The all develop mainly in semi-permanent or permanent water bodies, as long as the aquatic vegetation allows the larvae to hide from predators. This complex includes several species confirmed as historical (when malaria was endemic to the European subcontinent) significant vectors of malaria (i.e. *An. atroparvus*, *An. labranchiae*, *An. messeae*, *An. sacharovi*), their role being different according to the geographical region considered. Some members of the complex can also be involved in the transmission of Batai, Tahyna and West Nile viruses, canine filariae, tularaemia bacteria and Myxoma virus (cf Becker et al., 2010,. DOI: 10.1007/978-3-540-92874-4)).

VN MAP MARCH 21

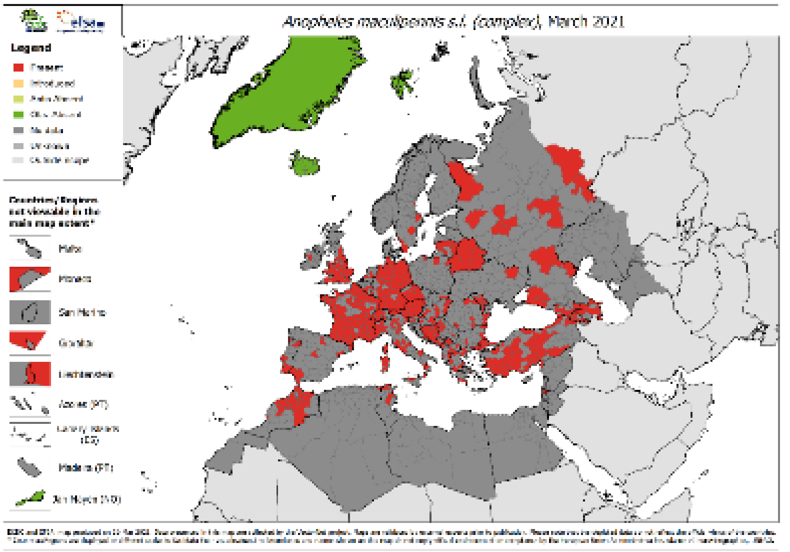

Anopheles maculipennis s.l. is distributed all over Europe, North Africa and Near East. However, the individual members of the complex have more restricted distributions (e.g. *An. atroparvus* is found throughout the Mediterranean Basin, *An. labranchiae* in North Africa, *An. messeae* in central and northern Europe, and *An. sacharovi* in south-eastern Europe). These species can only be identified with certainty based on molecular methods, which are to date not numerous enough to show relevant distribution maps at species level.

Comparison with VectorBase records (in Brown)

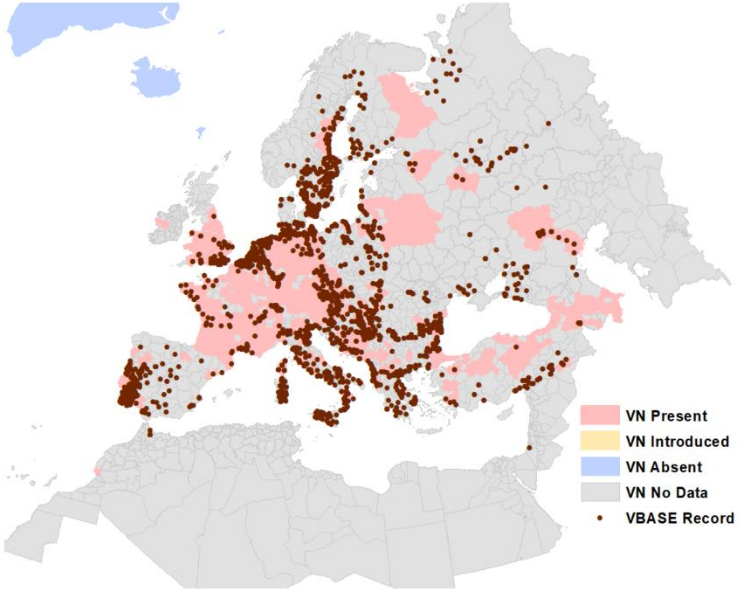

VBASE Present	VN Present	VN Absent	VN No Data	Total
**No**	190	27	813	**1030**
**Yes**	142	0	334	**476**
**Total**	**332**	**27**	**1147**	**1506**

Whilst the majority of VectorBase records match the VectorNet presence polygons, there are many VectorBase records in areas where VectorNet has yet to acquire information, especially in Spain, Turkey Greece, the Balkans, Russia and Scandinavia.

Supplementary Figure S16: *Anopheles plumbeus*

Anopheles plumbeus is a container-breeding species, developing mainly in rainwater-filled tree holes. It occasionally proliferates in unused cesspits (filed by rain water and animal dung residue) generating severe but localised nuisance. The species does not (yet) play an important vector role in Europe as it is highly competent for *Plasmodium falciparum*, and this malaria pathogen is rare in Europe (cf Becker et al., 2010,. DOI: 10.1007/978-3-540-92874-4).

VN MAP MARCH 21

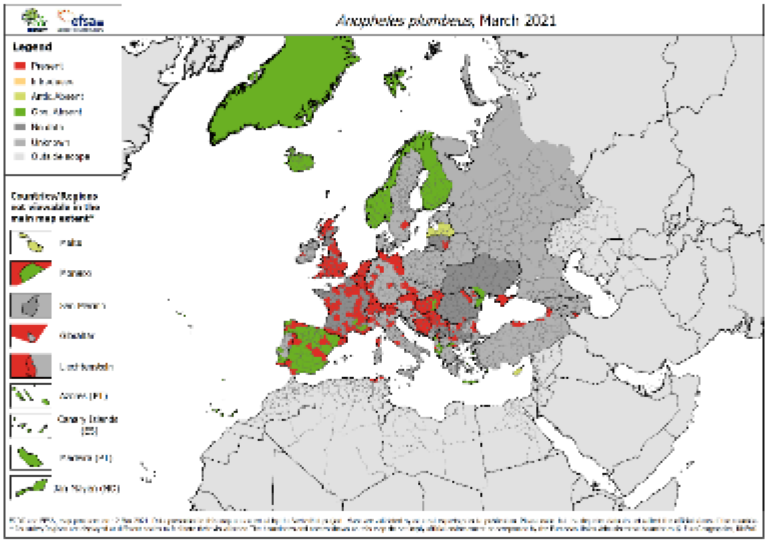

Anopheles plumbeus is widely distributed throughout the European subcontinent, and most probably under-reported. It is present in southern Sweden but absent from more northern latitudes, scarce in North Africa and absent from Near East.

Comparison with GBIF records (in Brown)

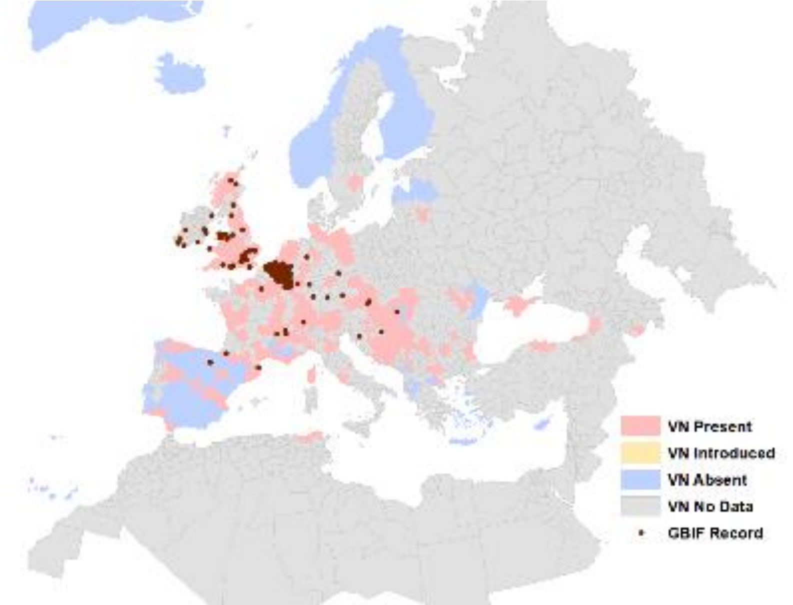

GBIF Present	VN Present	VN Absent	VN No Data	Total
No	199	151	1100	**1450**
Yes	36	1	19	**56**
**Total**	**235**	**152**	**1119**	**1506**

GBIF records are not numerous and globally fit the VectorNet data set or are within the distributions inferred by current VectorNet data (Ireland, Germany). The abundance of reports for Belgium is the consequence of a single mapping project but the inferred distribution does not contradict published data.

Supplementary Figure S17: *Anopheles superpictus*

Anopheles superpictus breeds in slow running or standing water in river beds or in irrigated rice fields. The species is reported as being an important vector of malaria in Middle East and a secondary vector in other regions where it occurs; it may also play a role in the transmission of *Dirofilaria immitis* (cf Becker et al., 2010,. DOI: 10.1007/978-3-540-92874-4).

VN MAP MARCH 21

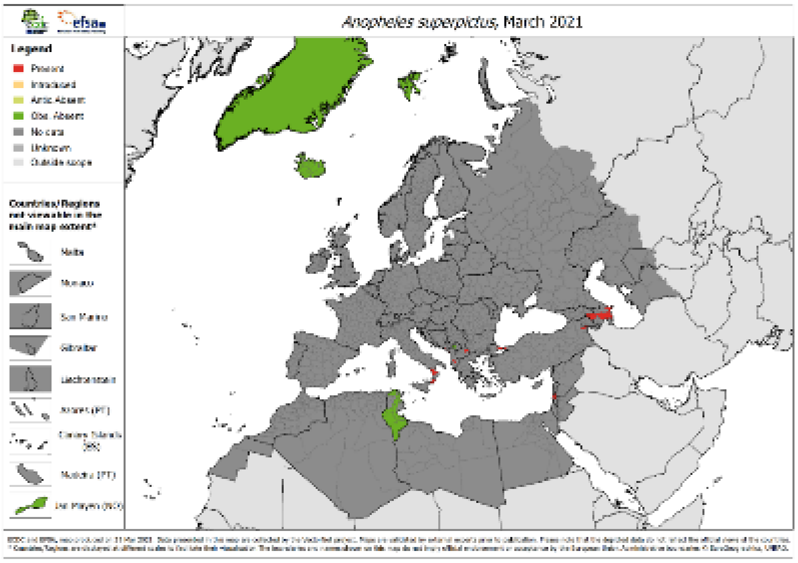

The reported VectorNet data confirm that the species distribution is restricted to the eastern Mediterranean Basin, Turkey and the Caucasus. Anticipated absence data may be inferred for eastern-Mediterranean and northern regions.

Comparison with VectorBase records (in Brown)

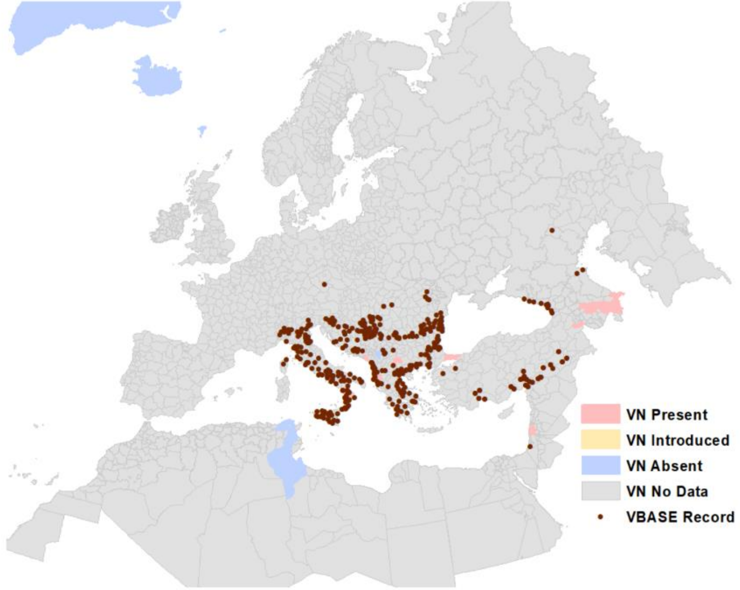

VBASE Present	VN Present	VN Absent	VN No Data	Total
**No**	10	43	1270	**1323**
**Yes**	2	0	181	**183**
**Total**	**12**	**43**	**1451**	**1506**

The VectorBase data base has considerably more records than does the VectorNet archive, indicating that VectorNet lacks at least one major source of information (either literature or sample programme) for this species.

Supplementary Figure S18: *Coquillettidia richiardii*

Coquillettidia richiardii breeds in permanent water bodies with erected aquatic vegetation such as reeds and bulrush (*Typha* sp.). Larvae and pupae live submerged and obtain oxygen from the aerenchym of aquatic plants and move very little. Adults can be very numerous, generating a severe nuisance to humans and domestic animals within short distance from their breeding site. They may be involved in the transmission of several pathogens, including Batai, Tahyna and West Nile viruses (cf Becker et al., 2010,. DOI: 10.1007/978-3-540-92874-4).

VN MAP MARCH 21

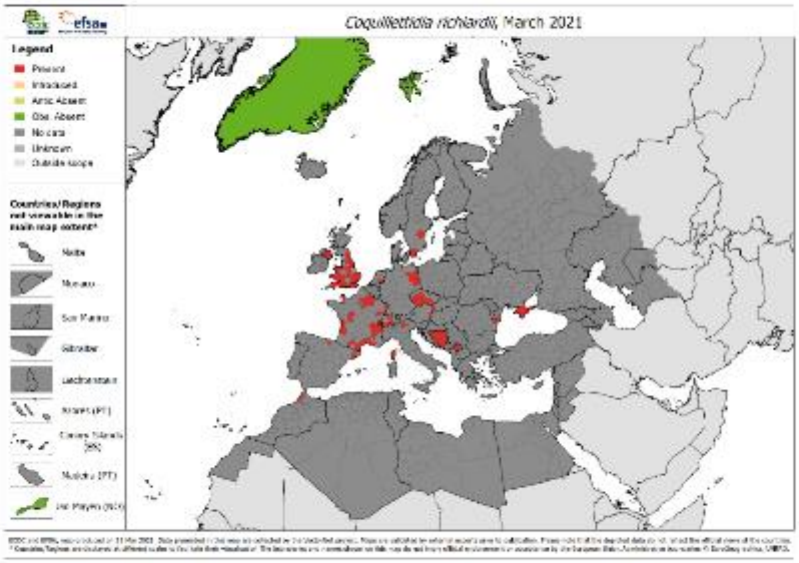

Coquillettidia richiardii shows a wide but patchy distribution. It is present in southern Sweden but absent from more northern latitudes and from Near East, and scarce in North Africa. Very few confirmed absences are reported, and anticipated absences remain to be reported (e.g. northern latitudes).

Comparison with GBIF records (in Brown)

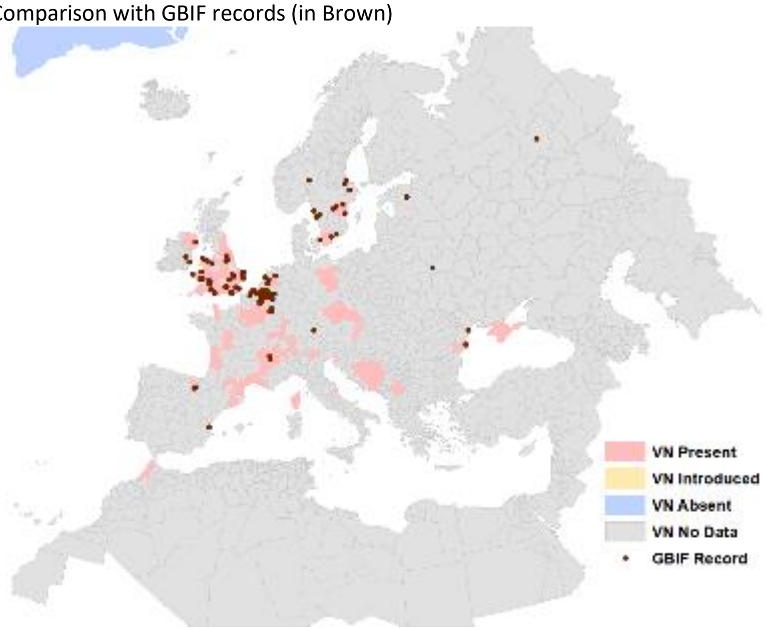

GBIF Present	VN Present	VN Absent	VN No Data	Total
**No**	67	3	1387	**1457**
**Yes**	16	0	33	**49**
**Total**	**83**	**3**	**1420**	**1506**

GBIF records are found in some of the polygons that VectorNet defines as present, but also in many for which VectorNet has no data. Most of these are reasonably near the range implied by the VectorNet records (Sweden, Spain, Belgium) though there do appear to be some more unlikely outliers (Russia, Norway)

Supplementary Figure S19: *Culex modestus*

Culex modestus is a wetland mosquito that breeds mainly in rice fields, irrigation canals and semi-permanent marshes (Becker et al., 2010). this species sometimes generates significant nuisance for human and other mammals in the immediate neighbourhood of breeding places. *Culex modestus* is considered to be an important vector of West Nile virus in some places (e.g. Camargue, southern France) and is involved in the transmission of Tahyna and Myxoma viruses. Moreover, wild females have been found infected by tularaemia bacteria, Lednice and Sindbis viruses, and *Dirofilaria immitis*.

VN MAP MARCH 21

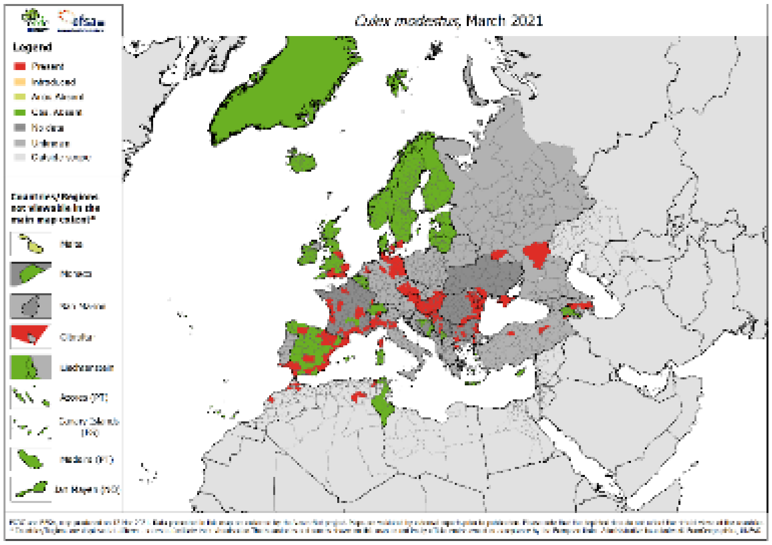

Culex modestus is widely distributed over the European continent. It is absent from northern latitudes and is scarce in Near East and North Africa. During the last decade, the species has become more abundant in UK, and was found for the first time in Belgium, Denmark and southern Sweden.

Comparison with GBIF records (in Brown)

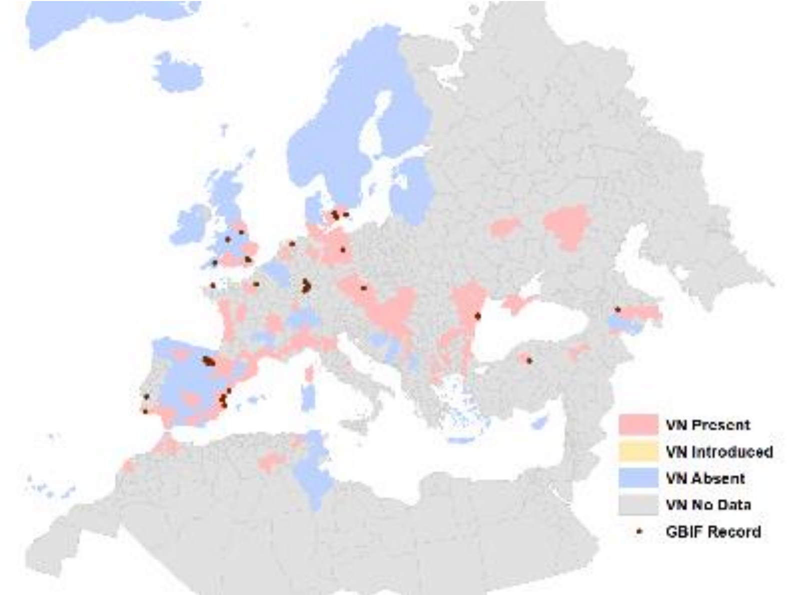

GBIF Present	VN Present	VN Absent	VN No Data	Total
No	153	372	958	**1483**
Yes	11	4	8	**23**
**Total**	**164**	**376**	**966**	**1506**

Few GBIF records exist; they mostly fit with VectorNet data or are recorded close to the implied range, which are feasible but need to be validated by existing scientific publication or data set (e.g. southern Germany). Some GBIF records may be considered doubtful (northern France, UK).

Supplementary Figure S20: *Culex pipiens*: Group

The *Culex pipiens* group comprises the Pipiens species complex and the sibling species *Cx. torrentium* and has a global distribution. In Europe, the Near East and North Africa, the Pipiens complex is represented by *Cx. pipiens*, form *pipiens*, which develops in the wild in clear water, bites mainly birds and diapauses in winter as an adult; and the form *molestus*, which develops in more polluted underground water throughout the year and bites mainly mammals. Females can be a major nuisance indoors and at night, in particular where underground breeding places exist. *Culex torrentium* is a northern species, common in high altitudes and rare or absent in the Mediterranean Basin. Larvae of both species develop in any man-made container as well as in a wide range of natural standing water bodies (e.g. ponds, ditches, marshes, rock pools, tree holes). *Culex pipiens* is considered an important vector of West Nile virus and avian plasmodia, and both *Cx. pipiens* and *Cx. torrentium* contribute to the transmission of Sindbis virus (cf Becker et al., 2010,. DOI: 10.1007/978-3-540-92874-4)

VN MAP MARCH 21

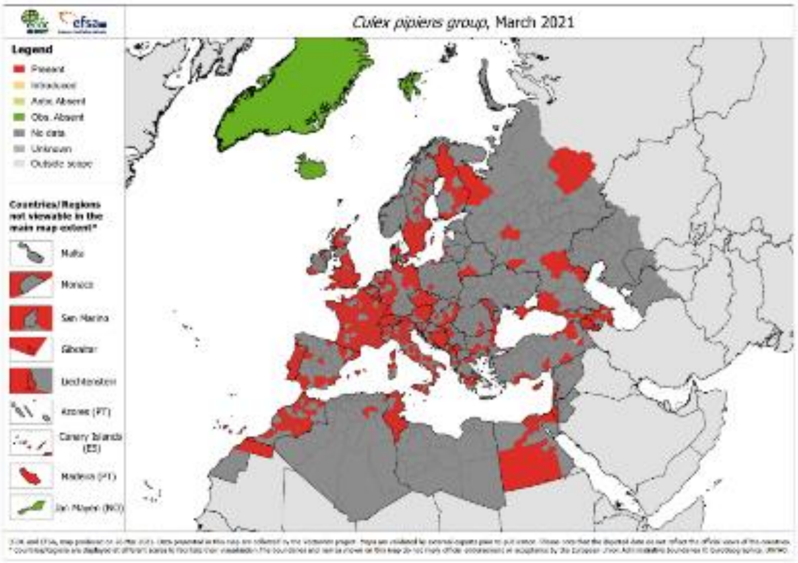

The Pipiens complex is widely distributed throughout the European subcontinent, the Near East and North Africa. Little true absence data exist and the species might be present in almost all dark grey (no data) areas, since potential breeding sites exist everywhere (at least around human settlements). However, in high altitudes and northern latitudes, the species might be replaced by *Cx. torrentium*, and part of presence reports in these areas might be wrongly attributed to *Cx. pipiens*

Comparison with GBIF records (in Brown)

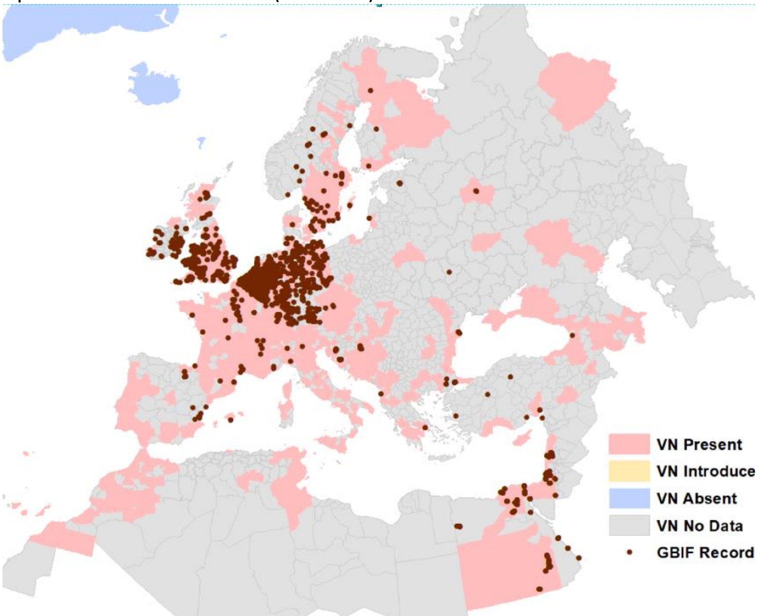

GBIF Present	VN Present	VN Absent	VN No Data	Total
No	418	27	851	**1296**
Yes	107	0	103	**210**
**Total**	**525**	**27**	**954**	**1506**

About half of the GBIF records are located in VectorNet polygons with no data, in particular in Benelux, Germany and Ireland. To address that, an effort in accessing grey literature and unpublished data sets for these countries is required. There are no GBIF records in VectorNet polygons defined as absent.

For this species in particular, VectorNet data are under-reported considering the reported surveillance effort (Figure S2).

Supplementary Figure S21: *Culicoides chiopterus*

Culicoides chiopterus is a species of the subgenus *Avaritia*, with a Holarctic distribution. This species has been identified in the Palearctic zone from Europe to Japan. Immatures of *C. chiopterus* develop exclusively in cattle and horse dung. Adults have been recorded feeding on a variety of mammals including ruminants, equids, suids, rabbits and humans. *Culicoides chiopterus* has been implicated in the transmission of veterinary pathogens including bluetongue and Schmallenberg viruses

VN MAP MARCH 21

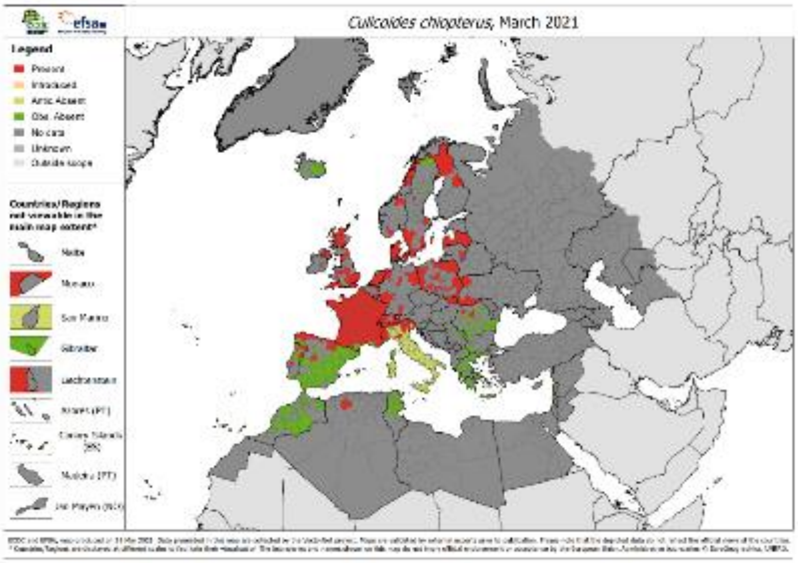

Culicoides chiopterus has a wide distribution in Europe, with a clear North to South gradient of abundance. Indeed, *C. chiopterus* may be abundant in cattle farms, due to its larval habitat requirement, in Northern Europe, but becomes rare to absent in southern Europe. There is a single record of this species in northern Africa, in the High Plains of Algeria, suggesting the presence of this species in limited environments.

Comparison with GBIF records (in Brown)

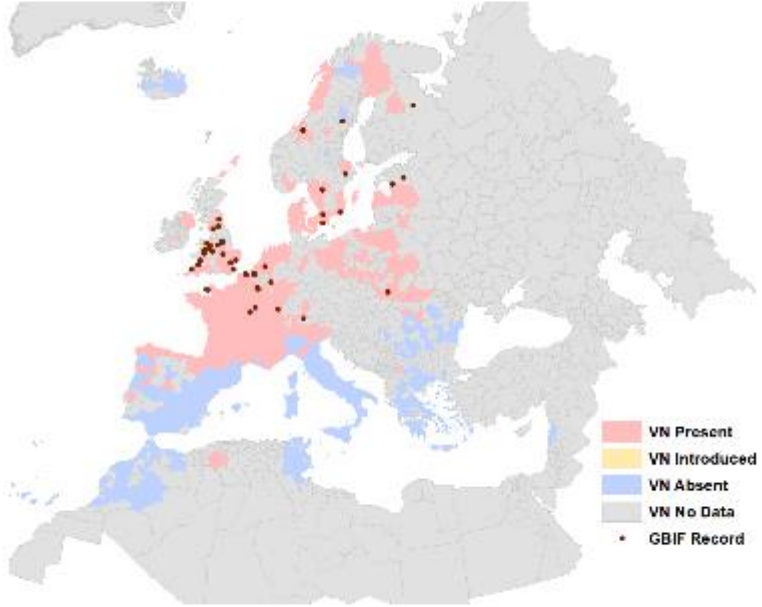

GBIF Present	VN Present	VN Absent	VN No Data	Total
No	211	238	1023	**1472**
Yes	23	0	11	**34**
**Total**	**234**	**238**	**1034**	**1506**

GBIF records of *C. chiopterus* are located in the northern part of Europe, where this species is the most abundant. Two thirds of them fall in the VectorNet polygons of presence, whereas a third fall in the VectorNet polygons with no data, especially in the United Kingdom and in Estonia, highlighting possible unpublished datasets in these countries.

Supplementary Figure S22: *Culicoides dewulfi*

Culicoides dewulfi is a species belonging to the subgenus *Avaritia*, with a Palearctic distribution, including northern Russia. This species has a biology and an ecology very similar to *C. chiopterus*. Indeed immatures develop exclusively in cattle and horse dung. Moreover, this species has been recorded feed on a variety of mammals, including ruminants, equids or also humans. *Culicoides dewulfi* has been implicated in the transmission of bluetongue and Schmallenberg viruses in Europe.

VN MAP MARCH 21

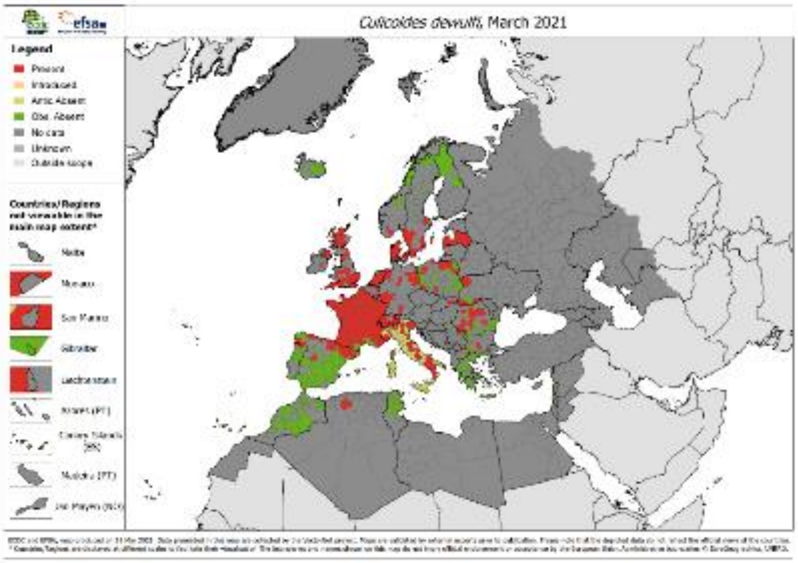

Culicoides dewulfi has, as *C. chiopterus*, a wide distribution in Europe. However, even if they are both dung-breeding species, the northern limit of *C. dewulfi* is at a more southern latitude than *C chiopterus*, as this species is absent from northern Scandinavia, whereas its southern limit is at a more southern latitude, as this species is recorded in southern Italy. There is a single record of this species in northern Africa, in the High Plains of Algeria, suggesting the presence of this species in particular environments.

Comparison with GBIF records (in Brown)

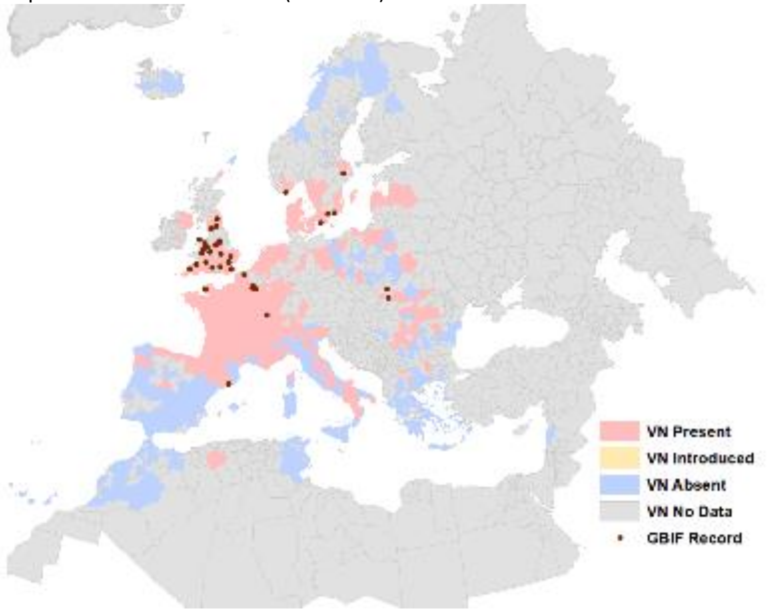

GBIF Present	VN Present	VN Absent	VN No Data	Total
**No**	217	232	1028	**1477**
**Yes**	21	0	8	**29**
**Total**	**238**	**232**	**1036**	**1506**

The GBIF records for *C. dewulfi* are located mostly in the northern part of Europe, where this species is the most abundant. Three quarters of them fall in the VectorNet polygons of presence, whereas few fall in the VectorNet polygons with no data, especially in the United Kingdom, highlighting possible unpublished datasets in this country.

Supplementary Figure S23*: Culicoides imicola*

Culicoides imicola is a species of the *Avaritia* subgenus. This Afrotropical species is widespread in Africa, in the Mediterranean basin and in the Middle East, and is occasionally recorded in the Far East (*e.g.* India). Immatures develop in a wide range of semi-aquatic habitats that are usually associated with livestock productions, in areas next to pond shorelines and irrigation channels. This adult feeds opportunistically on a wide variety of mammals including ruminants, equids, humans (rarely) and exotic host species in zoos. *Culicoides imicola* is a well-known vector of economically important livestock viruses such as bluetongue virus affecting domestic and wild ruminants or African horse sickness (AHS) virus affecting equids. The ability of culicoides to transmit these orbiviruses was first demonstrated in this species in the 1940s.

VN MAP MARCH 21

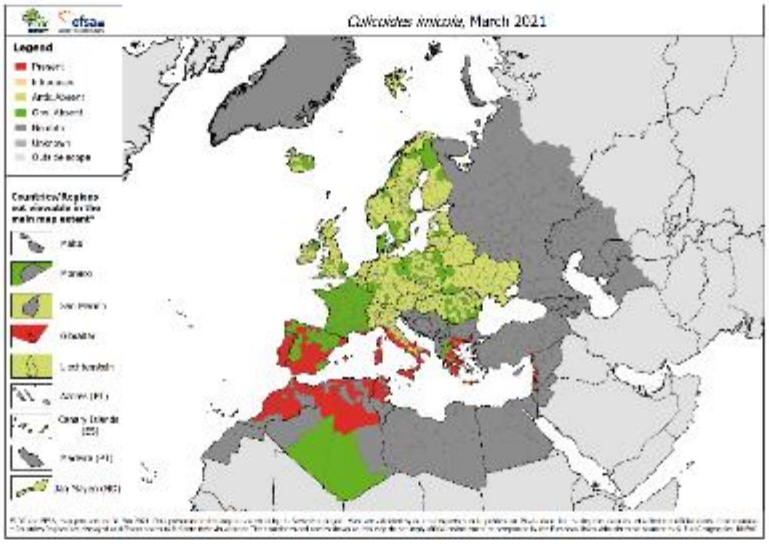

Culicoides imicola is widespread all around the Mediterranean Sea, including western Mediterranean islands where this species is particularly abundant. Its northern distribution limit borders the North of Portugal, crosses Spain – this species is absent in the Extremadura region, but recorded in northwestern and northeastern Spain, touches the South of the French mainland, follows the Italian Ligurian and Tyrrhenian coast and touches the southernmost part of North Macedonia and Bulgaria. The southern limit highlights the transition between the Mediterranean area and the Saharan one This species is better studied than most of the rest of the genus, and the absences better researched.

Comparison with GBIF records (in Brown)

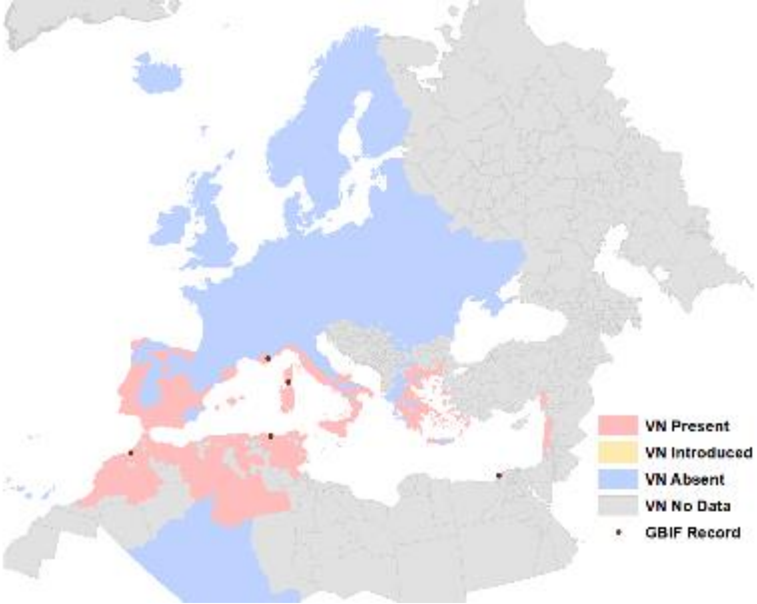

GBIF Present	VN Present	VN Absent	VN No Data	Total
**No**	216	738	546	**1500**
**Yes**	4	0	2	**6**
**Total**	**220**	**738**	**548**	**1506**

Given the epidemiological prominence of this species, it is perhaps surprising that there are only 6 GBIF records for *C. imicola.* This may reflect the academic value of data on this species, which often hinders publishing data to the public domain. The 2 records falling outside VectorNet presence polygons are in Egypt, from where this species is known. No information is available up to now in the VectorNet database for this country.

Supplementary Figure S24: *Culicoides kingi*

Culicoides kingi belongs to the Schultzei group, which is affiliated to the Remmia or the Wirthomyia subgenera depending on author. This species has an Afrotropical distribution, with records in Africa, in the Sahel and the Maghreb. Immatures have been observed to emerge from the edge of lakes or ponds in Senegal, whereas adults attack livestock such as horses or ruminants. The virus of the epizootic hemorrhagic disease has been isolated from *C. kingi* in Sudan and Nigeria (cf Mellor, p. *et al*. doi: 10.1017/s0022172400065190.) This species is also associated with the transmission of *Onchocerca gutturosa* in cattle.

VN MAP MARCH 21

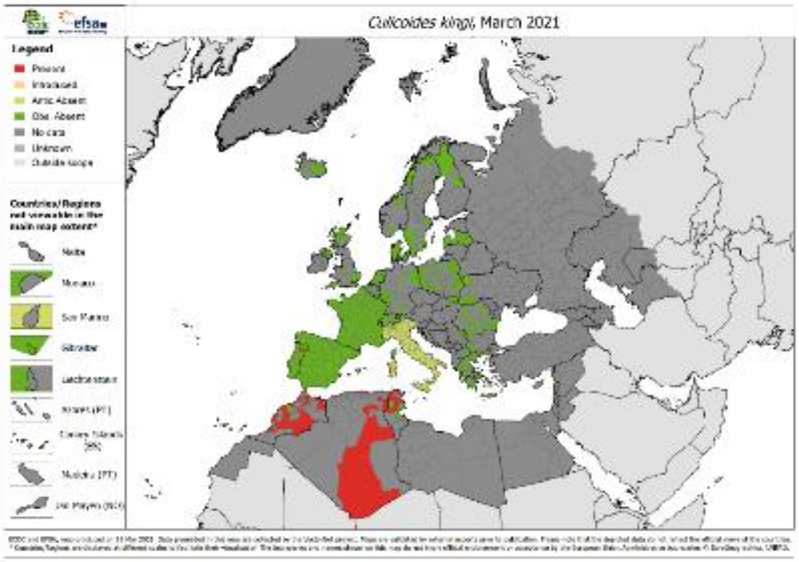

Culicoides kingi is abundant in northern Africa, in the Mediterranean areas, but also in the arid Sub-Saharan regions. This species is becoming very rare in the northern part of the Mediterranean basin, but not completely absent as highlighting by sporadic records.

Comparison with GBIF records (in Brown)

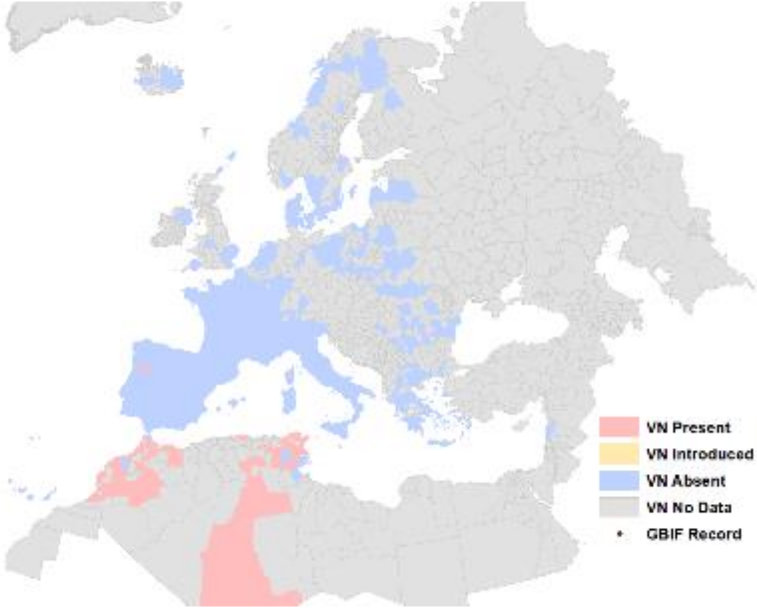

There is no GBIF records for *C.kingi*.

GBIF Present	GBIF Present	VN Present	VN Absent	VN No Data	Total
**No**	41	435	1030	**1506**	41
**Yes**	0	0	0	**0**	0
**Total**	**41**	**435**	**1030**	**1506**	**41**

Supplementary Figure S25: *Culicoides newsteadi*

Culicoides newsteadi s.l. belongs to the *Culicoides* subgenus. Cryptic diversity has been suggested within this taxon by molecular investigations (cf This https://doi.org/10.1111/j.1365-2915.2012.01050.x) species has a Palearctic distribution. Immatures are associated with brackish marshes, and adults have been recorded feed on the different domestic ruminants (cattle, goats, sheep), but also on humans, and sporadically on domestic birds. *Culicoides newsteadi s.l.* is considered a possible bluetongue and Schmallenberg viruses vector, due to its ecological habits and to virus isolation/viral genome detections from field-collected individuals in Mediterranean regions.

VN MAP MARCH 21

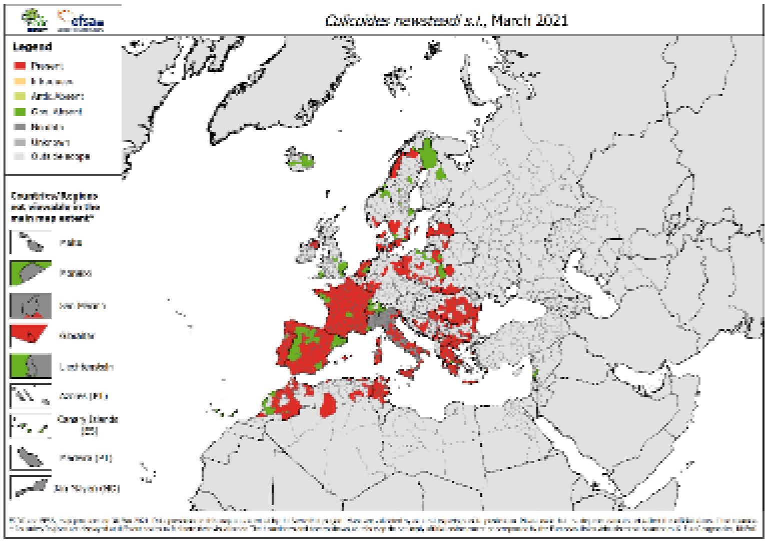

Culicoides newsteadi s.l. is widespread in Europe and in the Mediterranean basin. This species is absent from Iceland, and rare in Scandinavia. Elsewhere, its sparse distribution may be induced by the repartition of larval habitats. *Culicoides newsteadi s.l.* may be very abundant, even dominant, in some Mediterranean regions.

Comparison with GBIF records (in Brown)

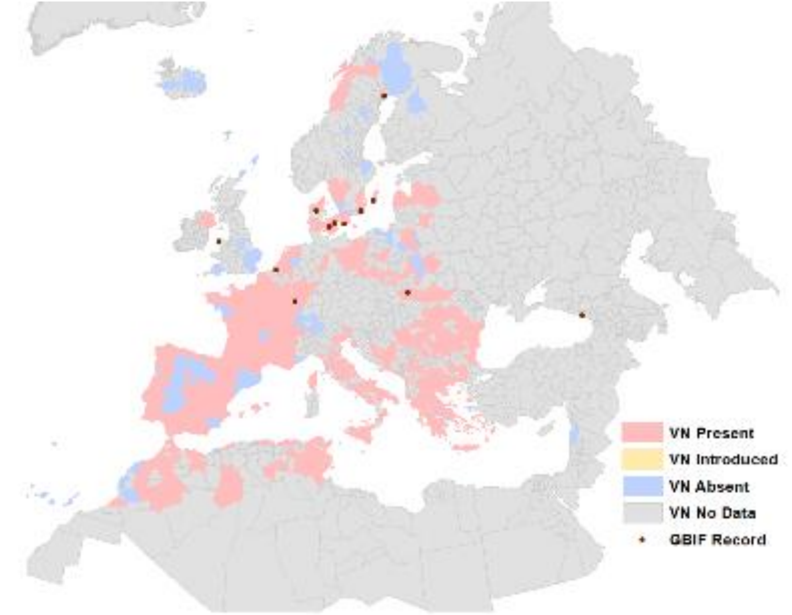

GBIF Present	VN Present	VN Absent	VN No Data	Total
No	396	75	1025	**1496**
Yes	6	0	4	**10**
**Total**	**402**	**75**	**1029**	**1506**

There are only few GBIF records for *C. newsteadi s.l.* Most of them fall just outside within the VectorNet presence polygons.

Supplementary Figure S26: *Culicoides pulicaris s.l./Culicoides lupicaris*

The taxon *Culicoides pulicaris s.l./Culicoides lupicaris* includes species of the subgenus *Culicoides*, namely *C. pulicaris s.l.* that may include cryptic diversity (cf https://doi.org/10.1111/j.1365-2915.2012.01050.x) and *C. lupicaris*. Some authors consider *C. lupicaris* as a synonym of *Culicoides delta* although evidences suggest two distinct valid species (cf https://www.inhs.illinois.edu/files/5014/6532/8290/CulicoidesSubgenera.pdf) . These species are grouped because diagnostic wing patterns cannot be distinguished. These species have a Palearctic distribution. Immatures have been found at the edges of ponds, whereas adults have been recorded feeding on ruminants (cattle and deer) and on horses. *Culicoides pulicaris s.l.* is considered a bluetongue and Schmallenberg viruses vector.

VN MAP MARCH 21

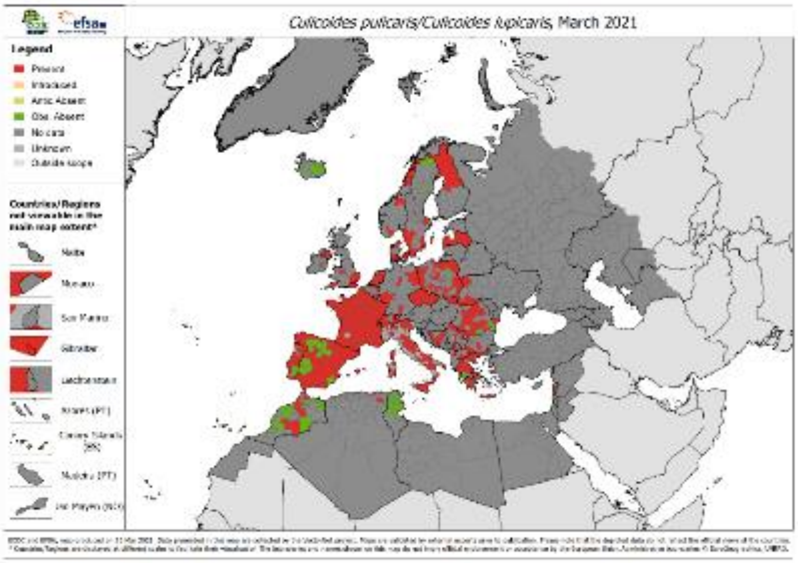

Culicoides pulicaris s.l./C. lupicaris has a wide distribution in Europe, but is absent from Iceland. Abundances become low in Mediterranean regions, whereas the taxon is rare in Northern Africa.

Comparison with GBIF records (in Brown)

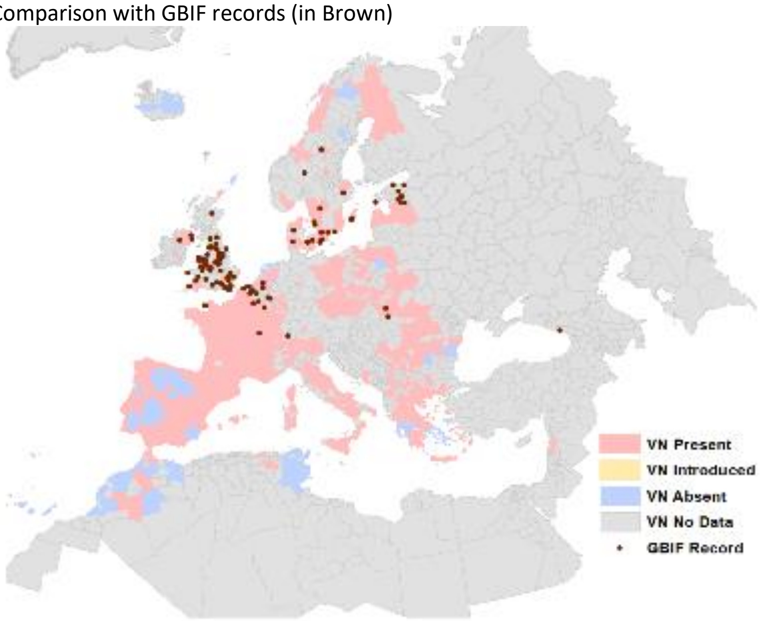

GBIF Present	VN Present	VN Absent	VN No Data	Total
**No**	360	71	1023	**1454**
**Yes**	10	0	42	**52**
**Total**	**370**	**71**	**1065**	**1506**

GBIF records of *C. pulicaris s.l.* and *C. lupicaris* are mostly located in the northern part of Europe. Twenty percent of them fall in the VectorNet polygons of presence, whereas the rest fall in the VectorNet polygons with no data, located mainly in the United Kingdom, in Belgium and in Estonia, where there are only sporadic VectorNet records, thus highlighting possible unpublished datasets in these countries.

Supplementary Figure S27: *Culicoides punctatus*

Culicoides punctatus s.l. belongs to the subgenus *Culicoides*. New species have been proposed after morphological detailed and genetic investigations of individuals firstly identified as *C. punctatus* (cf https://onlinelibrary.wiley.com/doi/full/10.1111/mve.12228) . This species has a Palearctic distribution. Immatures have been found at the edges of ponds, whereas adults have been recorded feed on a variety of mammals, including cattle, goats, deer and zoo large mammals, equids, suids or humans. *Culicoides punctatus s.l.* is considered a possible bluetongue and Schmallenberg viruses vector, due to its ecological habits and to virus isolation/viral genome detections from field-collected individuals.

VN MAP MARCH 21

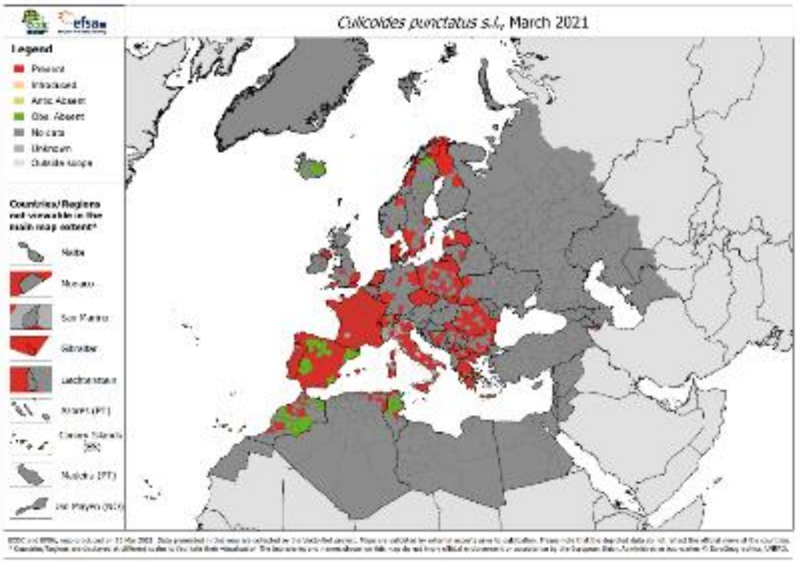

Culicoides punctatus s.l. is widely distributed in Europe, becoming rare in Northern Africa. This species is absent from Iceland, whereas is becoming abundant in north-eastern Europe.

Comparison with GBIF records (in Brown)

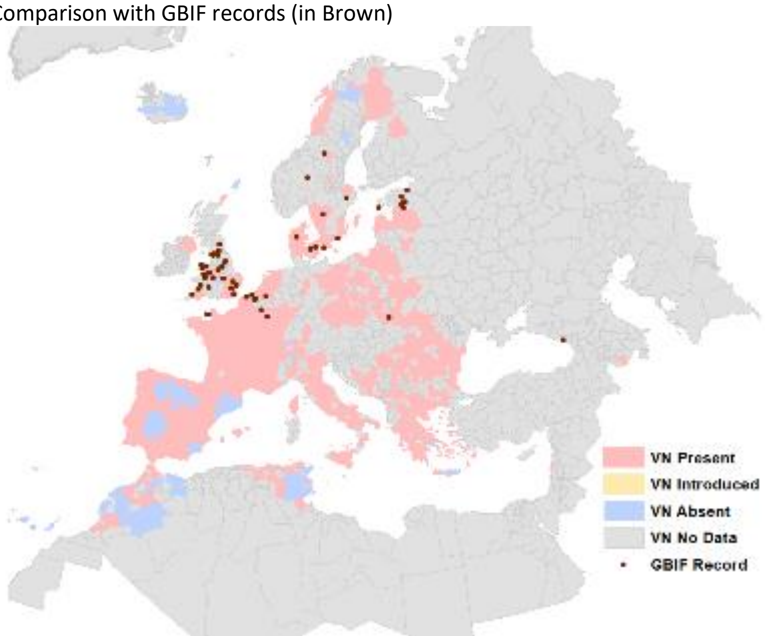

GBIF Present	VN Present	VN Absent	VN No Data	Total
**No**	438	58	973	**1469**
**Yes**	15	0	22	**37**
**Total**	**453**	**58**	**995**	**1506**

GBIF records of *C. punctatus s.l.* are mostly located in the northern-western part of Europe. Forty percent of them fall in the VectorNet polygons of presence, whereas the rest, falling in the VectorNet polygons with no data. These are located mainly in the United Kingdom, in Belgium and in Estonia, where there are only sporadic VectorNet records, highlighting possible unpublished datasets in these countries.

Supplementary Figure S28: *Culicoides obsoletus s.l./Culicoides scoticus*

The taxon *Culicoides obsoletus s.l./Culicoides scoticus* (cf doi: 10.1186/s13071-020-04114-1) includes species of the subgenus *Avaritia*, namely *C. scoticus* (that may include cryptic diversity), *C. montanus, C. obsoletus s.s.* and few other species that were identified after molecular investigations. The status of *C. montanus* as valid species is still uncertain. *Culicoides scoticus* is almost always grouped with *C. obsoletus s.l.* in routine identification process, because morphological separation of females is uncertain, though males can be separated accurately. Adult females of this taxon feed on a wide variety of mammalian hosts and appear to be opportunistic in preference. Feeding on avian hosts (*e.g*. ducks and chickens) has been recorded for *C. obsoletus s.l.* but is relatively rare. This taxon has been strongly implicated in the transmission of bluetongue and Schmallenberg viruses, and *C. obsoletus s.l.* may have played a role in the transmission of African horse sickness virus.

VN MAP MARCH 21

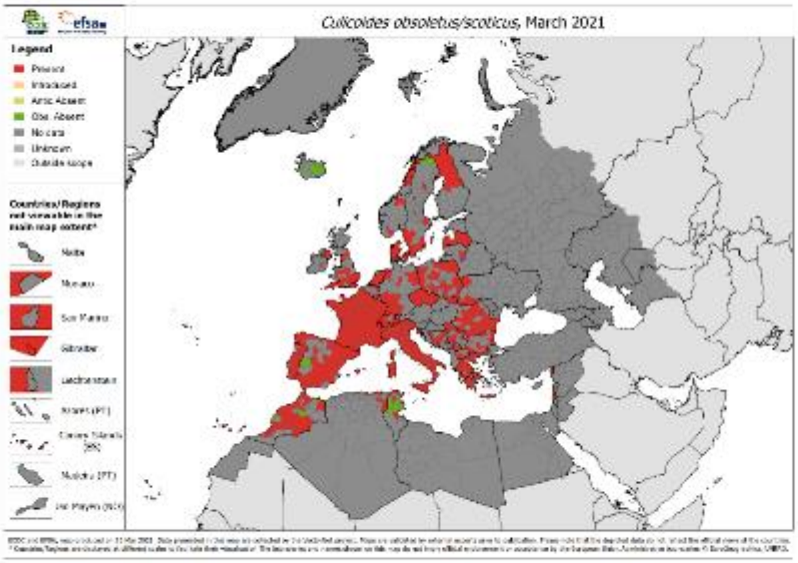

Comparison with GBIF records (in Brown)

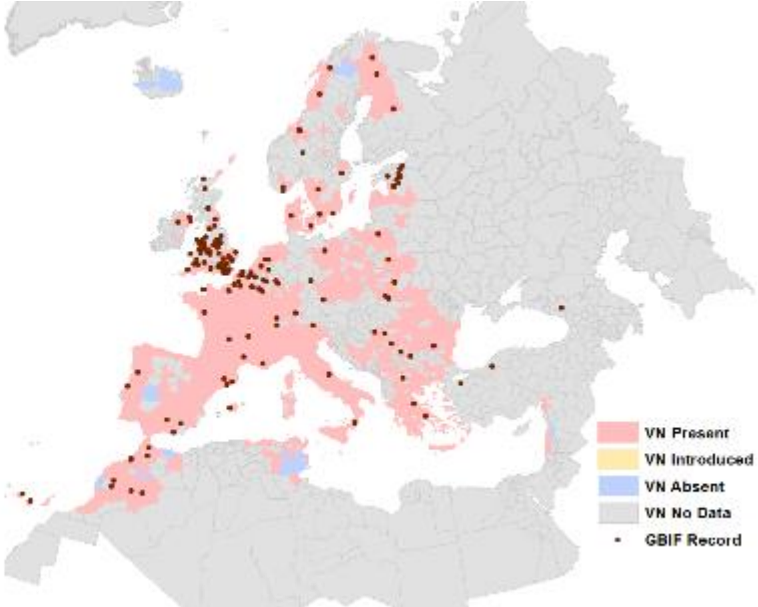

GBIF Present	VN Present	VN Absent	VN No Data	Total
**No**	470	20	904	**1394**
**Yes**	73	0	39	**112**
**Total**	**543**	**20**	**943**	**1506**

The taxon is present virtually everywhere in Europe, except in Iceland. Absence records are located in the Extramadura region in Spain, and occasionally in Maghreb. *C. obsoletus s.l.* is the most abundant species of biting midge in most of northern Europe, and catches of over ten thousand individuals in a single night of light trapping are frequently reported. It has been found at altitudes of up to 1,200 m. Its abundance and cryptic diversity decreases in Mediterranean regions, especially in Northern Africa. . *C. obsoletus s.l.* has a Holarctic distribution, although this status as a single species is under investigation, whereas *C. scoticus* is restricted to the western Palearctic zone. Immature stages of *C. obsoletus s.l./C. scoticus* require a semi-aquatic environment and organic enrichment. Habitats of this taxon identified include deciduous leaf litter, compost heaps, the dung-soil interface, marginal vegetation in open water and silage and also, for *C. scoticus,* tree fungi.

Two thirds of the GBIF records are located in the VectorNet presence polygons. Most of the GBIF records fall in the VectorNet polygons with no data are located in the United-kingdom where VectorNet data are scarce and from Estonia where there is no VectorNet records, suggesting the existence of unpublished datasets in these countries. Interesting GBIF records are observed in Turkey and in the Caucasus, where very little information is yet available in the VectorNet database.

Supplementary Figure S29: *Phlebotomus alexandri* This species is a suspected vector of *Leishmania infantum* and generally found at a low density.

VN MAP MARCH 21

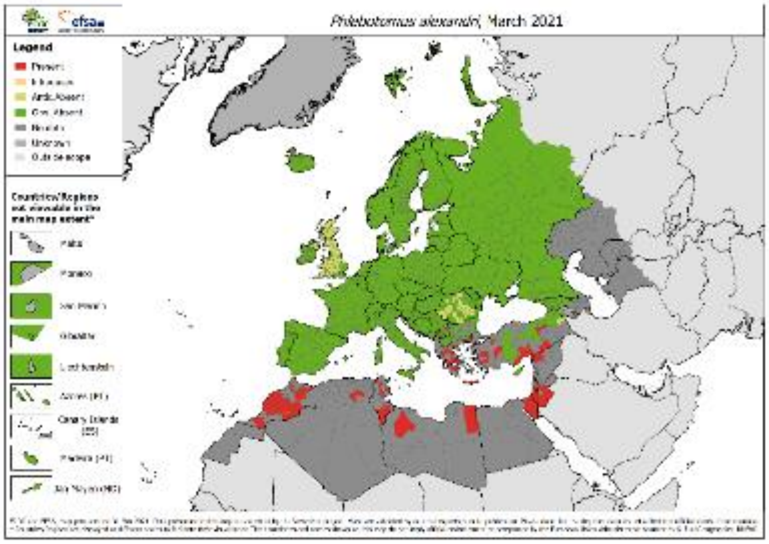

It has been commonly reported from North Africa, The Middle East, Turkey, Cyprus and Greece, and sporadically in Spain, North Macedonia, Serbia, Kosovo and Romania.

Comparison with GBIF records (in Brown)

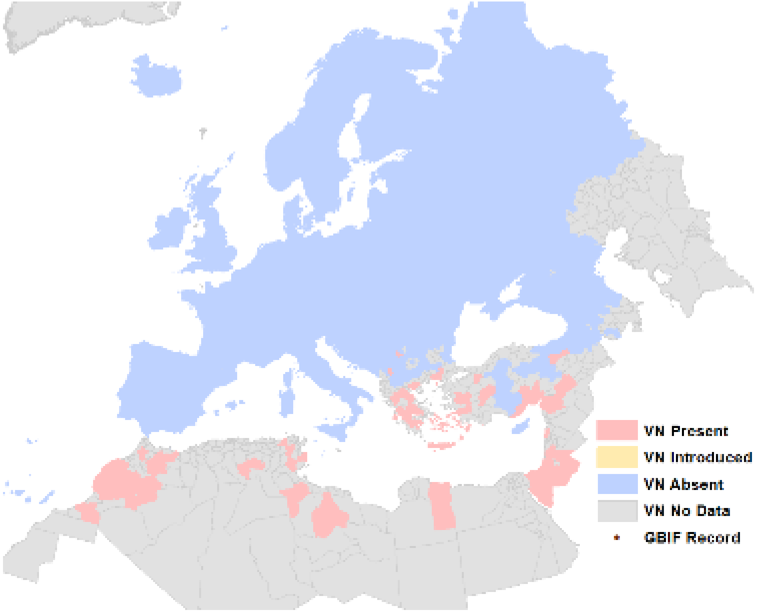

GBIF Present	VN Present	VN Absent	VN No Data	Total
**No**	79	1037	390	**1506**
**Yes**	0	0	0	**0**
**Total**	**79**	**1037**	**390**	**1506**

No GBIF records in VectorNet area

Supplementary Figure S30: *Phlebotomus ariasi* is a confirmed *L. infantum* vector

VN MAP MARCH 21

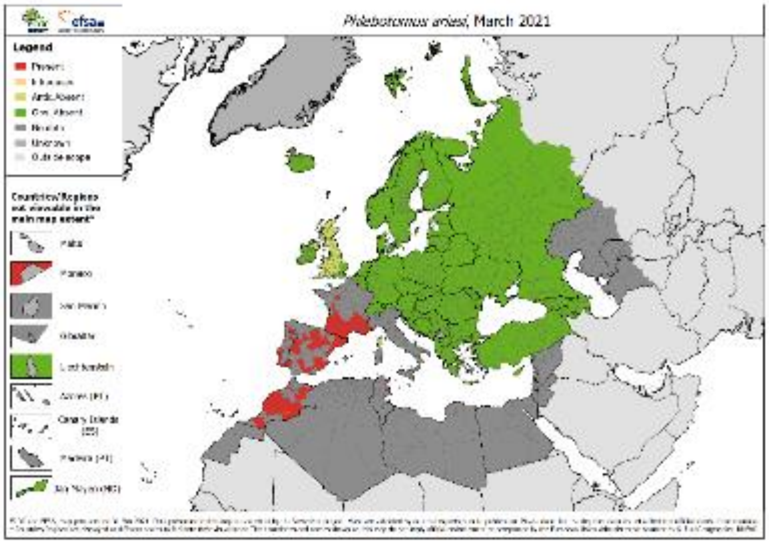

This species has been reported mainly in southern France, Spain, Portugal, Andorra and Morocco, and occasionally from small areas in Italy, Algeria and Tunisia. *P. ariasi* is adapted to cooler climates than its congener *P. perniciosus*, the other main vector in this part of Europe. As a result, its density is much lower than the latter except in high altitude areas in the Pyrenees and the Cévennes mountains in France.

Comparison with GBIF records (in Brown)

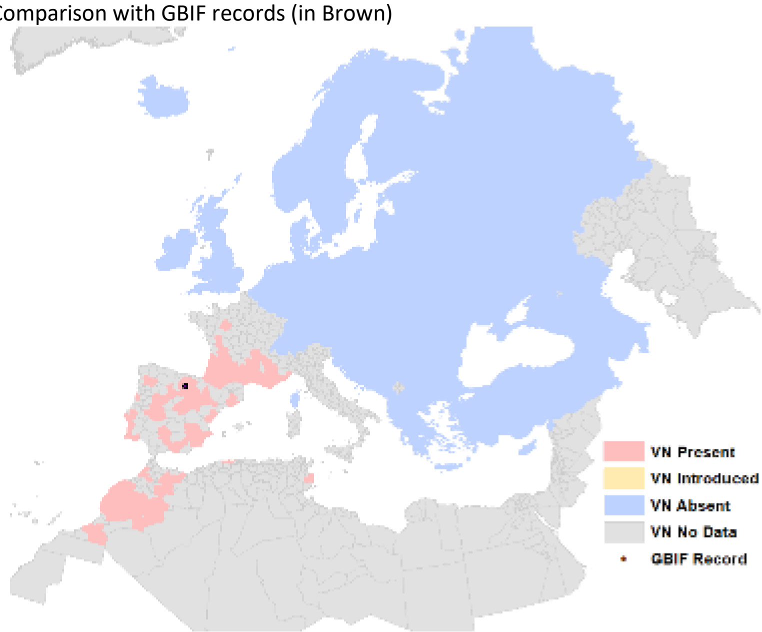

GBIF Present	VN Present	VN Absent	VN No Data	Total
No	77	892	536	**1505**
Yes	1	0	0	**1**
**Total**	**78**	**892**	**536**	**1506**

Only one record of *P. ariasi*, in Spain, is reported in the GBIF map.

Supplementary Figure S31: *Phlebotomus mascittii* is a suspected *L. infantum* vector

VN MAP MARCH 21

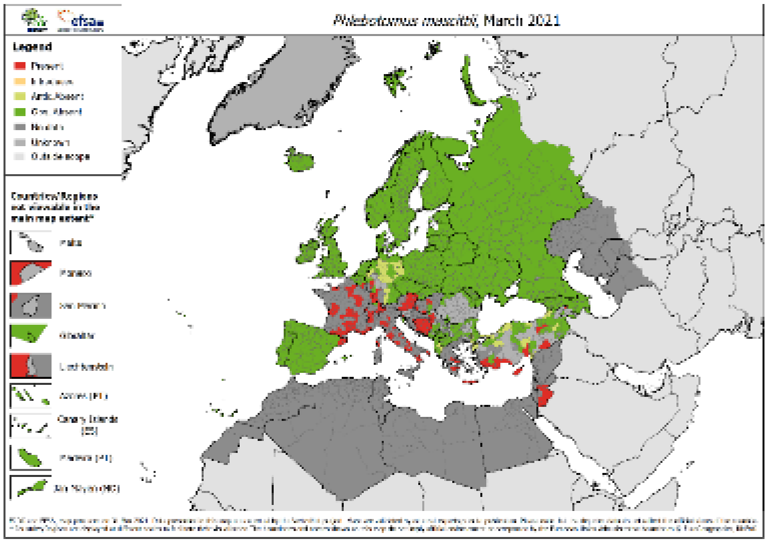

No other sand fly species has been reported as far north in Europe as *P. mascitii*, having been described in Northern France, Belgium, Germany, and Austria. *P. mascitii* spreads all the way from western France and northeast Spain to Turkey in the East. The species has also been reported in the Middle east but not in Northern Africa.

Comparison with GBIF records (in Brown)

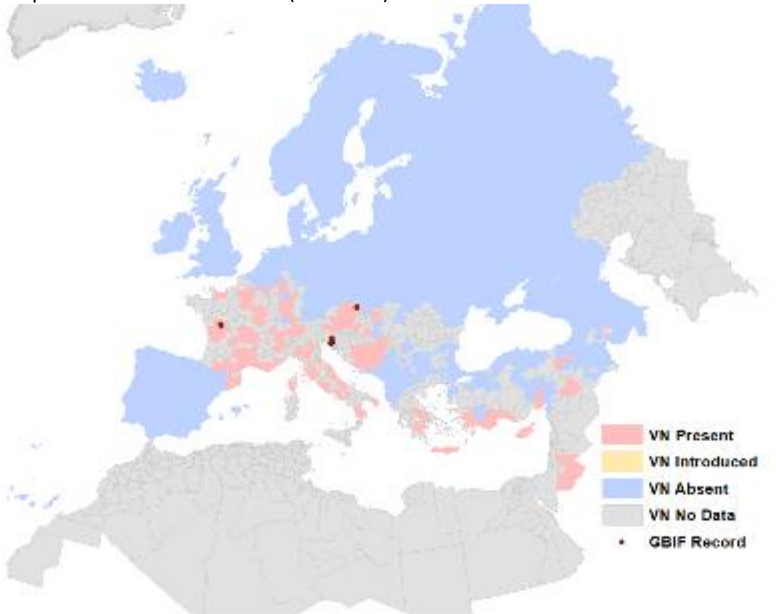

GBIF Present	VN Present	VN Absent	VN No Data	Total
**No**	96	736	670	**1502**
**Yes**	3	0	1	**4**
**Total**	**99**	**736**	**671**	**1506**

This species is reported by GBIF in Western France, as well central Europe and the Balkans, and in an area in where no sand flies of this species have been reported so far.

Supplementary Figure S32: *Phlebotomus neglectus* is a confirmed *L. infantum* vector and is part of the *P. major* s.l. species complex

VN MAP MARCH 21

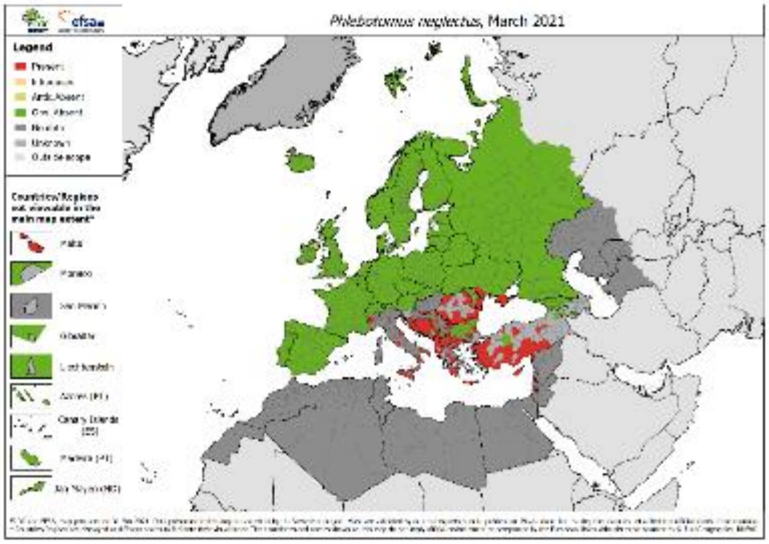

This is the dominant species in the Balkans', Greece and Turkey. Italy and Palestine represent its western and southern limits, respectively. The species has also been reported in southern Germany and Switzerland.

Comparison with Vectorbase records (in Brown)

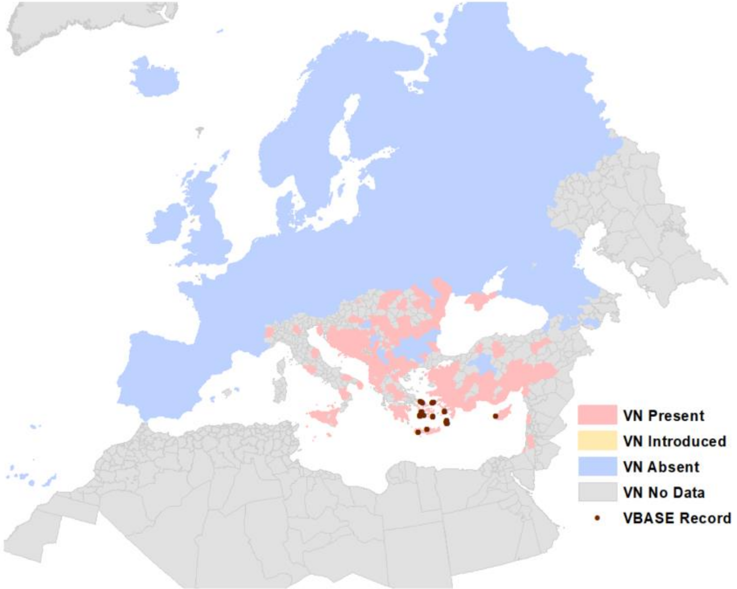

VBASE Present	VN Present	VN Absent	VN No Data	Total
**No**	138	760	602	**1500**
**Yes**	6	0	0	**6**
**Total**	**144**	**760**	**602**	**1506**

The cluster of VectorBase points in Greece match the VectorNet maps, but the VectorNet Map has a much wider range of information.

Supplementary Figure S33: ***Phlebotomus papatasi*** is the confirmed vector of *Leishmania major* in Mediterranean Europe

VN MAP MARCH 21

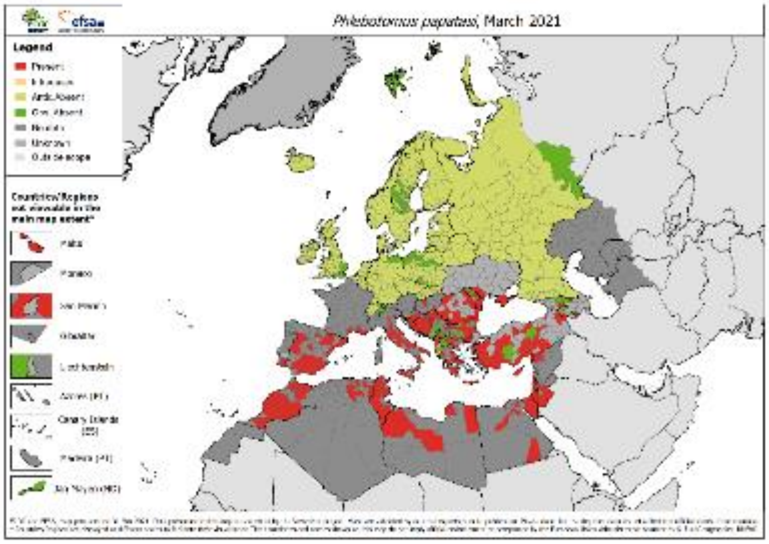

This species' range embraces Northern Africa, the Middle East, Turkey and Caucasus. It is also widely spread in every southern European country and in the Balkan peninsula and reported from Hungary and Ukraine, but *L. major* is not endemic in any of these areas, as they lack the necessary rodent reservoir species.

Comparison with VectorBase records (in Brown)

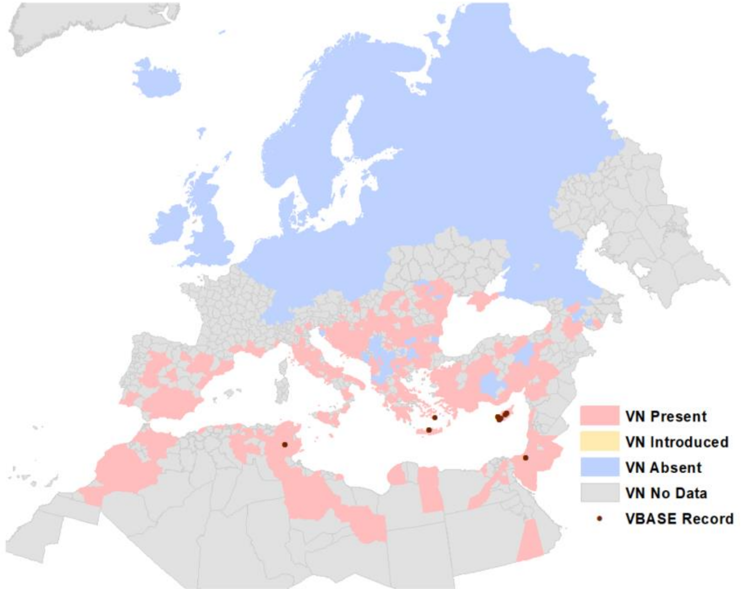

**VBASE Present**	**VN Present**	**VN Absent**	**VN No Data**	**Total**
**No**	305	535	661	**1501**
**Yes**	2	0	3	**5**
**Total**	**307**	**535**	**664**	**1506**

The VectorBase database only reports *P. papatasi* in North Africa Greece, Cyprus and the Middle East., all in areas where VectorNet also has records. Vectornet records extend significantly further than do the VectorBase ones

Supplementary Figure S34: ***Phlebotomus perfiliewi*** is a confirmed *L. infantum* vector and a dominant sand fly species in many areas

VN MAP MARCH 21

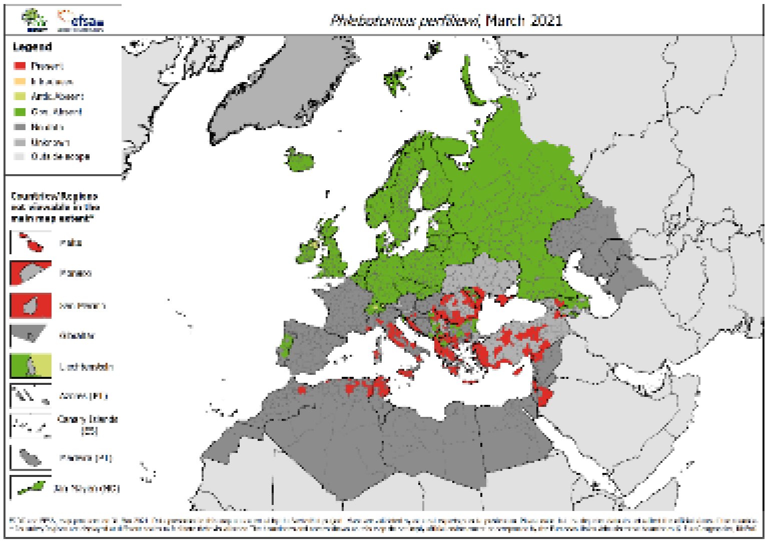

Like many other of this genus this species range is restricted to Italy, Eastern European countries and Turkey. Southeast France and the Caucasus constitute its eastern- and western-most limits in the study area, respectively. It has also been reported in Morocco, Algeria, Tunisia and the Middle East.

Comparison with GBIF records (in Brown)

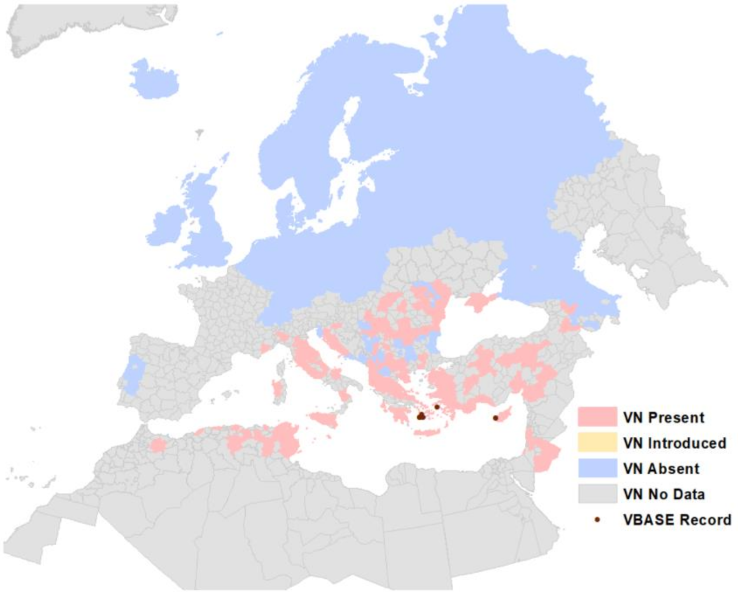

VectorBase records are only found for Greece and Cyprus, and match the Vectornet Records, which extend substantially further.

VBASE Present	VN Present	VN Absent	VN No Data	Total
**No**	184	554	765	**1503**
**Yes**	1	1	1	**3**
**Total**	**185**	**555**	**766**	**1506**

Supplementary Figure S35: ***Phlebotomus perniciosus*** is main leishmaniasis (*Leishmania infantum*) vector in Western Europe,

VN MAP MARCH 21

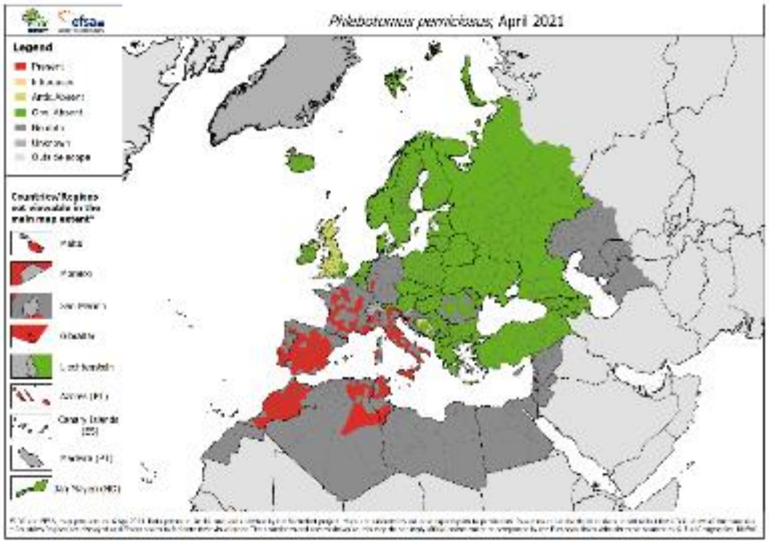

The species ranges from Portugal in the West to the Balkan Peninsula in the east, and it has been described as far north as Germany. In Northern Africa, *P. perniciosus* has also been reported in Morocco, Algeria and Tunisia only. Its altitudinal distribution is wide, having been found from sea level to over 1600m in the Pyrenees. Maps of the species' distribution in endemic countries is incomplete, as not all the territorial units have been reported to be sampled so far. Notwithstanding this, there are no similarly detailed distribution maps for Europe and neighbouring countries as those provided here.

Comparison with GBIF records (in Brown)

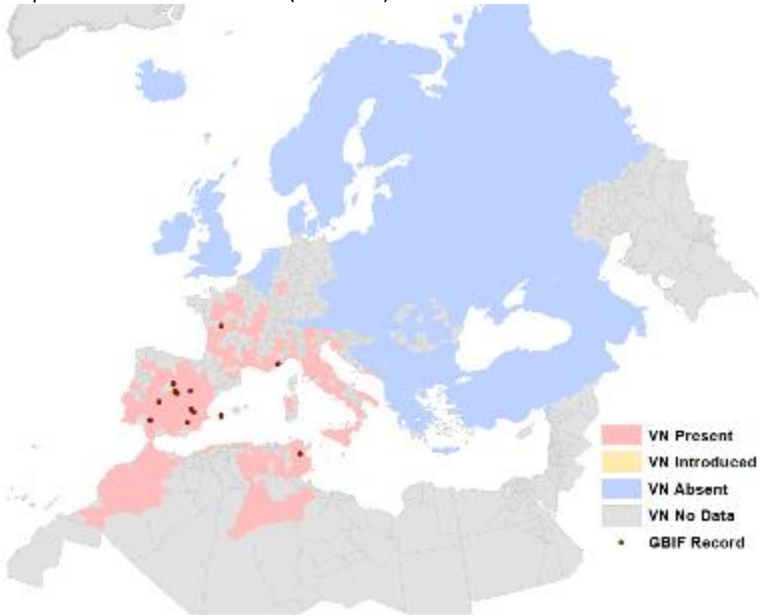

GBIF Present	VN Present	VN Absent	VN No Data	Total
**No**	97	1086	317	**1500**
**Yes**	1	4	1	**6**
**Total**	**98**	**1090**	**318**	**1506**

The GBIF distribution for this species includes many areas across Spain and isolated points in France and Tunisia, These are all within or very close to VectorNet presence areas.

Supplementary Figure S36: ***Phlebotomus sergenti*** is the most important vector of *Leishmania tropica* which is endemic in northern Africa, The Middle East and Turkey

VN MAP MARCH 2 1

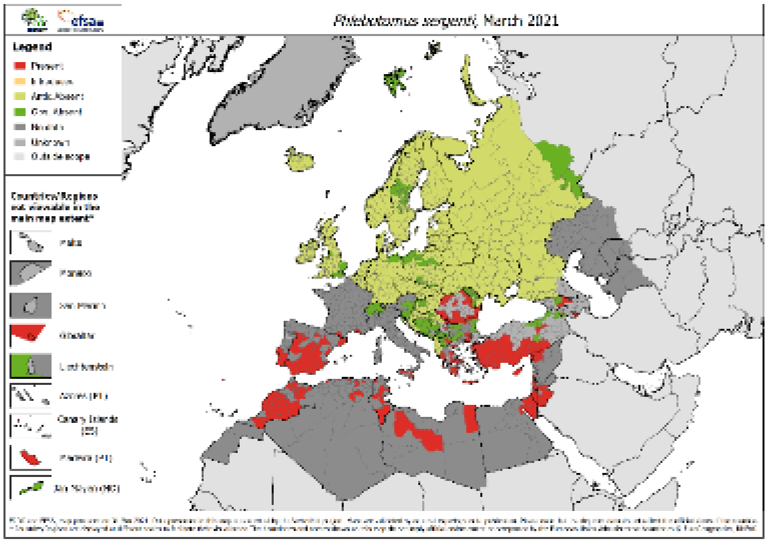

The vector is found in North Africa, the Middle East and Turkey, and is also widespread in the Iberian Peninsula, Sicily, Greece, Romania and the Caucasus. It has also been sporadically reported in other Balkan countries and Cyprus. *L. tropica*, the species transmitted by this sand fly, is indeed present in Northern Africa, Middle East and Turkey, but not in Europe.

Comparison with GBIF records (in Brown)

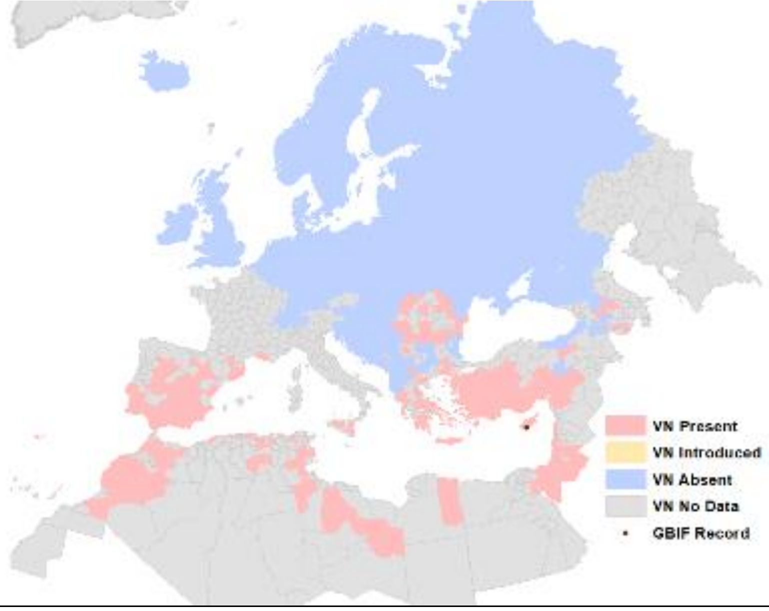

GBIF Present	VN Present	VN Absent	VN No Data	Total
**No**	193	648	664	**1505**
**Yes**	1	0	0	**1**
**Total**	**194**	**648**	**664**	**1506**

A single GBIF point for *P. sergenti* is shown in Cyprus that matches the Vectornet distribution

Supplementary Figure S37: *Phlebotomus similis* is considered to be a sister species of *P. sergenti* and responsible for *L. tropica* transmission in Crete

VN MAP MARCH 21

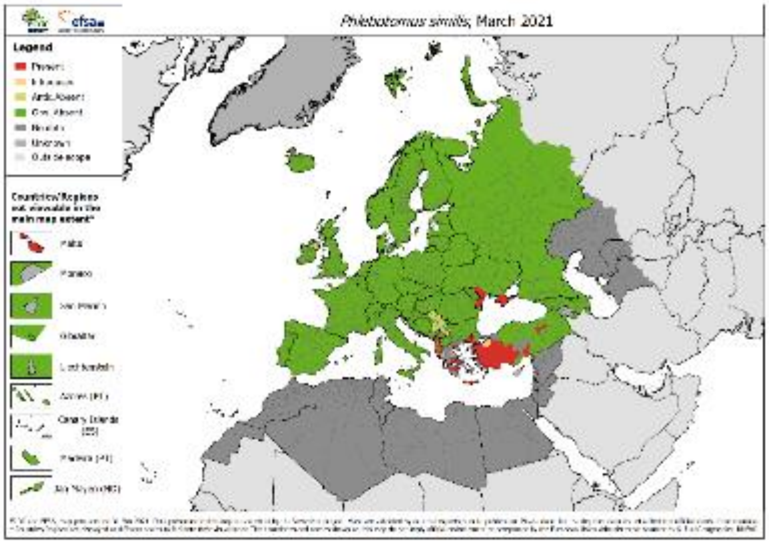

This sand fly has a very restricted distribution within the VectorNet region, and has been reported from mainland Greece, Crete, Cyprus, Albania, Western Turkey and Ukraine

Comparison with VectorBase records (in Brown)

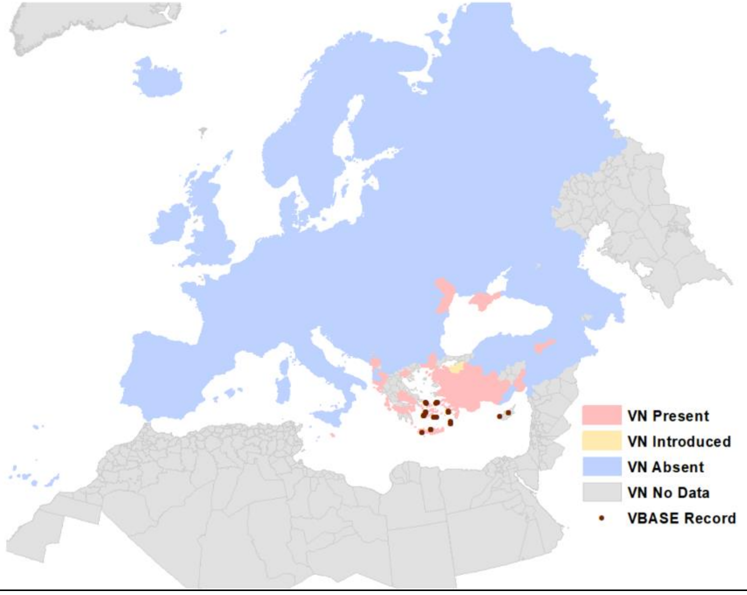

VectorBase shows a cluster of records in Greece and Cyprus which match the VectorNet mapped status, VectorNet has much more wide-ranging data than does VectorBase

VBASE Present	VN Present	VN Absent	VN No Data	Total
No	190	27	813	**1030**
Yes	142	0	334	**476**
**Total**	**332**	**27**	**1147**	**1506**

Supplementary Figure S38: *Phlebotomus tobbi* is a confirmed and abundant *Leishmania. infantum* vector

VN MAP MARCH 21

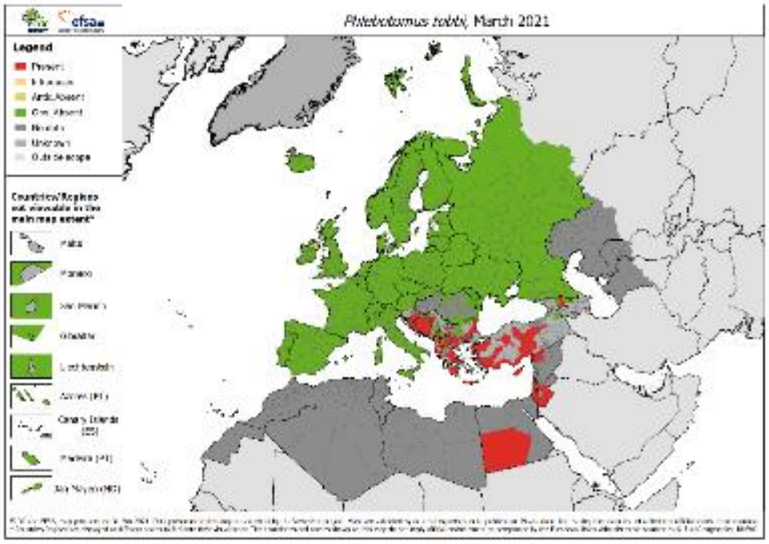

Reported only from the south- eastern parts of the VectorNet area, this species has been recorded from the Balkan Peninsula, Greece, Cyprus, the Middle East and Turkey, and has also been reported in the Caucasus and Egypt.

Comparison with GBIF records (in Brown)

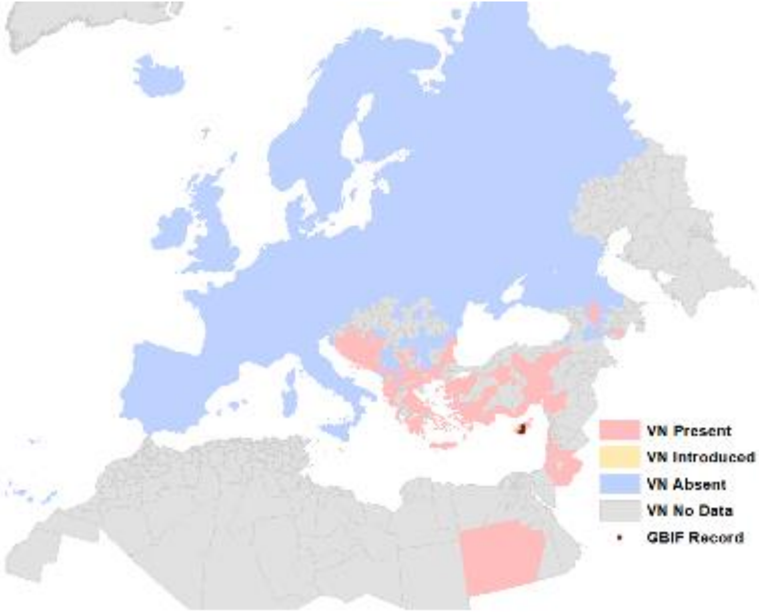

GBIF Present	VN Present	VN Absent	VN No Data	Total
**No**	115	883	507	**1505**
**Yes**	1	0	0	**1**
**Total**	**116**	**883**	**507**	**1506**

The only GBIF records are from Cyprus where VectorNet also records presence.

Supplementary Figure S39: *Dermacentor reticulatus*

Dermacentor reticulatus is known as the ornate cow tick. It frequently infests cattle and horses, and can also be found on dogs. It is generally considered a 'winter tick', active in northern Europe during the late winter / early spring, often before the other ticks become active. It can be found in central Europe in river valleys, and in north-western Europe in sand dune habitats and coastal grasslands. The tick is easily collected by flagging vegetation as well as from animal surveys. *D. reticulatus*, and similar species *D. marginatus* are considered competent vectors of CCHFV (cf Bazanov, B.A. *et al.* doi: 10.1089/vbz.2016.2075. epub 2017), TBEV and are important vectors of rickettsiae that cause TIBOLA/DEBONEL (*R. sibirica, R. slovaca*).

VN MAP MARCH 21

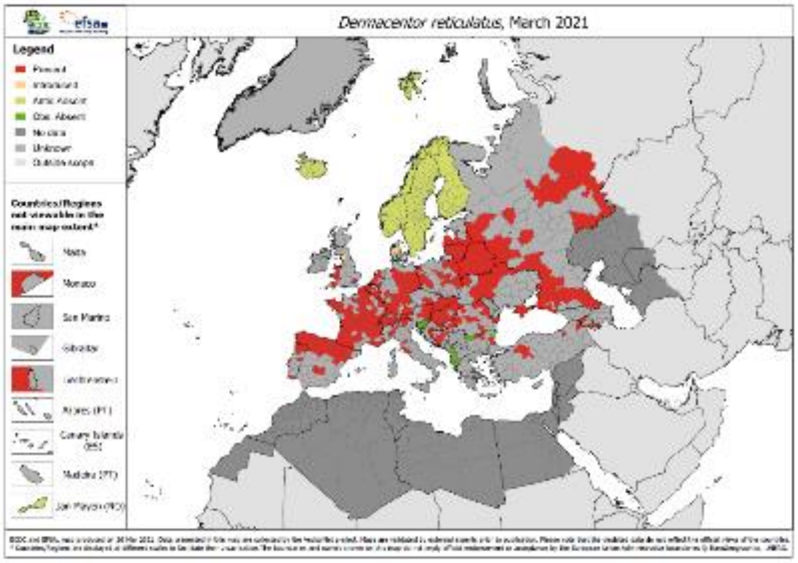

D. reticulatus is widely distributed across Europe, although its distribution is patchy. There are records from Denmark but not the rest of Scandinavia nor the Mediterranean, and the distribution in the British Isles is limited, with no records from Ireland. With it being a 'winter tick', it is not present in hot and arid climates.

Comparison with GBIF records (in Brown)

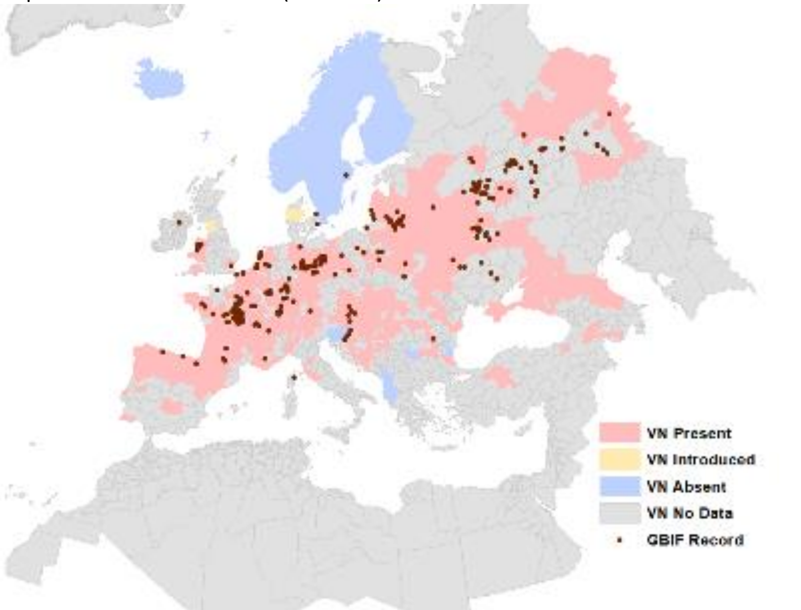

GBIF Present	VN Present	VN Absent	VN No Data	Total
**No**	258	174	972	**1404**
**Yes**	70	1	31	**102**
**Total**	**328**	**175**	**1003**	**1506**

There are many records of *D. reticulatus* in GBIF, and the distribution of these records are generally similar to those of VectorNet but with about a third in No Data in Eastern Europe and Russia though within what can be inferred at the species' range. Only one GBIF record – in Sweden- is in an area that VectorNet records as absent.

Supplementary Figure S40: *Hyalomma lusitanicum*

Hyalomma lusitanicum is a tick of the western Mediterranean. It has recently been implicated in the transmission of CCHFV in Spain. It can be found in woodland, steppe and scrub habitat in Iberia. It adopts a hunter strategy, like other *Hyalomma* ticks. It feeds on a variety of large and medium sized mammals such as wild and domestic ungulates. Immature stages feed on lagomorphs, and rarely birds, though it is transported on dogs.

VN MAP MARCH 21

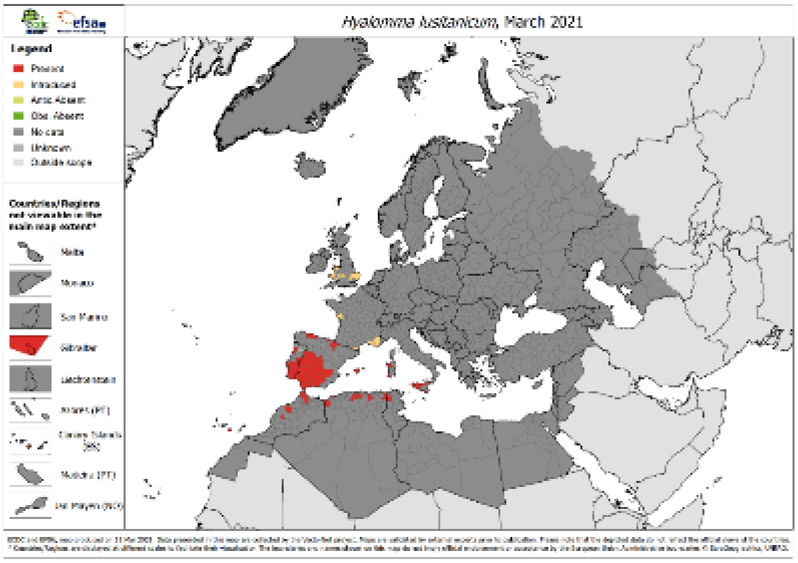

This tick is largely restricted to the western Mediterranean, primarily Iberia and North African coast fringe. Northerly records are imported ticks on dogs, that have travelled to Iberia.

Comparison with GBIF records (in Brown)

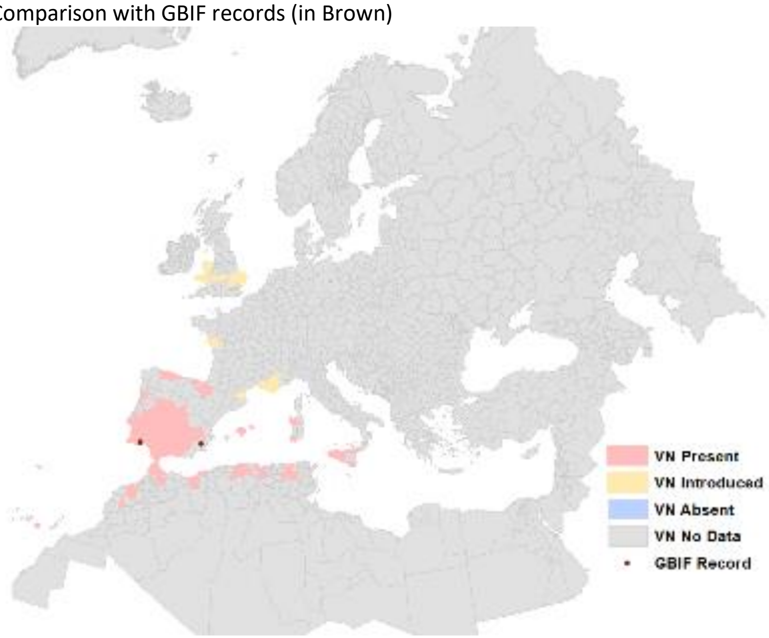

GBIF has very few records of *H. lusitanicum*, and they are all in Iberia, in or very close to the VectorNet presence reports.

GBIF Present	VN Present	VN Absent	VN No Data	Total
No	65	0	1438	**1503**
Yes	1	0	2	**3**
**Total**	0	**0**	**1440**	**1506**

Supplementary Figure S41: *Hyalomma marginatumCCHF*

Hyalomma marginatum is the primary European vector of CCHFV. Adult stages are frequently found on cattle where it has a predisposition to biting around the perineum and underbelly. They also often bite humans. The immature stages are found on birds and lagomorphs. The immature stages are nidifugous and are often carried by migratory birds to distant (northern) locations. Here, the ability of the engorged nymph to moult to the adult stage is climate dependent. This species does not quest so are normally sampled by examining the animal hosts, rather than more traditional flagging techniques used for other tick species such as *Ixodes* and *Dermacentor*. In addition, in contrast to other genera, including *Ixodes*, the *Hyalomma* ticks can survive in more arid environments, and are not restricted to humid environments, often afforded by forested habitats.

VN MAP MARCH 21

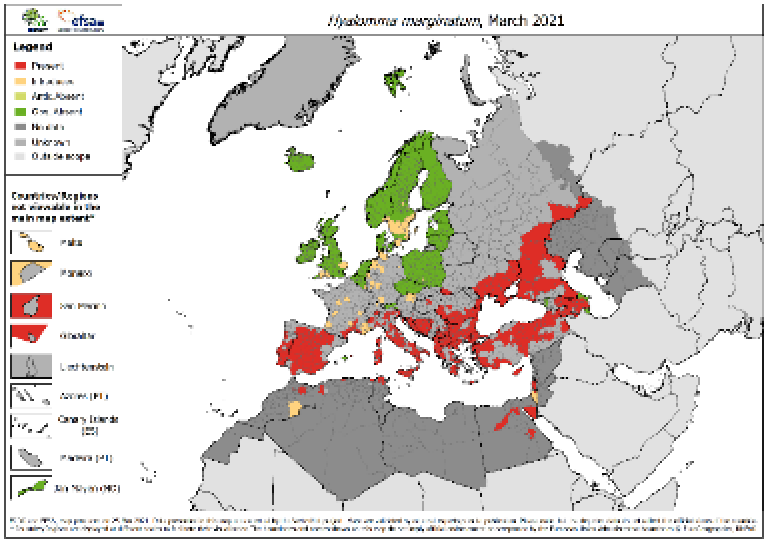

The presence records of *Hyalomma marginatum* are limited to the warmer climates of southern Europe and the Mediterranean. All records in more northerly locations (represented here in yellow) are records of imported ticks. They are frequently reported on migratory birds. During the heatwave summer 2018, nymphs were able to complete their temperature-dependent moult and many adults were collected in these northerly areas. However, this does not mean that they are established.

Comparison with GBIF records (in Brown)

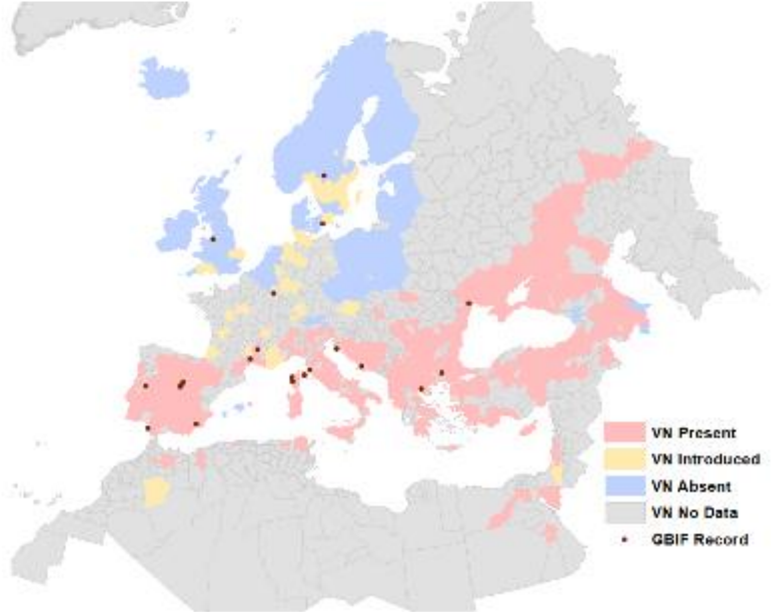

GBIF Present	VN Present	VN Absent	VN No Data	Total
**No**	347	352	790	**1489**
**Yes**	13	3	1	**17**
**Total**	**360**	**355**	**791**	**1506**

There are few records of this tick in GBIF most of which match the VectorNet distributions. Their distribution illustrates the challenge in mapping records of *H. marginatum*. The records in southern Europe likely represent data from established populations, and their records were probably from samples from large animal hosts. The records in the VectorNet absent areas in northern Europe, suggest that the tick is found across Europe. However, it is more likely that these are imported ticks and do not constitute established populations.

Supplementary Figure S42: *Ixodes persulcatus*

Ixodes persulcatus is very similar morphologically to *Ixodes ricinus*, as it occurs in similar woodland habitats though it is found in more grassy areas. Its populations tend to peak earlier in the year. It is also a vector of *Borrelia burgdorferi s.l.* and TBEV, along with other tick-borne pathogens such as *Anaplasma*.

VN MAP MARCH 21

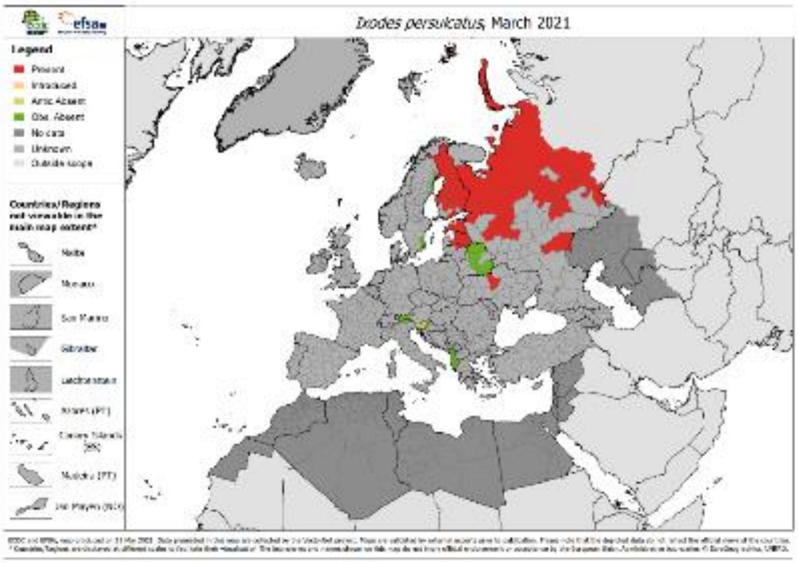

This tick species has a restricted distribution in western Russia, Finland and the Baltics states. It is likely that its morphological and habitat similarities to *I. ricinus* might make it under-recorded in fringe areas. It was recently detected in northern Sweden.

Comparison with GBIF records (in Brown)

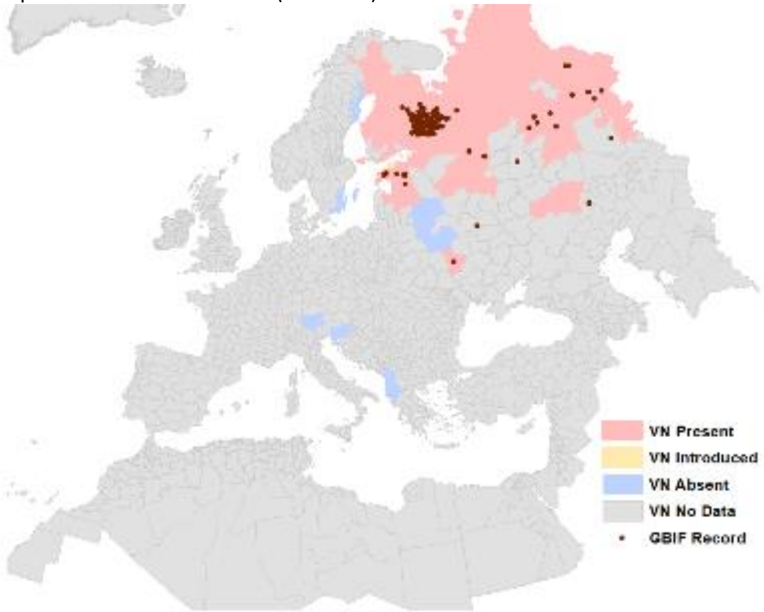

GBIF Present	VN Present	VN Absent	VN No Data	Total
**No**	35	36	1419	**1490**
**Yes**	12	0	4	**16**
**Total**	**47**	**36**	**1423**	**1506**

Most of the GBIF records match the VectorNet presence reports or occur within the inferable distributional range of VectorNet records. The intensity of recording in western Russia/Finland is indicative of its main range.

Supplementary Figure S43: ***Ixodes Ricinus***

Ixodes ricinus is widespread across Europe. Principally a tick of habitats that afford a moist microclimate like forests, it is found in grazed grasslands, moorland/heathland/montane habitats, mosaic habitats with agriculture, grassland and pasture, as well as urban parks. It's ubiquity and wide host range makes it an ideal vector for several pathogens. Larvae, nymphs and adults can be found on a wide range of animal host. Humans are often bitten, as are companion animals such as dogs and cats, and so records of tick bites from these species are numerous. In more sylvatic habitats, all stages of tick can be found on large ungulates such as deer, cattle and sheep, with the immature tick stages infesting a range of small and medium sized mammals and birds. It is the primary vector of *Borrelia burgdorferi* (the causative agent of Lyme borreliosis), Tick-borne encephalitis virus and other pathogens such as *Babesia, Anaplasma, Borrelia miyamotoi*, *Ricketssia* and Louping ill virus.

VN MAP MARCH 21

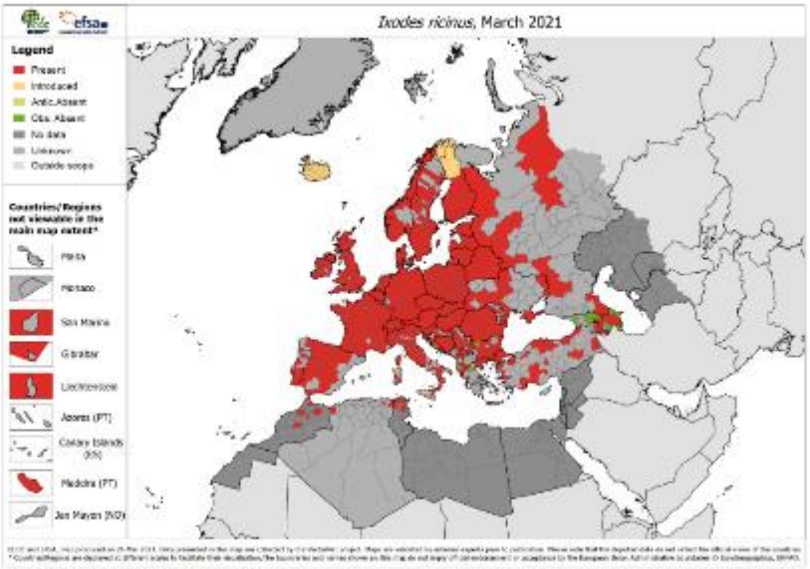

Comparison with GBI (in Yellow)

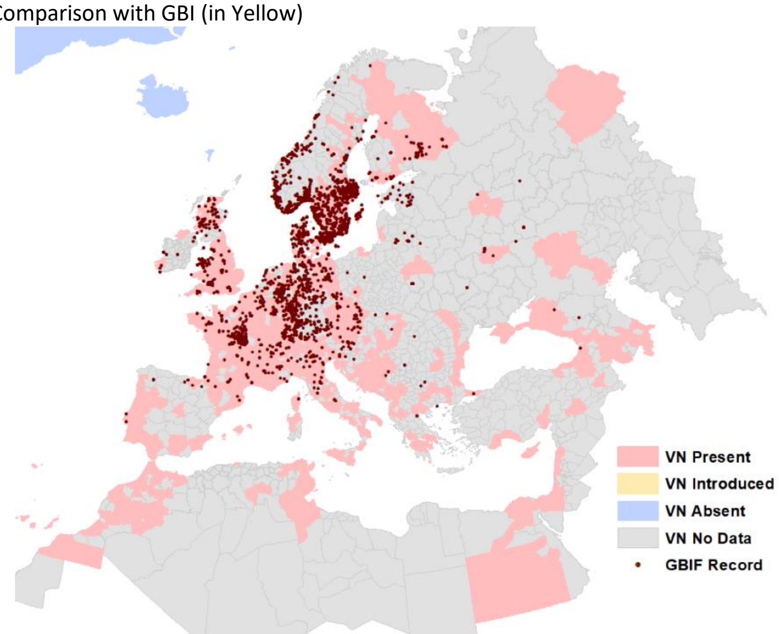

GBIF Present	VN Present	VN Absent	VN No Data	Total
**No**	579	17	595	**1191**
**Yes**	290	0	25	**215**
**Total**	**869**	**17**	**620**	**1506**

The distribution is essentially pan-regional, but acquired records are biased to west central and southern areas, with relatively few data records from eastern regions, so extrapolations are likely to be less accurate to east. Records of introduction (yellow) are seen in the far north of its range. There are few absence records.

Ixodes ricinus is limited in its distribution where there is an absence of hosts or a microclimate that limits their survival. For example, southern parts of Europe are too dry for their survival. High altitude areas of the Alps of central Europe are too cold for their survival, although they are being found at high altitude. In northern regions, there is a climatic limit, but the absence of abundant hosts is a limiting factor in places like Iceland.

This species is one of the most prolifically recorded by GBIF. Ninety percent of the GBIF occurrences are in VectorNet areas defined as present with about ten percent in areas with No Data, most noticeably in Norway and to a lesser extent in Russia and the Baltic countries. There are no GBIF records in VectorNet polygons defined as absent.

Ixodes ricinus remains one of the best recorded species of tick in Europe. For some records in southern Europe, these are now being questioned as other similar species (*I. gibbosus. I. inopinatus*).

Supplementary Figure S44: *Ornithodorus erraticus*

The *Ornithodorus erraticus* complex includes a number of similar species and is a subject of much debate. They are associated with warm blooded animals, such as ungulates, carnivores, rodents and insectivores. Species of the *O. erraticus* complex are often found on pigs, with some experimental evidence of a role in the transmission of African swine fever virus.

VN MAP MARCH 21

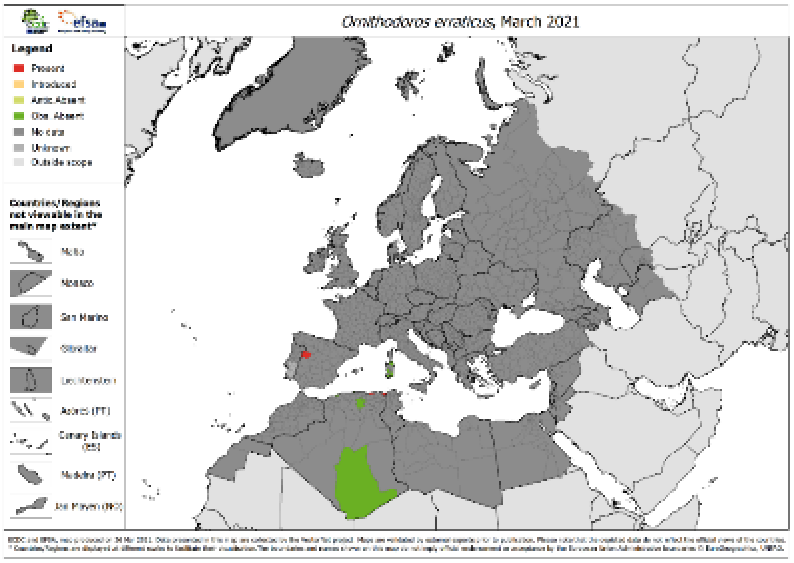

There is little published data on the distribution of these ticks, and they are generally under-recorded. The species of wider *Ornithodoros* genus are also not widely recorded as many are specialist species of wildlife and livestock, including some species associated with seabirds. Unlike the Ixodidae that spend time in vegetation, questing for a host, these species tend to be more nidicolous and therefore are not easily collected during routine tick sampling.

Comparison with GBIF records (in Brown)

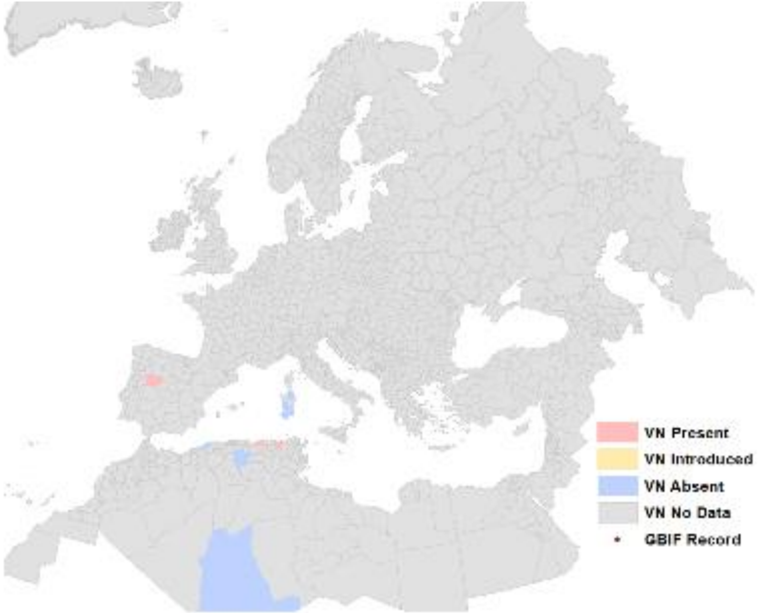

GBIF Present	VN Present	VN Absent	VN No Data	Total
No	3	4	1499	**1506**
Yes	0	0	0	**0**
**Total**	**3**	**4**	**1499**	**1506**

There are no GBIF Records. The lack of data is indicative of the paucity of records of *O. erraticus*.

Supplementary Figure S45: *Rhipicephalus sanguineus* group

R. sanguineus s.l. (the brown dog tick) has two defined lineages, one that is temperate, the other tropical. They are primarily ticks of dogs, particularly in urban areas, and are one of the most common ticks on dogs globally, associated with their homes and shelters. They can be found in the cracks and crevices on walls of rooms where dogs spend the night and have an ability to survive indoors in cooler climes. *R. turanicus* is morphologically similar to *R. sanguineus*, although it is more of a sylvatic species in grassland and meadows, semi-desert and steppe habitats, feeding on a range of mammals and birds, with a possible role in rickettsial transmission. The brown dog tick can transmit both human and animal diseases such as Mediterranean spotted fever and canine babesiosis and ehrlichiosis.

VN MAP MARCH 21

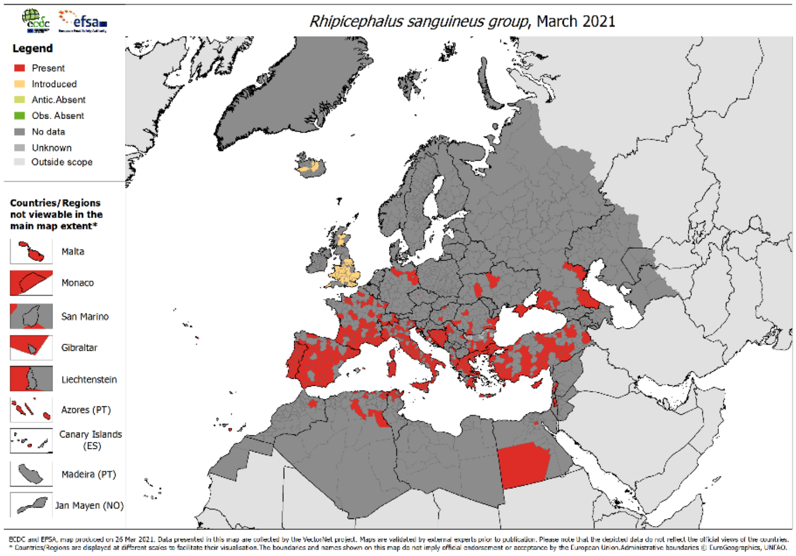

Rhipicephalus sanguineus is a common tick of dogs, occurring right across western, central and southern Europe and the Mediterranean. It is normally associated with the housing and bedding of dogs. In more northerly parts of Europe, it has been detected on travelling dogs where it can subsequently infest properties despite the cooler climate. Many of the introduced records from northern Europe are on imported dogs, or found infesting houses that have imported dogs.

Comparison with GBIF records (in Brown)

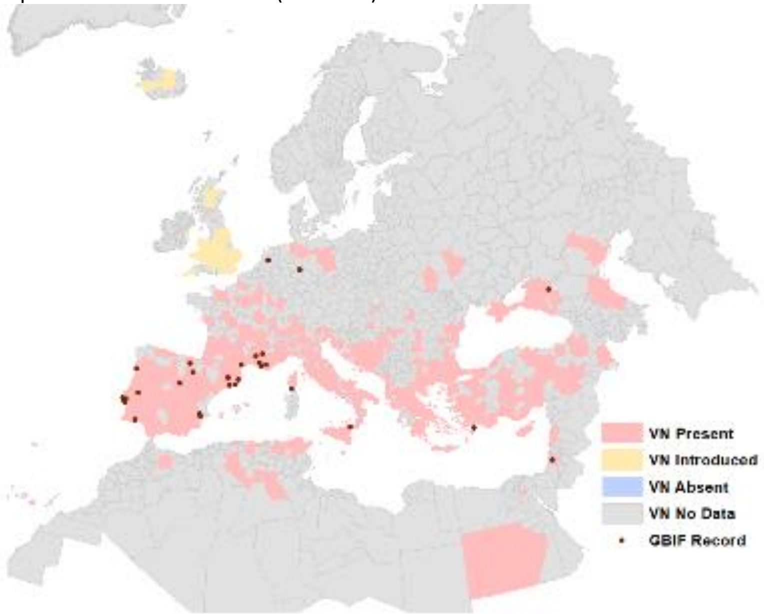

GBIF Present	VN Present	VN Absent	VN No Data	Total
No	336	0	1145	**1481**
Yes	18	0	7	**25**
**Total**	**354**	**0**	**1152**	**1506**

The records in GBIF largely match the distribution of this tick species in VectorNet, which is primarily in southern and Mediterranean regions. More northerly records are unlikely to reflect local established populations, but rather imported ticks on travelling dogs, or importations that subsequently lead to localised house infestations.

SUPPLEMENTRAY INFORMATION SECTION VI: Supplementary Figure S46: Citations

Supplementary Figure S46: Citation Numbers to November 2021

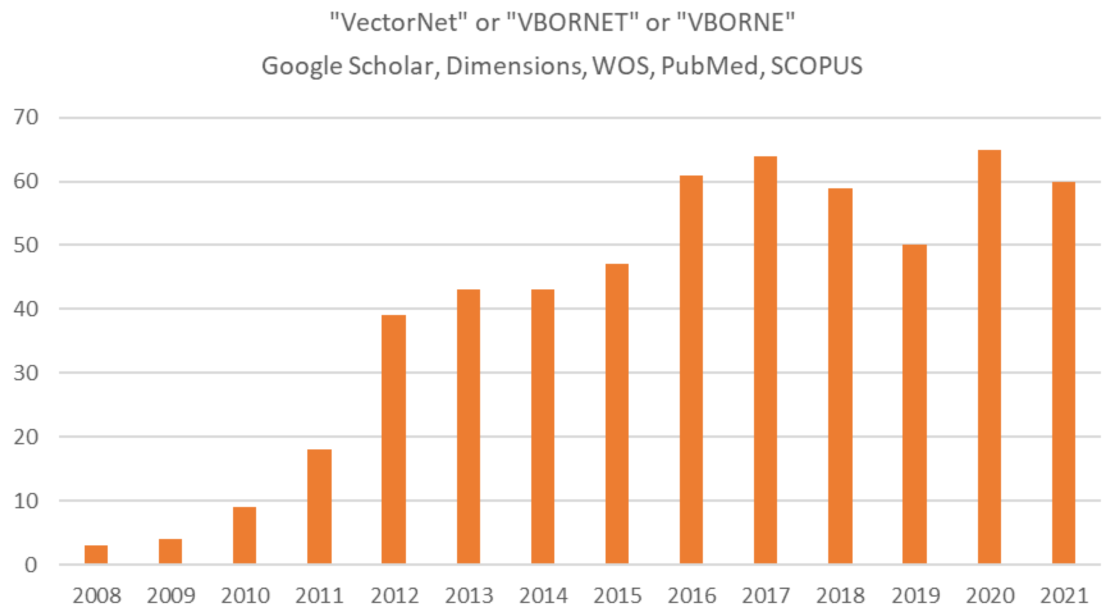

Supplement: Supplementary Material [file 22-00666_BRIET_Supplement.pdf]
